# TLR7 promotes smoke-induced experimental lung damage through the activity of mast cell tryptase

**DOI:** 10.1038/s41467-023-42913-z

**Published:** 2023-11-14

**Authors:** Gang Liu, Tatt Jhong Haw, Malcolm R. Starkey, Ashleigh M. Philp, Stelios Pavlidis, Christina Nalkurthi, Prema M. Nair, Henry M. Gomez, Irwan Hanish, Alan CY. Hsu, Elinor Hortle, Sophie Pickles, Joselyn Rojas-Quintero, Raul San Jose Estepar, Jacqueline E. Marshall, Richard Y. Kim, Adam M. Collison, Joerg Mattes, Sobia Idrees, Alen Faiz, Nicole G. Hansbro, Ryutaro Fukui, Yusuke Murakami, Hong Sheng Cheng, Nguan Soon Tan, Sanjay H. Chotirmall, Jay C. Horvat, Paul S. Foster, Brian GG. Oliver, Francesca Polverino, Antonio Ieni, Francesco Monaco, Gaetano Caramori, Sukhwinder S. Sohal, Ken R. Bracke, Peter A. Wark, Ian M. Adcock, Kensuke Miyake, Don D. Sin, Philip M. Hansbro

**Affiliations:** 1 Centre for Inflammation, Centenary Institute, and Faculty of Science, University of Technology Sydney, Camperdown, New South Wales Australia; 2 Immune Healthy &/or Grow Up Well, Hunter Medical Research Institute & University of Newcastle, Callaghan, New South Wales Australia; 3 Depatrment of Immunology and Pathology, Central Clinical School, Monash University, Melbourne, Victoria Australia; 4 School of Clinical Medicine, UNSW Medicine and Health, St Vincent’s Healthcare clinical campus, UNSW, Sydney, Australia; 5 The Airways Disease Section, National Heart & Lung Institute, Imperial College London, London, UK; 6 Department of Microbiology, Faculty of Biotechnology and Biomolecular Sciences, Universiti Putra Malaysia, Serdang, Selangor Malaysia; 7 Department of Medicine, Baylor College of Medicine, Houston, Texas USA; 8 Department of Radiology, Brigham and Women’s Hospital, Harvard Medical School, Boston, USA; 9 School of Life Sciences, Faculty of Science, University of Technology Sydney, Sydney, Australia; 10 Division of Innate Immunity, Department of Microbiology and Immunology, The Institute of Medical Science, The University of Tokyo, Shirokanedai, Minatoku, Tokyo, Japan; 11 Faculty of Pharmacy, Department of Pharmaceutical Sciences, Musashino University, Nishitokyo-shi, Tokyo, Japan; 12 Lee Kong Chian School of Medicine, Nanyang Technological University, Singapore, Singapore; 13 School of Biological Sciences, Nanyang Technological University, Singapore, Singapore; 14 Department of Respiratory and Critical Care Medicine, Tan Tock Seng Hospital, Singapore, Singapore; 15 Woolcock Institute of Medical Research, University of Sydney & School of Life Sciences, University of Technology, Sydney, Australia; 16 Department of Human Pathology in Adult and Developmental Age “Gaetano Barresi”, Section of Anatomic Pathology, Università di Messina, Messina, Italy; 17 Thoracic Surgery, Dipartimento di Scienze Biomediche, Odontoiatriche e delle Immagini Morfologiche e Funzionali (BIOMORF), Università di Messina, Messina, Italy; 18 Pneumologia, Dipartimento BIOMORF and Dipartimento di Medicina e Chirurgia, Universities of Messina and Parma, Messina, Italy; 19 Respiratory Translational Research Group, Department of Laboratory Medicine, School of Health Sciences, University of Tasmania, Launceston, Australia; 20 Laboratory for Translational Research in Obstructive Pulmonary Diseases, Department of Respiratory Medicine, Ghent University Hospital, Ghent, Belgium; 21 The University of British Columbia Centre for Heart Lung Innovation, St Paul’s Hospital & Respiratory Division, Dept of Medicine, University of British Columbia, Vancouver, BC Canada

**Keywords:** Chronic inflammation, Toll-like receptors, Chronic obstructive pulmonary disease, Inflammatory diseases

## Abstract

Toll-like receptor 7 (TLR7) is known for eliciting immunity against single-stranded RNA viruses, and is increased in both human and cigarette smoke (CS)-induced, experimental chronic obstructive pulmonary disease (COPD). Here we show that the severity of CS-induced emphysema and COPD is reduced in TLR7-deficient mice, while inhalation of imiquimod, a TLR7-agonist, induces emphysema without CS exposure. This imiquimod-induced emphysema is reduced in mice deficient in mast cell protease-6, or when wild-type mice are treated with the mast cell stabilizer, cromolyn. Furthermore, therapeutic treatment with anti-TLR7 monoclonal antibody suppresses CS-induced emphysema, experimental COPD and accumulation of pulmonary mast cells in mice. Lastly, *TLR7* mRNA is increased in pre-existing datasets from patients with COPD, while TLR7^+^ mast cells are increased in COPD lungs and associated with severity of COPD. Our results thus support roles for TLR7 in mediating emphysema and COPD through mast cell activity, and may implicate TLR7 as a potential therapeutic target.

## Abstract (web summary)

Toll-like receptor 7 (TLR7) normally recognizes exogenous single-stranded RNA for the activation of innate immunity. Here the authors show that TLR7 may also contribute, via the modulation of mast cell functions, to experimental, cigarette smoke-induced mouse models of emphysema, thereby hinting TLR7 as a potential therapeutic target for human lung inflammation.

## Introduction

Lung damage in respiratory disease is the most prolific cause of illness and death globally. Chronic obstructive pulmonary disease (COPD) is the third leading cause of mortality worldwide and imposes an enormous socioeconomic burden^1^. It is complex and heterogeneous characterized by chronic pulmonary inflammation, airway remodeling, emphysema, and progressively declining lung function^2^. A major risk factor is cigarette smoke (CS) inhalation whilst other exposures such as wood smoke and air pollution are also important^3^. Emphysema is associated with airway inflammation and progressive disease irrespective of smoking status^4^. Current therapies include smoking cessation, glucocorticoids, β2-adrenergic agonists, and anticholinergic agents^5–7^. However, they only provide some symptomatic relief, and do not suppress causal factors, reverse the disease or halt its progression^7^. Hence, COPD lacks effective treatments, which is largely due to the poor understanding of underlying disease mechanisms.

Toll-like receptor (TLR)7 is an intracellular pattern recognition receptor (PRR) with well-known roles in host defense against single-stranded (ss)RNA viruses including influenza A and SARS-CoV-2 virus^8–12^. When single-stranded (ss)RNA interacts with TLR7, myeloid differentiation primary response gene (MyD)88 is recruited. This leads to the activation of nuclear factor kappa-light-chain-enhancer of activated B cells (NF-κB) that drives inflammatory responses^13,14^. TLR7 may also signal through TIR-domain-containing adapter-inducing interferon (IFN)-β (TRIF) to activate IFN regulatory factors (IRFs), which drive the production of anti-viral type-I IFNs^13,14^.

In the lung, TLR7 has pathogenetic roles in asthma^15–18^. TLR7 agonists suppressed levels of pro-inflammatory cytokines (interleukin [IL]−4, IL-5, IL-13) and airway inflammation, fibrosis and hyperresponsiveness in experimental asthma^15–18^. Mast cells are important inflammatory cells in asthma and COPD^19–23^. They express several PRRs, including TLR7^24–26^, as well as cell-specific tetramer-forming tryptases (e.g., mouse MC protease-6 [mMCP-6], human (h)Tryptase-β)^27,28^. However, the role of TLR7 in COPD/emphysema is unknown, and there are no known links between TLR7 and mast cell-specific mediators.

Understanding the function of TLR7 in COPD is key to identifying new therapeutic options to COPD. In this study, we show that TLR7 mRNA and protein are increased in both human and experimental COPD. TLR7-deficient (*Tlr7*^−/−^) mice are protected against CS-induced emphysema-like alveolar enlargement associated with attenuated airway remodeling, apoptosis and mast cell numbers in the lungs of a mouse model of experimental COPD. The TLR7-agonist, imiquimod, increases CS-induced emphysema that is reduced in *mmcp6*^−/−^ mice, or in wild-type mice treated with the mast cell stabilizer cromolyn. CS-induced emphysema, apoptosis, airway remodeling, and mast cells are reduced in mice following therapeutic neutralization of TLR7. Thus, we identify a role for TLR7 in COPD pathogenesis and its potential as a therapeutic target.

## Results

### TLR7 is increased in human and experimental COPD

**Fig. 1:**
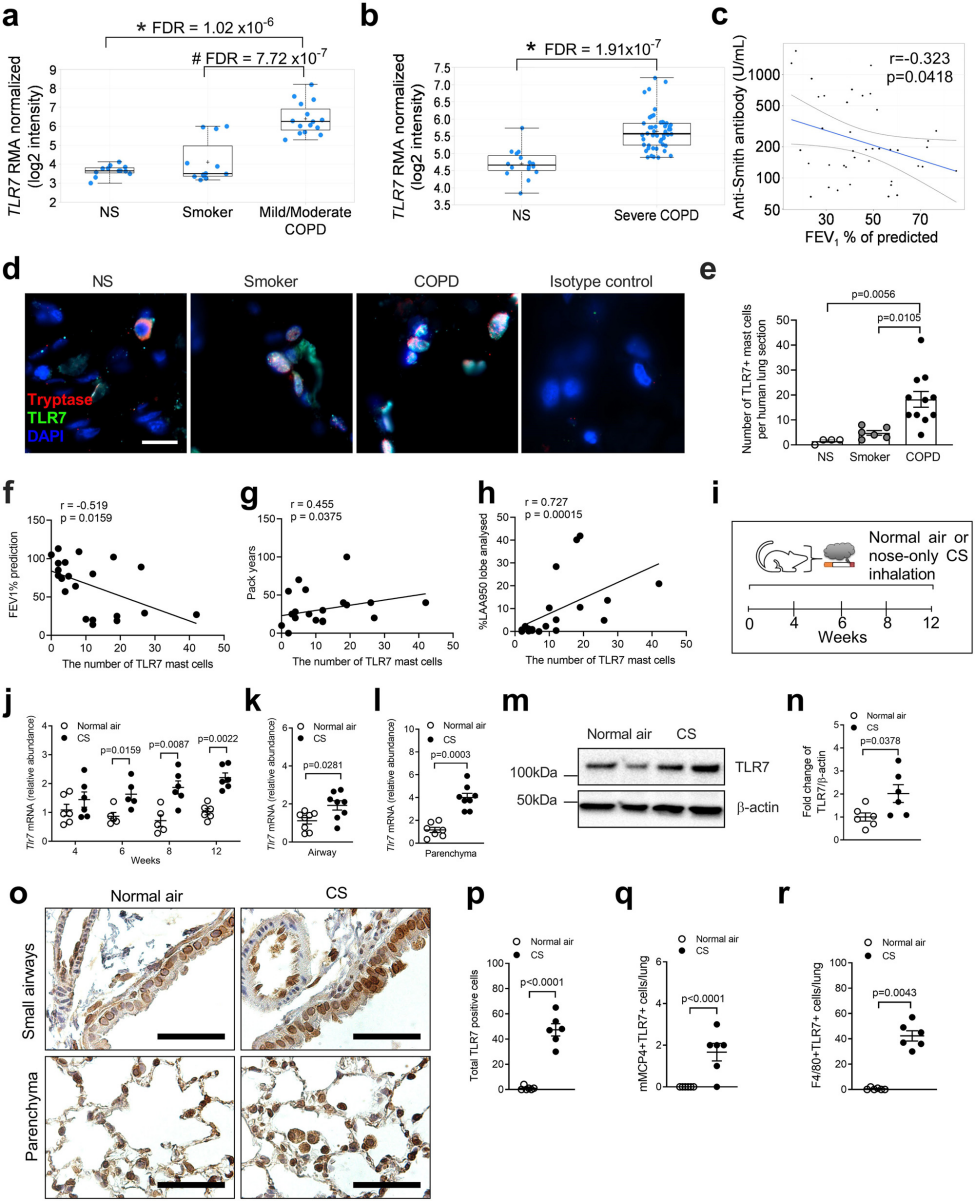
TLR7 is increased in human and experimental COPD. **a** *TLR7* mRNA levels in airway epithelial brushings from non-smokers (NS), healthy smokers without COPD (Smoker) and COPD patients with Global Initiative for Chronic Obstructive Lung Disease (GOLD) stage I (mild) or II (moderate) disease (*n* = 12 NS; *n* = 12 Smokers; *n* = 15 mild or moderate COPD). **b** *TLR7* mRNA levels in lung parenchyma cores from NS and COPD patients with GOLD stage IV (severe) disease (*n* = 16 NS; *n* = 48 severe COPD). Differential gene expression analysis was performed using published microarray datasets (GEO accession numbers GSE5058 and GSE27597) and the numbers in panels **a** and **b** represent the false discovery rate (FDR), whereby *denotes FDR of COPD *vs*. NS; and ^#^ denotes FDR of COPD *vs*. Smoker. The data are presented as box and whiskers with min to max showing all points. **c** Correlation analysis of anti-Smith antibody levels in serum and forced expiratory volume in 1 second (FEV_1_) of mild-to-moderate COPD patients (*n* = 40). **d** Human lung sections stained with tryptase, TLR7 and DAPI by immunofluorescence, and **e** TLR7^+^ mast cells were enumerated in sections from NS controls (*n* = 4), smoker (*n* = 6) and COPD patients (*n* = 11). The numbers of TLR7^+^ mast cells correlated with **f** FEV_1_% predicted, **g** pack years of cigarettes, and **h** low attenuation areas less than a threshold of −950 Hounsfield units (%LAA950) in NS, smoker, and COPD patients. **i** Induction of experimental COPD where wild-type (WT) BALB/c mice (female, 6–8 weeks old) were exposed to nose-only inhalation of cigarette smoke (CS) for up to 12 weeks, controls received normal air. **j** *Tlr7* mRNA levels in whole lungs of WT mice exposed to normal air or CS after 4, 6, 8, and 12 weeks (*n* = 6 mice per group). *Tlr7* mRNA levels in blunt-dissected **k** airways and **l** lung parenchyma after 8 weeks of CS exposure (*n* = 6 mice per group). Wild-type (WT) BALB/c mice (*n* = 6) were exposed to CS for 8 weeks to induce experimental COPD, controls were exposed to normal air. **m** TLR7 protein was assessed in mouse lungs by immunoblot, and **n** quantitated by densitometry analysis of fold change normalised to β-actin (*n* = 6 mice per group). **o** Representative micrographs (*n* = 3 mice per group) of TLR7 immunostaining in small airways (top) and lung parenchyma (bottom) of WT mice exposed to normal air (left) or CS (right) for 8 weeks. Scale bars, 50 µm. WT BALB/c mice were exposed to 8 weeks of CS, control mice breathed normal air. **p** Total TLR7^+^ cells, **q** mMCP4^+^TLR7^+^ mast cells and **r** F4/80^+^TLR7^+^ macrophages enumerated in whole lung sections (*n* = 6 mice per group). All data are presented as means ± s.e.m. and are representative of two independent experiments. For panel **c**, **f**–**h**, correlation analyzes were performed using Spearman’s rank correlation coefficient test. For panel **e**, compared to NS or smokers using one-way ANOVA with Bonferroni’s multiple comparison test. The rest of the panels compared COPD to normal air-exposed controls using a two-tailed Mann–Whitney test. Source data are provided as a Source Data file.

We first assessed *TLR7* mRNA levels in pre-existing human microarray data from the global initiative for chronic obstructive lung disease (GOLD) stage I (mild), II (moderate) and IV (severe) COPD and non-COPD participants (Supplementary Tables 1–3)^29–31^. *TLR7* mRNA was not different in airway epithelial brushings^30^ from healthy smokers without COPD compared to non-smokers (Fig. 1a). However, it was increased in airway epithelial brushings from mild-to-moderate COPD patients compared to non-smokers and healthy smokers without COPD. *TLR7* mRNA was also increased in lung parenchyma^29,31^ from severe COPD patients compared to participants without COPD (Fig. 1b). We stained TLR7 proteins in the airways from patients with moderate (GOLD stage II) or severe COPD (GOLD stage IV), non-COPD smoker and control donor (Supplementary Table 4). TLR7 expression was increased in epithelial cells from severe compared to moderate COPD patients (Supplementary Fig. 1). To further strengthen the human data we performed immunohistochemical analysis of TLR7 expression in the peripheral lung from severe COPD and control donors (Supplementary Table 5). This showed substantially increased TLR7 protein levels in immune cells, peripheral lung tissue, and alveolar epithelial cells in COPD patients compared to smokers with normal lung function (Supplementary Fig. 2). It also showed that TLR7 was both membranous and cytoplasmic. Endogenous host RNA may induce TLR7 to drive disease^32,33^. Thus, we assessed the levels of anti-Smith antibody against endogenous RNA^34^. Levels inversely correlated with lung function impairment in 40 COPD patients (forced expiratory volume in 1 second [FEV_1_] <80% of predicted, FEV_1_ to FVC ratio <0.70 post-bronchodilator^35^, Fig. 1c, Supplementary Table 6).

We further examined differential transcriptomes in these existing datasets (GSE27597) between lung biopsies of healthy and COPD donors^29^. We identified 4269 differentially expressed genes (DEGs) of 3 gene clusters linked to immune responses (1875 DEGs), tissue remodeling (1009 DEGs), and metabolic reprogramming (1365 DEGs). TLR7 is part of the immune response cluster that is over-expressed in COPD. We then used pairwise Pearson correlation analysis to produce a gene signature with a strong positive correlation with *TLR7* (Pearson r ≥ 0.6; FDR < 0.05). This was consistently upregulated in COPD samples in an independent dataset (GSE5058)^30^, demonstrating its importance in COPD (Supplementary Fig. 3). A subset of the signature was overexpressed in COPD airway organoids^36^ associated with inflammatory responses and cytokine production, suggesting that other cells such as immune cells including mast cells, are responsible for leukocyte activation and differentiation. Analysis of human single-cell RNA-sequencing COPD datasets confirmed that *TLR7* expression was increased in mast cells in COPD patients (Supplementary Fig. 4).

We found that TLR7^+^ mast cells were increased in COPD patients (Supplementary Table 7) compared to controls (Fig. 1d, e). Furthermore, increased TLR7^+^ mast cell numbers correlated with impairment of lung function and decreased predicted FEV_1_ (Fig. 1f), increased pack years of cigarette smoking (Fig. 1g), and increased low attenuation areas less than a threshold of −950 Hounsfield units (%LAA950, Fig. 1h). Together these results indicate that the numbers of TLR7^+^ mast cells are associated with the severity of COPD.

We next assessed *Tlr7* mRNA levels in the lungs in experimental COPD. Wild-type (WT) BALB/c mice were exposed to nose-only inhalation of CS or normal air and euthanised with pentobarbitone overdose after 4, 6, 8, and 12 weeks (Fig. 1i) as described previously^27,28,37–47^. *Tlr7* mRNA was increased after 6, 8, and 12 weeks of CS exposure (Fig. 1j), in both airways and lung parenchyma (Fig. 1k, l) compared to air-exposed controls. We assessed the levels of TLR7 protein in the lungs in experimental COPD by immunoblot (Fig. 1m). Lung TLR7 protein levels were increased after 8 weeks of CS exposure (Fig. 1n). We also found increased TLR7 protein levels in small airway epithelial cells and parenchyma-associated inflammatory cells in CS-exposed mice (Fig. 1o, Supplementary Fig. 5). We stained the lungs of mice with experimental COPD and control for TLR7 by immunofluorescence. TLR7^+^ cells were increased after 8 weeks CS exposure (Fig.1p, Supplementary Fig. 6). We then assessed the numbers of TLR7^+^ mast cells and macrophages in the lungs in experimental COPD after 8 weeks of CS exposure. Both mMCP4^+^TLR7^+^ mast cells (Fig. 1q, Supplementary Fig. 7a) and F4/80^+^TLR7^+^ macrophages (Fig. 1r, Supplementary Fig. 7b) were significantly increased in the lungs after 8 weeks CS. We also found that TLR7 protein was mainly located in the cytoplasm, likely in epithelial and inflammatory cells (by morphology, Supplementary Fig. 6).

### CS-induced experimental COPD/emphysema is reduced in Tlr7^−/−^ mice

**Fig. 2:**
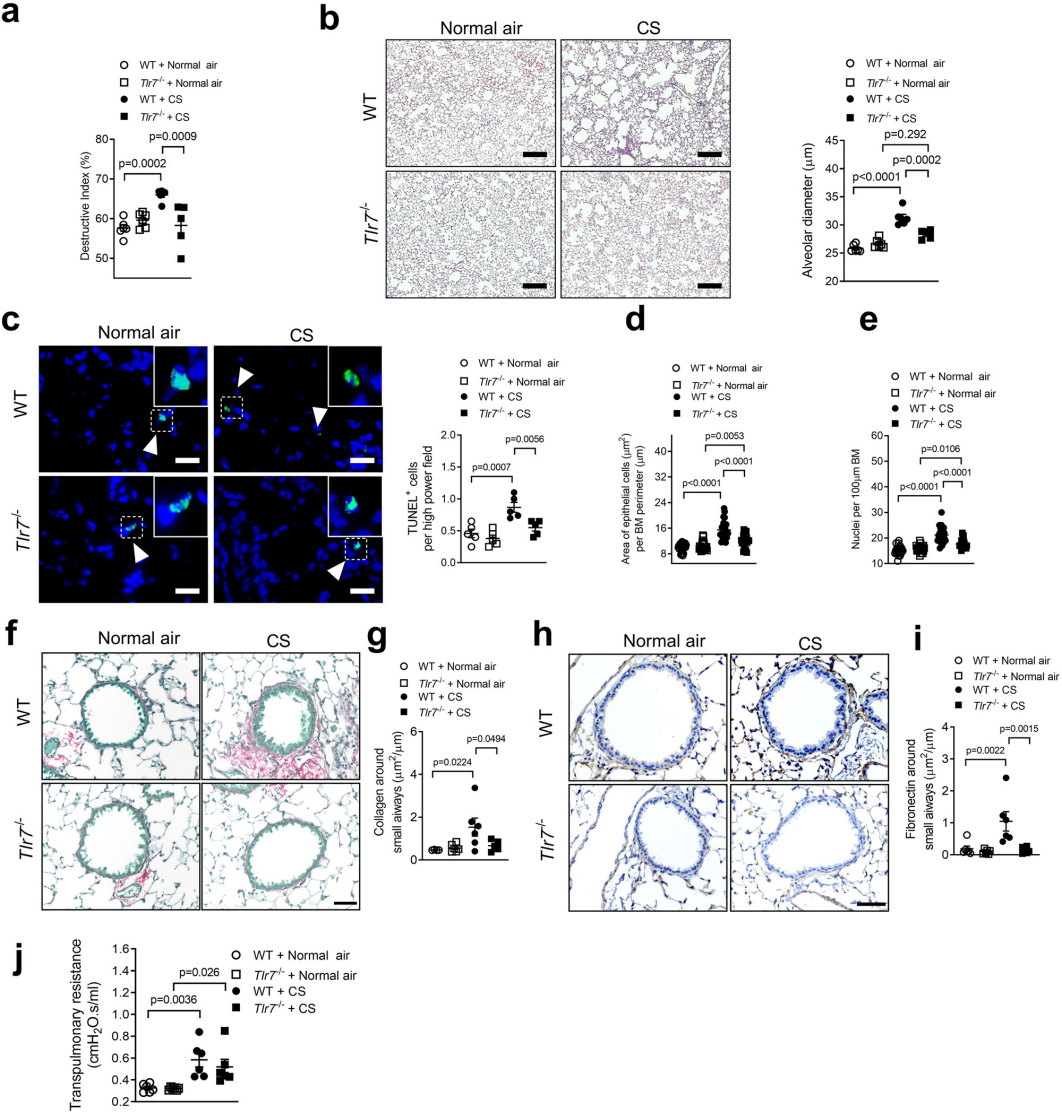
TLR7 promotes emphysema-like alveolar enlargement, airway remodeling and apoptosis in experimental COPD. Wild-type (WT) BALB/c mice and *Tlr7*^−/−^ mice (female, 6–8 weeks old) were exposed to cigarette smoke (CS) or normal air for 8 weeks. **a** Quantification of destructive index (*n* = 6 per group). **b** Representative micrographs (left) of hematoxylin and eosin-stained lung sections from WT (top panels) and *Tlr7*^−/−^ (bottom panels) mice exposed to normal air (left panels) or CS (right panels). Scale bars, 200 µm. Quantification of mean linear intercept (right, *n* = 6 per group). **c** Representative micrographs (left) of TUNEL-stained lung sections from WT (top panels) and *Tlr7*^−/−^ (bottom panels) mice exposed to normal air (left panels) or CS (right panels). Arrows indicate TUNEL^+^ cells. Scale bars, 20 µm. Quantification of apoptotic cells (right, *n* = 5 per group). **d** Quantification of small airway epithelial cell area per µm of basement membrane (BM) perimeter and **e** nuclei numbers per 100 µm of BM perimeter of normal air- or CS-exposed WT and *Tlr7*^−/−^ mice (4 small airways per mouse, *n* = 6 per group). **f** Mouse lung sections were stained with Sirius red and fast green. Scale bar=50 um. **g** Quantification of collagen around the small airways of air- or CS-exposed WT and *Tlr7*^−/−^ mice (4 small airways per mouse, *n* = 6 per group). **h** Lung sections were stained with fibronectin by immunohistochemistry. Scale bar=50 um and **i** quantification of fibronectin around the small airways of air- or CS-exposed WT and *Tlr7*^−/−^ mice (4 small airways per mouse, *n* = 6 per group). **j** Transpulmonary resistance of normal air- or CS-exposed WT and *Tlr7*^−/−^ BALB/c mice (*n* = 6 per group). All data are presented as means ± s.e.m. and are representative of two independent experiments. Statistical analysis was performed using one-way ANOVA with Bonferroni’s multiple comparison test. ns, not significant. Source data are provided as a Source Data file.

We next determined that TLR7 has a role in CS-induced emphysema-like alveolar enlargement. WT and *Tlr7*^−/−^ BALB/c mice were exposed to CS or normal air for 8 weeks^26–28,37–47^. CS-exposed WT mice had increased alveolar septal damage (Fig. 2a) and diameter (Fig. 2b) compared to air-exposed WT controls. In contrast, CS-exposed *Tlr7*^−/−^ mice had no septal damage and only marginally increased alveolar enlargement compared to air-exposed *Tlr7*^−/−^ and CS-exposed WT controls. These reductions in alveolar damage and diameter were associated with reduced terminal deoxynucleotidyl transferase dUTP nick end labeling (TUNEL)^+^ cells in the parenchyma, indicating reduced apoptosis, in CS-exposed *Tlr7*^−/−^ compared to CS-exposed WT controls (Fig. 2c). We found that TLR7^+^CD8^+^ cells were significantly increased in the lungs of WT mice after 8 weeks CS exposure, however, these cells were not detected in CS-exposed *Tlr7*^−/−^ mice (Supplementary Fig. 8).

We previously showed that mice developed small airway remodeling in experimental COPD^27,28,43^. CS exposure of WT and *Tlr7*^−/−^ mice increased small airway epithelial cell area (thickening) compared to their respective air-exposed controls (Fig. 2d, Supplementary Fig. 9a). Notably, however, CS-exposed *Tlr7*^−/−^ mice had reduced small airway epithelial cell thickening compared to CS-exposed WT controls. We then determined whether reduced epithelial cell thickening was associated with decreased numbers of nuclei, an indicator of reduced numbers of epithelial cells. CS exposure of WT and *Tlr7*^−/−^ mice increased nuclei numbers compared to their respective air-exposed controls (Fig. 2e, Supplementary Fig. 9b). However, CS-exposed *Tlr7*^−/−^ mice had reduced small airway nuclei numbers compared to CS-exposed WT controls. We also assessed the levels of collagen and fibronectin (an important component of the extracellular matrix) as additional measures of airway remodeling^48^. Collagen and fibronectin were increased around the small airways in experimental COPD that were completely inhibited in the absence of TLR7 (Fig. 2f–i).

Next, we defined the effect of TLR7 on lung function in terms of transpulmonary resistance^43^. CS exposure of WT and *Tlr7*^−/−^ mice increased transpulmonary resistance compared to air-exposed WT and *Tlr7*^−/−^ controls (Fig. 2j). Transpulmonary resistance was not different between CS-exposed WT and *Tlr7*^−/−^ mice. We also assessed the role of TLR7 in CS-induced pulmonary inflammation, mRNA levels of pro-inflammatory cytokines/chemokines, and COPD-related factors in lung homogenates and interferon-related factors (Supplementary Fig. 10). These were not different between CS-exposed WT and *Tlr7*^−/−^ mice.

We found that CS exposure reduced *Ptgds*, *C5a* and *Cxcr3* mRNA expression in WT mice, with trends to more significant reductions in *Tlr7*^−/−^ mice (Supplementary Fig. 11a–c). *Cxcl5* and *Cxcr1* mRNAs were further increased in *Tlr7*^−/−^ mice (Supplementary Fig. 11d, e). *Cxcl10* mRNA was increased in WT but not *Tlr7*^−/−^ mice after 8 weeks CS exposure (Supplementary Fig. 11f). *Cxcr2* mRNA was increased in *Tlr7*^−/−^ but not WT mice (Supplementary Fig. 11g). *Ptges2*, *Ltb4r1* and *Cxcl14* mRNAs were not changed with CS-exposure (Supplementary Fig. 11h–j). This suggests that there are global small changes in mast cell chemokines that may reduce their influx in *Tlr7*^−/−^ mice.

### Administration of TLR7 agonist imiquimod induces experimental COPD/emphysema

**Fig. 3:**
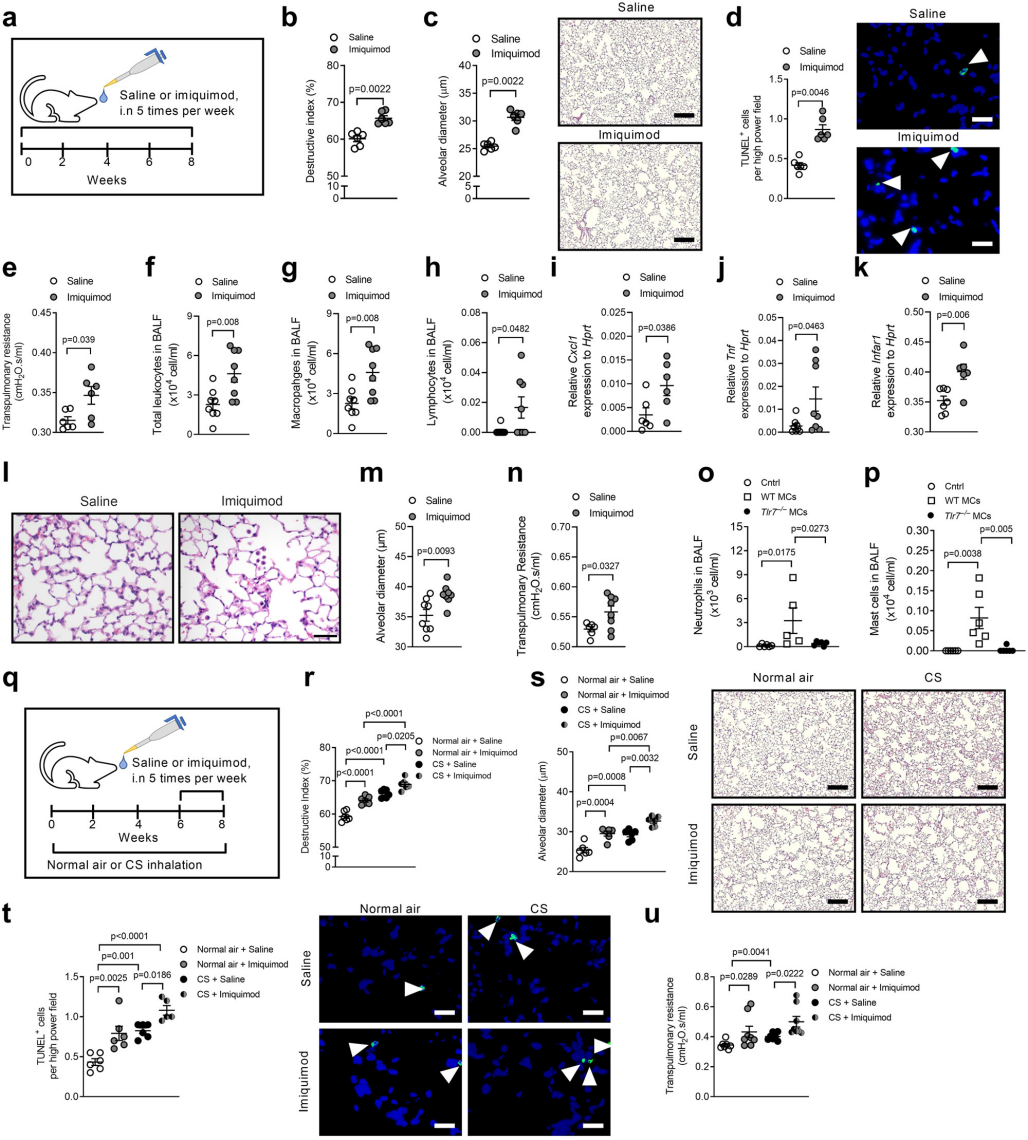
Pulmonary administration of the synthetic TLR7 agonist imiquimod induces emphysema-like alveolar enlargement and apoptosis and impairs lung function in mice. **a** Wild-type (WT) BALB/c mice (female, 6–8 weeks old) were administered low-dose imiquimod (50 μg in 50 μl sterile saline), intranasally (i.n.) 5 times per week, for 8 weeks. Controls received sterile saline. **b** Quantification of destructive index (*n* = 6 mice per group) of saline- or imiquimod-administered WT mice. **c** Quantification of mean linear intercept (*n* = 6 mice per group) and representative micrographs (right) of hematoxylin and eosin (H&E)-stained lung sections from saline (top panel)- or imiquimod (bottom panel)-administered WT mice. Scale bars, 200 µm. **d** Quantification of apoptotic cells (*n* = 6 mice per group) and representative micrographs (right) of TUNEL-stained lung sections from saline (top panel)- or imiquimod (bottom panel)-administered WT mice. Arrows indicate TUNEL^+^ cells. Scale bars, 20 µm. **e** Transpulmonary resistance of saline- or imiquimod-administered WT mice (*n* = 6 mice per group). WT BALB/c mice (female, 6–8 weeks old, *n* = 8) were challenged with high-dose imiquimod (100 μg in 50 μl sterile saline) intranasally, 5 times per week, for 2 weeks. Controls were challenged with sterile saline. **f** Total leukocytes, **g** macrophages, and **h** lymphocytes in bronchoalveolar lavage fluid (BALF, *n* = 6 mice per group). mRNA expression of **i** *Cxcl1*, **j** *Tnf*, **k** *Infar1* were assess in lungs by qPCR (*n* = 6 mice per group). **l** Lungs were stained with H&E (scale bar = 50 μm) and **m** alveolar diameter was assessed (*n* = 6 mice per group). **n** Lung function, in terms of transpulmonary resistance was assessed using the flexiVent system (*n* = 6 mice per group). WT BALB/c mice were administered 5×10^5^ bone-marrow-derived mast cells intranasally from either WT or *Tlr7*^−/−^ mice. **o** Neutrophils and **p** mast cells were counted in BALF 3 days after receiving mast cells (*n* = 5 mice per group). **q** WT BALB/c mice were exposed to normal air or CS for 8 weeks and some groups were administered imiquimod (50 μg in 50 μl sterile saline), i.n. 5 times per week, between Week 6 to 8 (for 2 weeks). Controls received sterile saline. **r** Quantification of the destructive index (*n* = 6 mice per group) of saline- or imiquimod-administered WT mice exposed to normal air or CS for 8 weeks. **s** Quantification of mean linear intercept (*n* = 6 mice per group) and representative micrographs (right) of H&E-stained lung sections from saline (top panel)- or imiquimod (bottom panel)-administered WT mice exposed to normal air (left panel) or CS (right panel) for 8 weeks. Scale bars, 200 µm. **t** Quantification of apoptotic cells (*n* = 6 mice per group) and representative micrographs (right) of TUNEL-stained lung sections from saline (top panel)- or imiquimod (bottom panel)-administered WT mice exposed to normal air (left panel) or CS (right panel) for 8 weeks. Arrows indicate TUNEL^+^ cells. Scale bars, 20 µm. **u** Transpulmonary resistance of saline- or imiquimod-administered WT mice exposed to normal air or CS for 8 weeks (*n* = 8 mice per group). All data are presented as means ± s.e.m. and are representative of two independent experiments. For panels **b**–**n** statistical analysis was performed using two-tailed Mann–Whitney test. For the rest of the panels, statistical analysis was performed using one-way ANOVA with Bonferroni’s multiple comparison test. Source data are provided as a Source Data file.

We then assessed the effects of chronic intranasal (i.n) administration of imiquimod in the absence of CS for 8 weeks (Fig. 3a). Imiquimod administration to WT mice increased alveolar septal damage and diameter compared to saline-administered WT controls (Fig. 3b, c). These effects were associated with increased TUNEL^+^ parenchyma cells (Fig. 3d). Imiquimod also increased transpulmonary resistance (Fig. 3e).

Imiquimod administration (50 μg per mouse) to WT mice did not alter inflammatory cell numbers in bronchoalveolar lavage fluid (BALF), histopathology scores, small airway epithelial cell thickening or nuclei numbers, or interferon-related mRNA levels (Supplementary Fig. 12). To assess the role of inflammation, mice were challenged with a higher dose of imiquimod (100 μg in 50 μl sterile saline, i.n., 5 times per week, for 2 weeks). This increased total leukocytes in BALF compared to vehicle-treated controls (Fig. 3f). Differential cell counts showed that macrophages were the predominant cell type and that macrophages (Fig. 3g) and lymphocytes (Fig. 3h) were increased with imiquimod. To further assess inflammation, we extracted RNA from the lungs, and measured inflammatory markers by qPCR. *Cxcl1*, *Tnf*, IFN-γ (*Ifng*) and IFN receptor-α1 (*Ifnar1*) mRNA levels were increased in mice challenged with imiquimod compared to controls (Fig. 3i–k). There were no differences in *Il6*, IFN-λ (*Infl*), -γ (*Infg*), -β (*Infb*) or -α1(*Infa1*) expression (Supplementary Fig. 13). High dose imiquimod challenge increased alveolar diameter (Fig. 3I, m) and impaired lung function with increased transpulmonary resistance (Fig. 3n). The mRNA expression of the TLR7 regulated genes *Ifr1*, *Stat1*, *Myd88*, *Cxcl10* and *Oas1a* were significantly increased in mouse lungs after high dose imiquimod challenge (Supplementary Fig. 14), but *Il6* mRNA expression was not changed (Supplementary Fig. 13b).

### Bone marrow-derived *Tlr7*^−/−^ mast cells induce less airway inflammation than WT mast cells

Mast cells were cultured from the bone marrow of WT or *Tlr7*^−/−^ mice and 5×10^5^ cells transferred intranasally into WT mice. Transfer of either cell population increased total leukocyte and macrophage numbers in the airways after 1 and 3 days. WT mast cells also increased the numbers of neutrophils and mast cells over 1–3 days but these were only transiently increased after 1 day and were back to baseline after 3 days with *Tlr7*^−/−^ mast cells (Fig. 3o, p, Supplementary Fig. 15a-f).

### Imiquimod administration increases severity of experimental COPD/emphysema

We next determined if exogenous imiquimod affects CS-induced experimental COPD. WT mice were exposed to CS or normal air for 8 weeks and administered sterile saline or imiquimod i.n between weeks 6–8 (Fig. 3q). Imiquimod administration to CS-exposed mice further increased alveolar septal damage (Fig. 3r) and diameter (Fig. 3s) compared to imiquimod-administered air-exposed and, saline-administered CS-exposed controls. Notably, imiquimod-administered air-exposed mice also had increased alveolar septal damage and diameter compared to saline-administered air-exposed controls. Increased alveolar septal damage and diameter in imiquimod-administered CS- and air-exposed mice were associated with increased TUNEL^+^ parenchyma cells (Fig. 3t). Then, we assessed the effects of imiquimod on lung function. Saline-administered CS-exposed mice had increased transpulmonary resistance compared to saline-administered air-exposed controls (Fig. 3u). In contrast, resistance was not altered in imiquimod-administered CS-exposed mice compared to imiquimod-administered air-exposed controls. This was because increased resistance occurred in imiquimod-administered compared to saline-administered air-exposed controls. Imiquimod-administered CS-exposed mice had increased resistance compared to saline-administered CS-exposed controls.

Imiquimod did not alter the numbers of inflammatory cells in BALF, histopathology score, small airway epithelial cell thickening, or nuclei numbers and had minimal effects on interferon-related mRNA levels (Supplementary Fig. 16a–p).

### Imiquimod-induced emphysema is TLR7- and MyD88-dependent

**Fig. 4:**
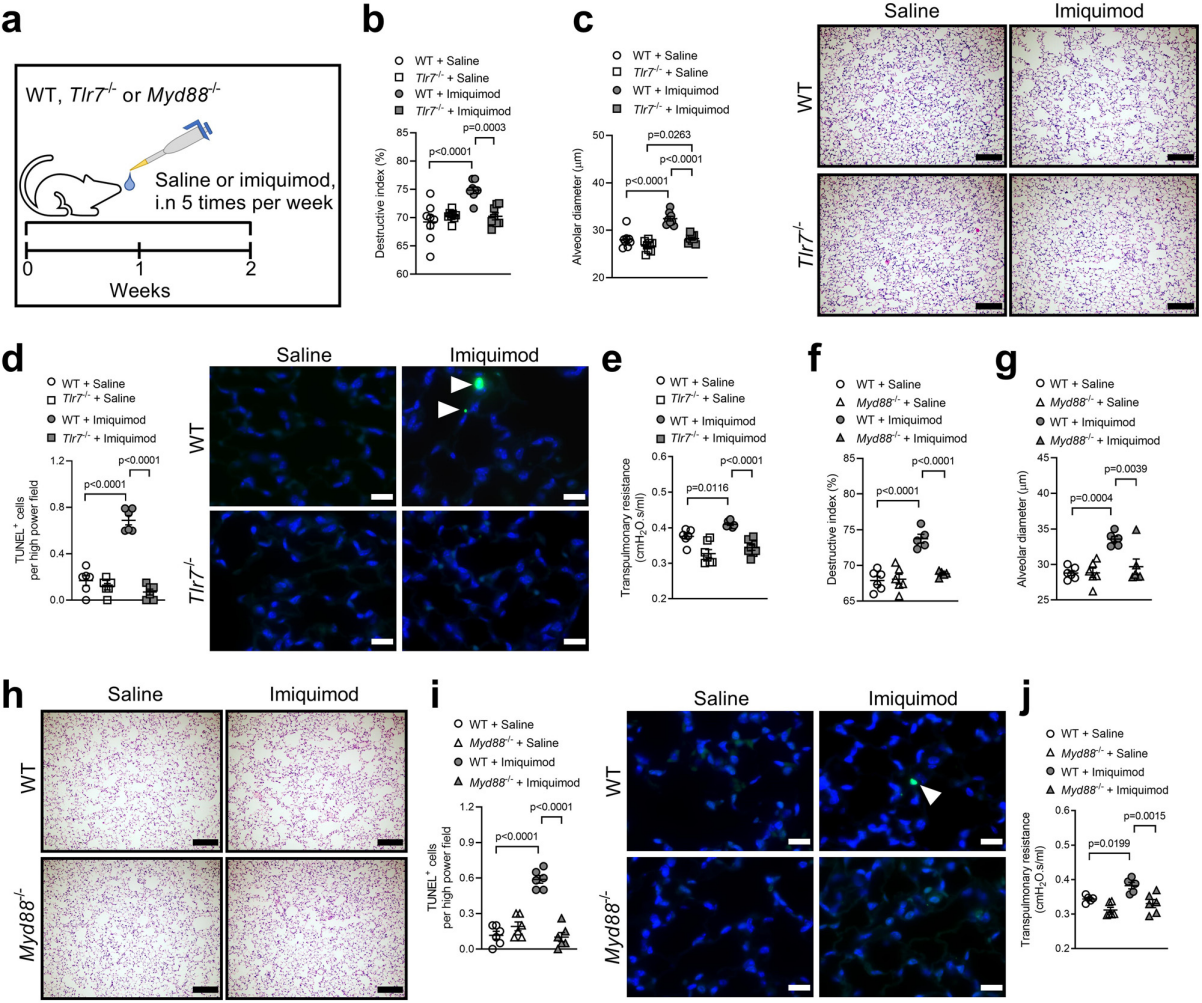
Imiquimod induces emphysema in a TLR7- and MyD88-dependent manner. **a** Wild-type (WT) or TLR7-deficient (*Tlr7*^−/−^) or MyD88-deficient (*Myd88*^−/−^) BALB/c mice (female, 6–8 weeks old) were administered imiquimod (50 μg in 50 μl sterile saline), intranasally (i.n.) 5 times per week, for 2 weeks. Controls received sterile saline. **b** Quantification of destructive index (*n* = 8 mice per group) of saline- or imiquimod-administered WT and *Tlr7*^−/−^ mice. **c** Quantification of mean linear intercept (*n* = 8 mice per group) and representative micrographs (right) of hematoxylin and eosin (H&E)-stained lung sections from WT (top panels) and *Tlr7*^−/−^ (bottom panels) mice administered saline (left panels) or imiquimod (right panels). Scale bars, 200 µm. **d** Quantification of apoptotic cells (*n* = 6 mice per group) and representative micrographs (right) of TUNEL-stained lung sections from WT (top panels) and *Tlr7*^−/−^ (bottom panels) mice administered saline (left panels) or imiquimod (right panels). Arrows indicate TUNEL^+^ cells. Scale bars, 20 µm. **e** Transpulmonary resistance of saline- or imiquimod-administered WT and *Tlr7*^−/−^ mice (*n* = 8 mice per group). **f** Quantification of destructive index (*n* = 6 mice per group) of saline- or imiquimod-administered WT and *Myd88*^−/−^ mice. **g** Quantification of mean linear intercept (*n* = 6 mice per group) and **h** representative micrographs of H&E-stained lung sections from WT (top panels) and *Myd88*^−/−^ (bottom panels) mice administered saline (left panels) or imiquimod (right panels). Scale bars, 200 µm. **i** Quantification of apoptotic cells (*n* = 6 mice per group) and representative micrographs (right) of TUNEL-stained lung sections from WT (top panels) and *Myd88*^−/−^ (bottom panels) mice administered saline (left panels) or imiquimod (right panels). Arrows indicate TUNEL^+^ cells. Scale bars, 20 µm. **j** Transpulmonary resistance of saline- or imiquimod-administered WT and *Myd88*^−/−^ mice (*n* = 6 mice per group). All data are presented as means ± s.e.m. Statistical analysis was performed using one-way ANOVA with Bonferroni’s multiple comparison test. Source data are provided as a Source Data file.

Next, we determined if imiquimod-induced emphysema has a short-term effect and is TLR7- and MyD88-dependent. We administered saline or imiquimod acutely i.n to WT, *Tlr7*^−/−^ or *Myd88*^−/−^ mice for 2 weeks (Fig. 4a). Short-term imiquimod increased alveolar septal damage (Fig. 4b) and diameter (Fig. 4c) in WT mice compared to saline-administered controls. These effects were significantly reduced in imiquimod-administered *Tlr7*^−/−^ compared to WT controls. This was associated with reduced TUNEL^+^ parenchyma cells (Fig. 4d) and reduced transpulmonary resistance (Fig. 4e).

Notably, imiquimod-induced alveolar septal damage (Fig. 4f) and diameter (Fig. 4g, h) were significantly reduced in *Myd88*^−/−^ compared to imiquimod-administered WT controls. These were also associated with reduced TUNEL^+^ parenchyma cells (Fig. 4i) and transpulmonary resistance (Fig. 4j).

Acute imiquimod administration did not alter the numbers of inflammatory cells in BALF, histopathology scores, small airway epithelial cell thickening or nuclei numbers or interferon-related mRNA levels in *Tlr7*^−/−^ (Supplementary Fig. 17a–o) or *Myd88*^−/−^ mice (Supplementary Fig. 18a–o).

### Imiquimod promotes pulmonary mast cell influx

**Fig. 5:**
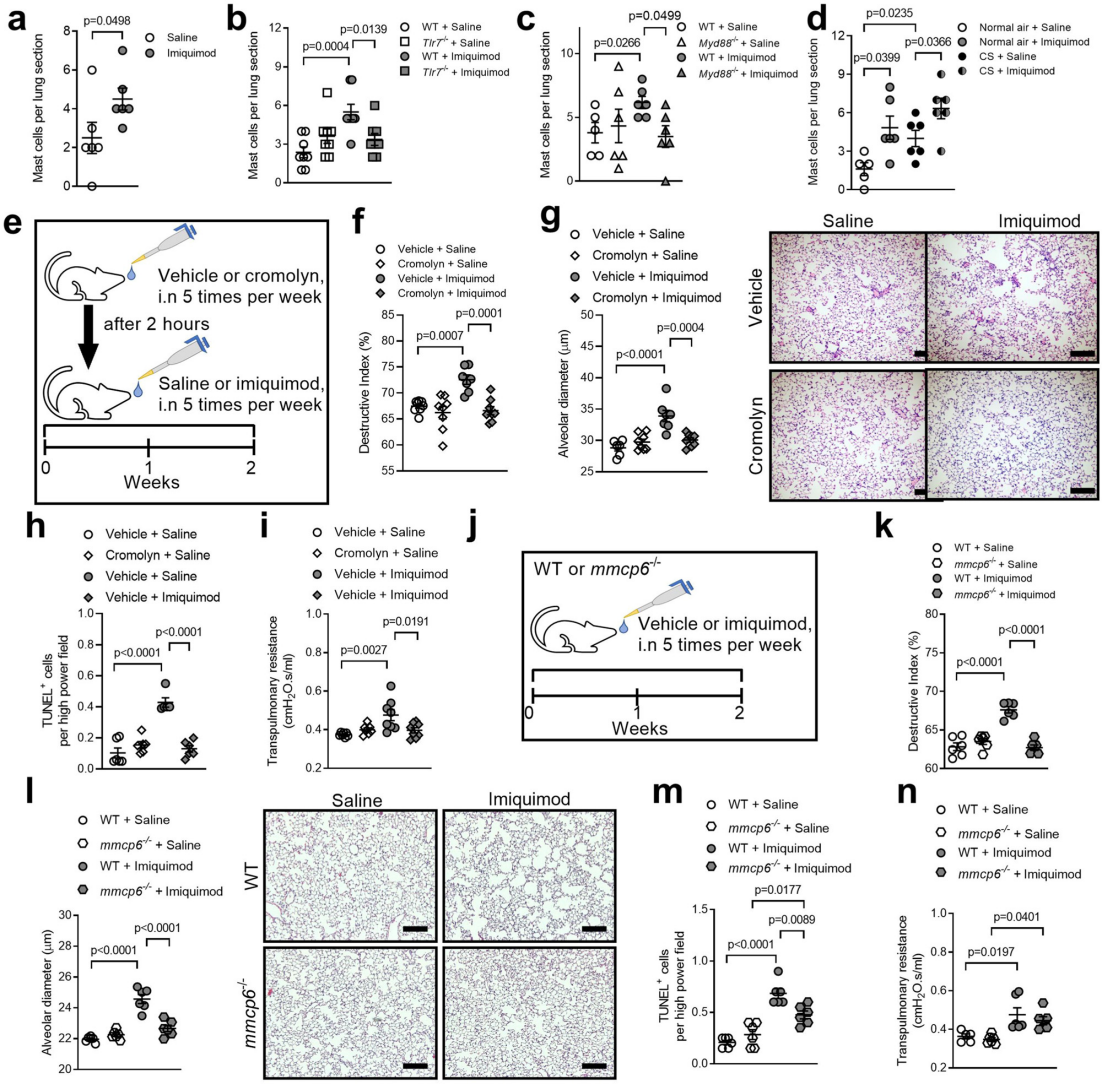
Imiquimod induces pulmonary mast cell influx and imiquimod-induced emphysema is reduced in mice treated with the mast cell stabilizer cromolyn or deficient in the mast cell tryptase mMCP6. **a** Quantification of mast cells in lung sections from wild type (WT) BALB/c mice (female, 6–8 weeks old, *n* = 6 mice per group) administered imiquimod or vehicle for 8 weeks. Quantification of mast cells in lung sections from **b** WT, TLR7- (*Tlr7*^−/−^) or **c** MyD88-deficient (*Myd88*^−/−^) BALB/c mice (female, 6–8 weeks old) administered imiquimod or vehicle for 2 weeks (*n* = 8 mice per group). **d** Quantification of mast cells in lung sections from WT BALB/c mice exposed to normal air or CS for 8 weeks and administered imiquimod or vehicle from weeks 6–8 (*n* = 6 mice per group). **e** WT mice were first administered cromolyn (50 mg/kg body weight) or vehicle (sterile water), and after 2 h, were administered imiquimod (50 μg) or vehicle. Cromolyn, imiquimod, and vehicle were delivered intranasally (i.n.) 5 times per week, for 2 weeks. **f** Quantification of the destructive index (*n* = 8 mice per group) of vehicle- or imiquimod-administered mice with or without cromolyn treatment. **g** Quantification of mean linear intercept (*n* = 8 mice per group) and representative micrographs (right) of hematoxylin and eosin (H&E)-stained lung sections from vehicle (top panels) and cromolyn (bottom panels) mice administered vehicle (left panels) or imiquimod (right panels). Scale bars, 200 µm. **h** Quantification of apoptotic cells (*n* = 6 mice per group). **i** Transpulmonary resistance of saline- or imiquimod-administered mice with or without cromolyn treatment (*n* = 8 mice per group). **j** WT or mouse mast cell protease-6-deficient (*mmcp6*^−/−^) C57BL/6 mice were administered imiquimod (50 μg in 50 μl sterile saline), intranasally 5 times per week, for 2 weeks. Controls received sterile saline. **k** Quantification of destructive index (*n* = 6 mice per group) of saline- or imiquimod-administered WT and *mmcp6*^−/−^ mice. **l** Quantification of mean linear intercept (*n* = 6 mice per group) and representative micrographs (right) of H&E-stained lung sections from WT (top panels) and *mmcp6*^−/−^ (bottom panels) mice administered saline (left panels) or imiquimod (right panels). Scale bars, 200 µm. **m** Quantification of apoptotic cells (*n* = 6 mice per group). **n** Transpulmonary resistance of saline- or imiquimod-administered WT and *mmcp6*^−/−^ mice (*n* = 6 mice per group). All data are presented as means ± s.e.m. For panel **a**, statistical differences were determined by two-tailed Mann–Whitney test. For rest of panels, statistical analysis was performed using one-way ANOVA with Bonferroni’s multiple comparison test. Source data are provided as a Source Data file.

We and others showed that mast cells are involved in human and experimental COPD^21–23,27,28^. Given that certain population of mast cells express TLR7^24–26^, we next assessed the numbers of pulmonary mast cells in mice administered imiquimod for 8 weeks (Fig. 3a) or during weeks 6–8 of CS exposure (Fig. 3q). Chronic imiquimod administration increased pulmonary mast cell numbers compared to saline-administered controls (Fig. 5a). Imiquimod-induced pulmonary mast cell influx was TLR7- (Fig. 5b) and MyD88-dependent (Fig. 5c). Consistent with our previous studies^42^, pulmonary mast cell numbers were increased in saline-administered CS-exposed compared to air-exposed controls (Fig. 5d). Imiquimod increased pulmonary mast cells in CS- and air-exposed mice compared to respective saline-administered CS- and air-exposed controls.

### Imiquimod-induced emphysema is ablated by cromolyn administration and in *mmcp6*^−/−^ mice

Next, we assessed the impact of cromolyn on imiquimod-induced emphysema. Cromolyn stabilizes and prevents mast cell degranulation and the release of their contents including tryptases^49^. WT mice were administered cromolyn followed by imiquimod 2 h later i.n for 2 weeks (Fig. 5e). Cromolyn had no effect on saline-administered control mice. Imiquimod administration increased alveolar septal damage (Fig. 5f) and diameter (Fig. 5g), TUNEL^+^ parenchyma cells (Fig. 5h, Supplementary Fig. 19) and transpulmonary resistance (Fig. 5i) compared to vehicle-administered controls. Each of these imiquimod-induced effects were ablated in cromolyn-treated mice compared to vehicle-administered controls. Cromolyn or imiquimod did not alter inflammatory cell numbers in BALF, histopathology scores, small airway epithelial cell thickening or nuclei numbers (Supplementary Fig. 20a–k).

We previously demonstrated that the mast cell-specific tryptases, mMCP6 and protease serine member S (Prss)31, play critical roles in experimental CS-induced emphysema^27,28^. mMCP6 proteins were increased in mouse lungs after 8 weeks CS exposure (Supplementary Fig. 21). To assess the relationship between TLR7 and mast cell granule-specific tryptase, WT or *mmcp6*^−/−^ C57BL/6 mice were administered imiquimod i.n for 2 weeks (Fig. 5j). Imiquimod administration to WT mice increased alveolar septal damage (Fig. 5k) and diameter (Fig. 5l) compared to saline-administered WT controls. In contrast, administration to *mmcp6*^−/−^ mice did not increase alveolar septal damage or diameter compared to saline-administered *mmcp6*^−/−^ controls. Increased alveolar septal damage and diameter were also inhibited compared to imiquimod-administered WT controls. These effects were associated with reduced TUNEL^+^ parenchyma cells in imiquimod-administered *mmcp6*^−/−^ mice compared to imiquimod-administered WT controls (Fig. 5m, Supplementary Fig. 22). Imiquimod administration to WT or *mmcp6*^−/−^ mice also increased transpulmonary resistance compared to their respective saline-administered controls (Fig. 5n). However, resistance was not different between imiquimod-administered WT and *mmcp6*^−/−^ mice. Imiquimod administration to WT or *mmcp6*^−/−^ mice did not alter BALF inflammatory cell numbers, histopathology score, airway epithelial cell thickening or nuclei numbers (Supplementary Fig. 23a–k). To test if imiquimod-induced emphysema was dependent on secreted mast cell tryptase, we administered imiquimod to *Prss31*^−/−^ mice that lack mast cell surface tryptase. Imiquimod-induced emphysema was similar in *Prss31*^−/−^ mice and WT controls (Supplementary Fig. 24a and b).

### Human tryptase-β challenge induces inflammation and experimental COPD

**Fig. 6:**
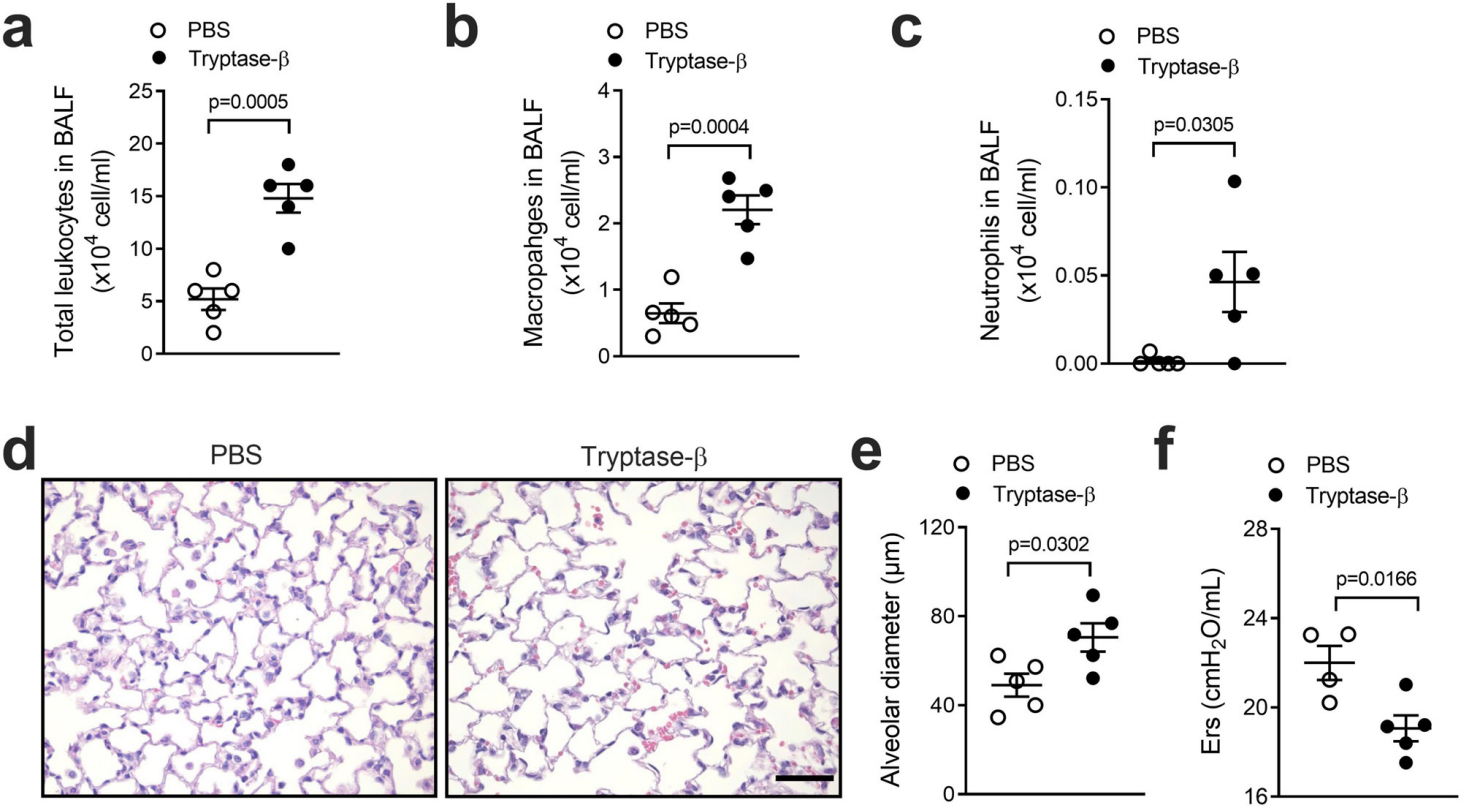
Challenge with human tryptase-β induces inflammation and experimental COPD. Mice (6–8 weeks old, female) were challenged with recombinant human tryptase-β (20 μg in 50 μL PBS/mouse) intranasally for 7 days, control mice received equal volumes of PBS. Tryptase-β challenge increased the influx of **a** total leukocytes, **b** macrophages and **c** neutrophils in the airways (bronchoalveolar lavage fluid, BALF, *n* = 5 per group). **d** Lung sections were stained with hematoxylin and eosin (H&E, scale bar = 50 um), and **e** alveolar diameter was assessed using the mean linear intercept as a measure of emphysema. **f** Lung function, in terms of elastance (Ers, *n* = 5 mice per group). Results are mean ± s.e.m. Statistical analysis was performed using the two-tailed Mann–Whitney test. Source data are provided as a Source Data file.

Recombinant human tryptase-β challenge of the lungs of naïve mice increased the influx of total leukocytes (Fig. 6a), comprised predominantly of macrophages (Fig. 6b) and neutrophils (Fig. 6c), into the airways compared to vehicle challenge. Lung sections were stained with H&E for histology analysis (Fig. 6d). Tryptase-β recombinant challenge increased alveolar diameter and emphysema (Fig. 6e). Challenge also impaired lung function by reducing elastance (Ers, Fig. 6f).

### Mast cell inhibitor treatment completely inhibited the development of experimental COPD and increases in the levels of mast cell tryptase

**Fig. 7:**
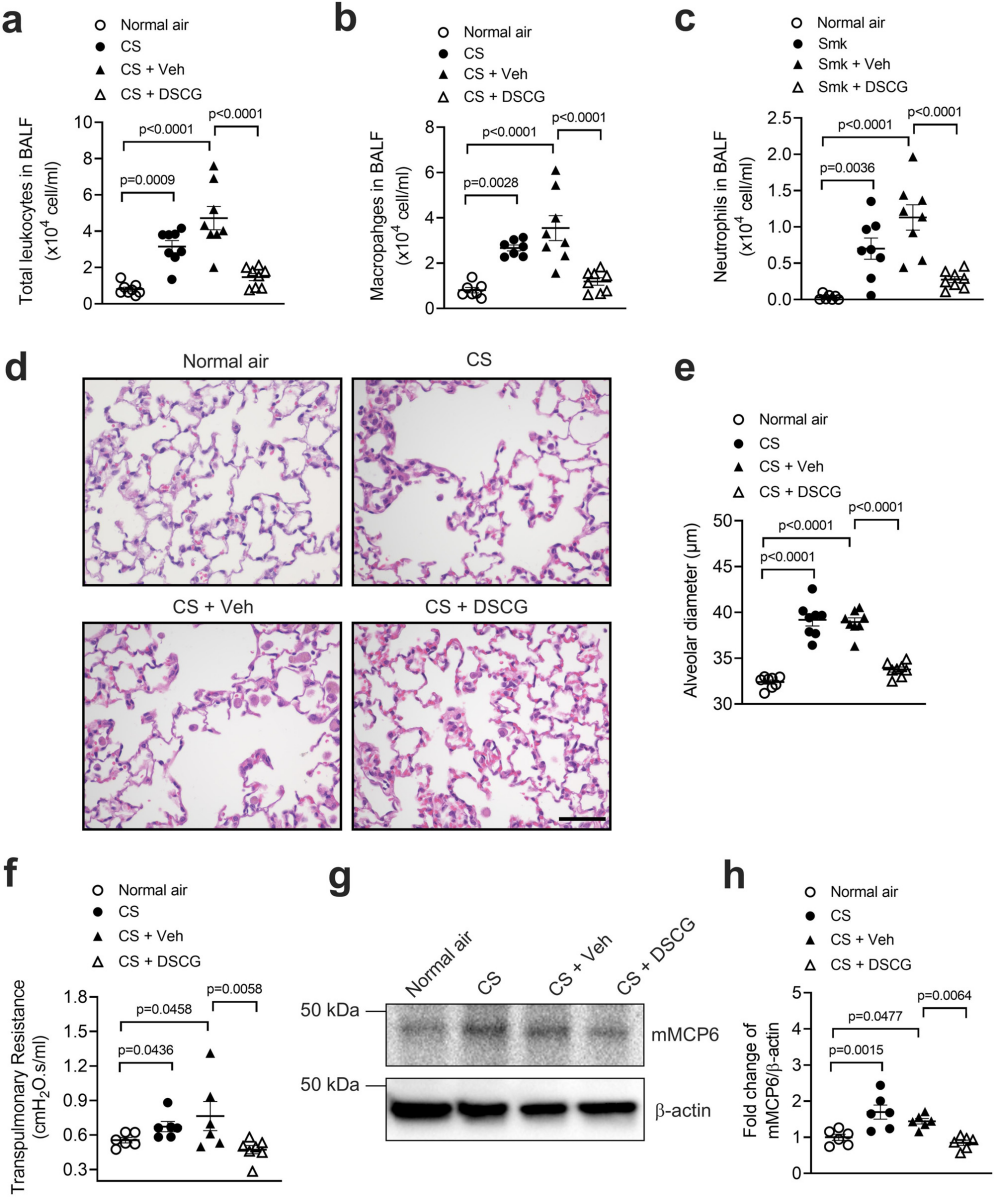
Treatment with a mast cell degranulation stabilizer completely inhibits the development of experimental COPD and increases in the levels of mast cell tryptase. Wild-type BALB/c mice (6–8 weeks old, female) were exposed to cigarette smoke (CS) for 8 weeks and treated with disodium cromoglycate (DSCG) intranasally from weeks 6–8. control mice received equal volumes of vehicle. DSCG completely inhibited the development of chronic airway inflammation, emphysema and impaired lung function in CS exposed mice. **a** Total leukocyte, **b** macrophage and **c** neutrophil numbers in bronchoalveolar lavage fluid (BALF) after 8 weeks of CS exposure (*n* = 8 per group). **d** Lung sections were stained with hematoxylin and eosin (scale bar = 50 μm), and **e** alveolar diameter was assessed as a measure of emphysema (*n* = 8 per group). **f** Lung function, in terms of transpulmonary resistance (*n* = 8 per group). **g** mMCP6 protein was assessed in whole mouse lung tissues by immunoblot (*n* = 8 per group), and **h** fold change of densitometry analysis of mMCP6 protein was normalized to β-actin. Results are mean ± s.e.m, Statistical differences were determined using one-way ANOVA followed by Bonferroni post-test. Source data are provided as a Source Data file.

To further clarify the role of the TLR7-mast cell axis in COPD we used treatment with the anti-asthma and mastocytosis drug disodium cromoglycate (DSCG, cromolyn) that inhibits the release of mediators from mast cells^50^. We administered DSCG during weeks 6–8 of CS exposure, when the chronic features emerge to induce experimental COPD. DSCG treatment completely inhibited chronic airway inflammation and the influx of total leukocytes, predominantly macrophages and neutrophils into the airways after 8 weeks CS exposure (Fig. 7a-c). Treatment also completely inhibited the development of emphysema with alveolar diameter back to baseline (Fig. 7d, e). These collective effects prevented the impairment of lung function and transpulmonary resistance (Fig. 7f). We then related our observations to the levels of mMCP6 (murine equivalent of human tryptase-β)^27^. mMCP6 levels increased in the lungs in experimental COPD, which was inhibited back to baseline with DSCG treatment (Fig. 7g, h).

### Imiquimod induces the release of mast cell tryptase from human mast cells

**Fig. 8:**
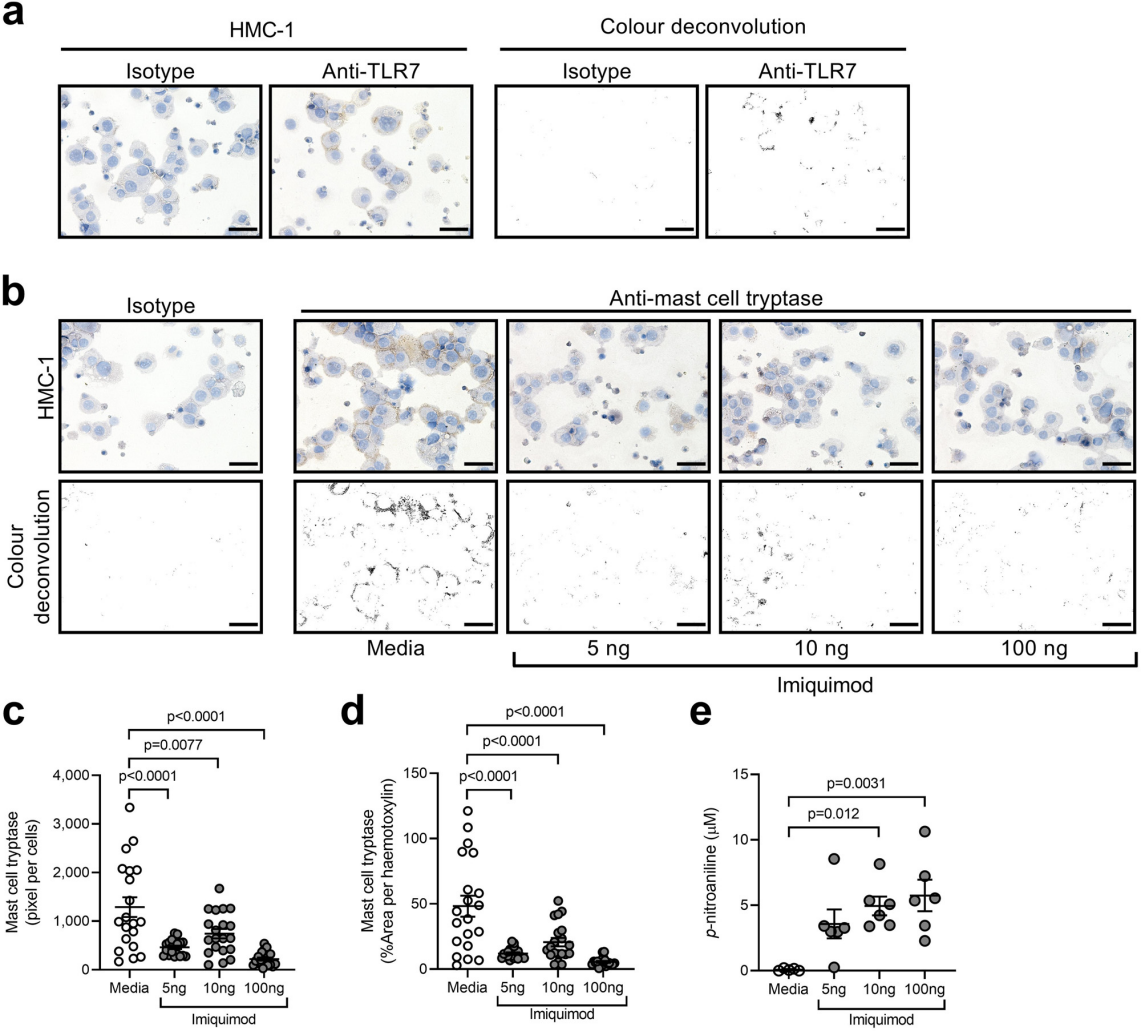
Imiquimod induces the release of mast cell tryptase from human mast cells. **a** Representative micrographs (top panels, *n* = 3) and color deconvolution of isotype control (left panels) and TLR7 (right panels) immunostaining of HMC-1 human mast cells. Scale bars, 50 µm. **b** Representative micrographs (top panels, *n* = 3) and color deconvolution (bottom panels) of isotype control (left panel) and mast cell tryptase (right panels) immunostaining of HMC-1 cells incubated with media or imiquimod (5, 10 or 100 ng) for 1 h, Scale bars, 50 µm. Quantification of mast cell tryptase in cells (10 random fields per sample, *n* = 3 per group) normalized to **c** number of cells or **d** area of hematoxylin of HMC-1 cells incubated with media or imiquimod (5, 10 or 100 ng) for 1 h. **e** Quantification of mast cell tryptase activity (*n* = 6 per group) in terms of *p*-nitroaniline levels in culture supernatants from HMC-1 cells incubated with media or imiquimod (5, 10, or 100 ng) for 1 h. Throughout, data are presented as means ± s.e.m. Statistical analysis was performed using one-way ANOVA with Bonferroni’s multiple comparison test. Source data are provided as a Source Data file.

We next determined whether imiquimod-induced tryptase release from human mast cells. First, we confirmed that human mast cell line 1 (HMC-1) cells expressed TLR7 by immunostaining (Fig. 8a). We then incubated HMC-1 cells with media or imiquimod (5, 10 or 100 ng) for 1 h. HMC-1 cells incubated with media clearly expressed mast cell tryptase (Fig. 8b). The intensity of mast cell tryptase immunostaining reduced in HMC-1 cells incubated with increasing concentrations of imiquimod. This was confirmed when mast cell tryptase was quantified in terms of DAB signal (pixels) normalized to cell numbers (Fig. 8c) or area of hematoxylin-stained cells (Fig. 8d). Furthermore, mast cell tryptase activity dose-dependently increased in culture supernatants of HMC-1 cells incubated with imiquimod compared to media (Fig. 8e). Interestingly, other proteases such as neutrophil elastase, myeloperoxidase and total matrix metalloproteinase activities were not increased in the lungs of mice chronically administered imiquimod (Supplementary Fig. 25a–c).

### Prophylactic TLR7 neutralization prevents CS-induced experimental COPD/emphysema

**Fig. 9:**
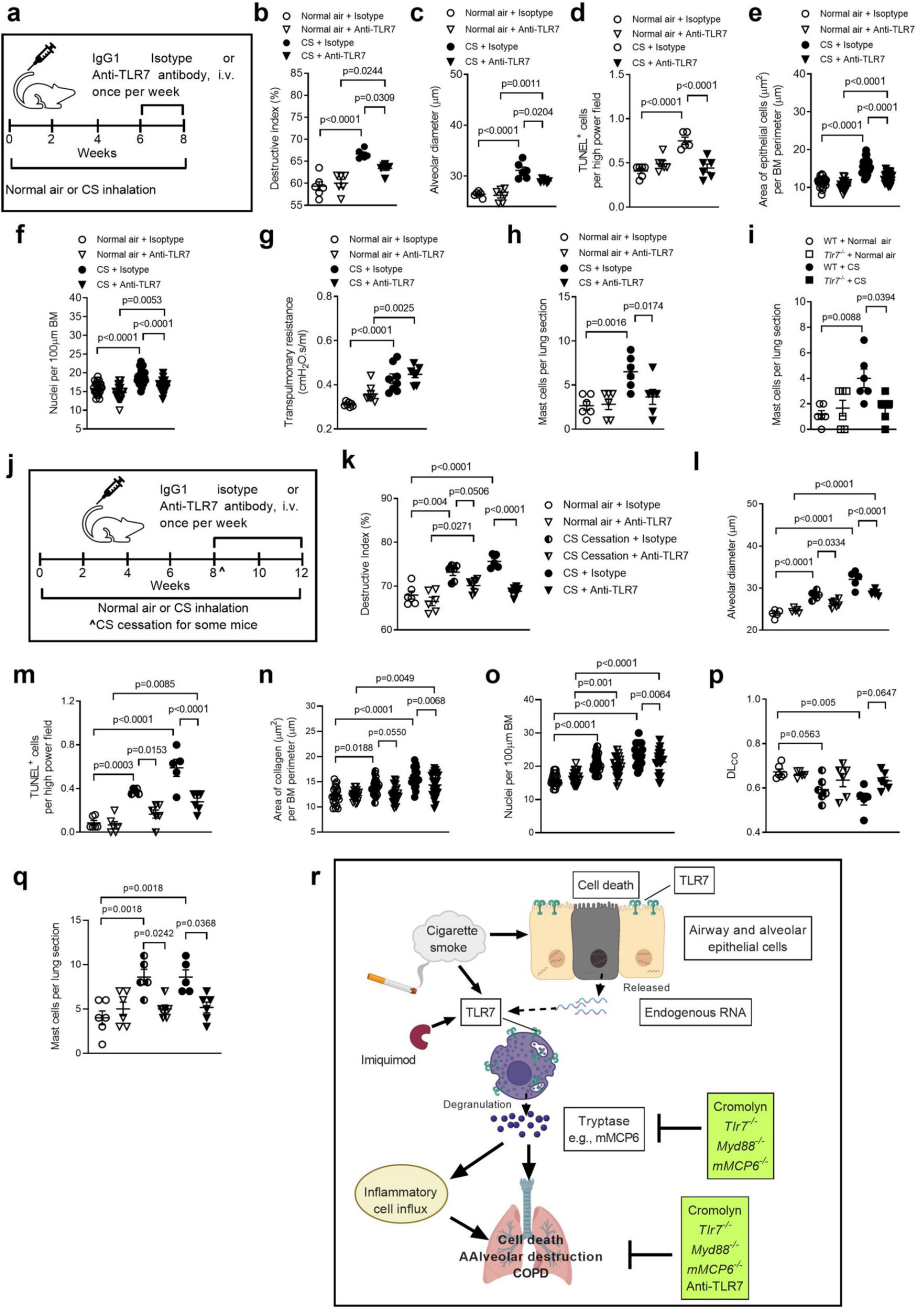
Therapeutic treatment with anti-TLR7 monoclonal antibody reduces CS-induced emphysema and mast cell influx in experimental COPD. **a** Wild-type (WT) BALB/c mice (female, 6–8 weeks old) were exposed to normal air or CS for 8 weeks and treated with neutralizing anti-TLR7 monoclonal antibody or isotype control, intravenously (i.v.) once per week for 2 weeks, from weeks 6–8. **b** Quantification of destructive index (*n* = 6 mice per group) in lungs of isotype- or anti-TLR7-treated WT mice exposed to normal air or CS for 8 weeks. **c** Quantification of mean linear intercept (*n* = 6 mice per group) of isotype or anti-TLR7 -treated WT mice exposed to normal air or CS for 8 weeks. **d** Quantification of apoptotic cells (*n* = 6 mice per group) in TUNEL-stained lung sections from isotype or anti-TLR7 treated WT mice exposed to normal air or CS for 8 weeks. Quantification of **e** small airway epithelial cell area per µm of basement membrane (BM) perimeter and **f** nuclei numbers per 100 µm of BM perimeter of isotype- or anti-TLR7-treated WT mice exposed to normal air or CS for 8 weeks (4 small airways per mouse, *n* = 6 mice per group). **g** Transpulmonary resistance of isotype- or anti-TLR7-treated WT mice exposed to normal air or CS for 8 weeks (*n* = 6 mice per group). **h** Quantification of mast cells in lung sections from isotype- or anti-TLR7-treated WT mice exposed to normal air or CS for 8 weeks (*n* = 6 mice per group). **i** Quantification of mast cells in lung sections from WT and *Tlr7* ^−/−^ mice exposed to normal air or CS for 8 weeks (*n* = 6 mice per group). **j** WT BALB/c mice (female, 6–8 weeks old) were exposed to normal air or CS for 12 weeks and treated with neutralizing anti-TLR7 monoclonal antibody or isotype control, i.v. once per week, from weeks 8–12 (for 4 weeks). Some mice had CS cessation others continued CS exposure after 8 weeks prior to anti-TLR7 treatment. **k** Quantification of destructive index, **l** mean linear intercept and **m** apoptotic cells (*n* = 6 mice per group) in lungs of isotype- or anti-TLR7-treated WT mice exposed to normal air or CS with CS cessation or continued CS exposure from 8–12 weeks. Quantification of **n** small airway epithelial cell area per µm of basement membrane (BM) perimeter and **o** nuclei numbers per 100 µm of BM perimeter of isotype- or anti-TLR7-treated WT mice exposed to normal air or CS with CS cessation or continued CS exposure from 8–12 weeks (4 small airways per mouse, *n* = 6 mice per group). **p** Measurement of diffusing lung capacity for carbon monoxide (DL_CO_) of isotype- or anti-TLR7-treated WT mice exposed to normal air or CS with CS cessation or continued CS exposure from 8–12 weeks (*n* = 6 mice per group). **q** Quantification of mast cells in lung sections from isotype- or anti-TLR7-treated WT mice exposed to normal air or CS with CS cessation or continued CS exposure from 8–12 weeks (*n* = 6 mice per group). **r** Schematic representation of proposed mechanisms of how TLR7 contributes to CS-induced apoptosis and emphysema-like alveolar enlargement in experimental COPD in a mast cell-specific tryptase-dependent manner. All data are presented as means ± s.e.m. Statistical analysis was performed using one-way ANOVA with Bonferroni’s multiple comparison test. Source data are provided as a Source Data file.

We previously showed that emphysema-like alveolar enlargement develops between weeks 6–8 of CS exposure in mice^27^. To assess the therapeutic potential of targeting TLR7, WT mice were exposed to CS or normal air for 8 weeks and treated with neutralizing anti-TLR7 monoclonal antibody or isotype control intravenously (i.v) between weeks 6–8 (Fig. 9a). Anti-TLR7-treated CS-exposed mice had increased alveolar septal damage (Fig. 9b) and diameter (Fig. 9c and Supplementary Fig. 26a) compared to air-exposed controls. However, importantly, alveolar damage and diameter were significantly reduced compared to isotype-treated CS-exposed controls. The reductions in CS-induced alveolar septal damage and diameter in anti-TLR7-treated mice were associated with reduced numbers of TUNEL^+^ parenchyma cells, which were reduced to the baseline levels in isotype- or anti-TLR7 treated air-exposed controls (Fig. 9d, Supplementary Fig. 26b).

Small airway epithelial thickening (Fig. 9e, Supplementary Fig. 26c) and nuclei numbers (Fig. 9f, Supplementary Fig. 26d) were increased in CS-exposed mice administered either isotype or anti-TLR7 antibodies compared to their air-exposed controls. Notably, anti-TLR7 treatment reduced small airway epithelial cell thickening in CS-exposed compared to isotype-treated controls. Isotype- and anti-TLR7-treated CS-exposed mice had increased transpulmonary resistance compared to isotype- and anti-TLR7-treated air-exposed controls, respectively (Fig. 9g). Resistance was similar in anti-TLR7-treated and isotype-treated CS-exposed mice.

Isotype-treated CS-exposed mice also had increased mast cell numbers compared to isotype-treated air-exposed controls (Fig. 9h). Notably, anti-TLR7 treatment of CS-exposed mice prevented the increase in lung mast cell numbers compared to isotype-treated CS-exposed controls. Numbers were reduced to baseline levels in anti-TLR7-treated air-exposed controls. Consistent with this, CS-exposed *Tlr7*^−/−^ mice did not have increased lung mast cell numbers compared to air-exposed *Tlr7*^−/−^ controls, which were also reduced to baseline in air-exposed WT controls (Fig. 9i).

Notably, anti-TLR7-treatment reduced CS-induced BALF total leukocytes compared to isotype-treated controls (Supplementary Fig. 27a). This was due to the suppression of macrophage but not neutrophil or lymphocyte numbers (Supplementary Fig. 27b–d). Anti-TLR7 treatment did not alter histopathology score in CS-exposed compared to isotype-treated controls (Supplementary Fig. 27e–i). Treatment partially restored *Ifna* mRNA expression in CS-exposed mice (Supplementary Fig. 27j) but had no effect on *Ifnb*, *Ifng*, *Ifnl* or *Ifnar1* mRNA levels (Supplementary Fig. 27k-n).

### Therapeutic anti-TLR7 treatment suppresses CS-induced experimental COPD/emphysema

We then assessed therapeutic treatment with anti-TLR7 monoclonal antibody to reduce experimental COPD progression and severity. WT mice were exposed to CS for 8 weeks until the disease developed. Controls were exposed to normal air. Some mice continued to be CS-exposed and were treated with a neutralizing anti-TLR7 monoclonal antibody or isotype control i.v. between weeks 8–12 to assess the effects of treatment on disease progression (Fig. 9j). Some mice underwent CS cessation after 8 weeks of CS exposure prior to assessing the effects of treatment from weeks 8–12 in reversing disease. Isotype and anti-TLR7 treatments had no effect on air-exposed mice. Isotype-treated continually CS-exposed and CS cessation mice had increased alveolar septal damage (Fig. 9k) and diameter (Fig. 9l, Supplementary Fig. 28a) compared to isotype-treated air-exposed controls. Anti-TLR7-treated continually CS-exposed groups had a slight increase in alveolar septal damage and diameter compared to anti-TLR7-treated air-exposed controls. Anti-TLR7-treated CS cessation groups were completely protected from alveolar septal damage and increased diameter, which were no different to levels in anti-TLR7-treated air-exposed controls. Most importantly, in both groups of anti-TLR7 treated CS-exposed mice alveolar septal damage and diameter were significantly and substantially reduced compared to isotype-treated continually CS-exposed and CS cessation controls. This was associated with reduced numbers of TUNEL^+^ parenchyma cells (Fig. 9m).

Next, we assessed the impact of therapeutic anti-TLR7 treatment on small airway remodelling. Small airway epithelial thickening (Fig. 9n, Supplementary Fig. 28b) and nuclei numbers (Fig. 9o) were increased in continually CS-exposed and CS cessation mice treated with either isotype or anti-TLR7 compared to their air-exposed controls. Notably, anti-TLR7 treatment reduced small airway epithelial cell thickening and nuclei number in CS-exposed compared to isotype-treated controls.

We recently established the assessment of pulmonary gaseous exchange in mice in terms of diffusing lung capacity for carbon monoxide (DL_CO_) similar to that assessed in COPD patients^34^. Isotype-treated continually CS-exposed and CS-cessation mice had reduced gas exchange compared to isotype-treated air-exposed controls (Fig. 9p). Anti-TLR7 treatment completely restored gas exchange in the lungs of continually CS-exposed and CS-cessation mice back to baseline levels in air-exposed mice.

Isotype-treated continually CS-exposed and CS cessation groups also had increased mast cell numbers compared to isotype-treated controls (Fig. 9q). Anti-TLR7 treatment completely inhibited the increases in mast cell numbers back to anti-TLR7-treated air-exposed control levels, and were substantially reduced compared to isotype-treated CS-exposed controls. The levels of BALF inflammatory cells were reduced in anti-TLR7 treated continually CS-exposed mice compared to treatment with isotype control (Supplementary Fig. 29a-d). Treatment did not alter histopathology scores in CS-exposed compared to isotype-treated controls (Supplementary Fig. 29e–i).

## Discussion

In this study, we have demonstrated non-viral related functions of TLR7, and shown pathogenic roles and potential for therapeutic targeting in lung damage. We show that TLR7 is increased in human and experimental COPD, and promotes alveolar destruction, emphysema, and experimental COPD through mast cell tryptase activity. This is unexpected because of the known roles of TLR7 are in antiviral immunity and protection against infection-induced exacerbations in respiratory diseases^8,13,14^. *Tlr7*^−/−^ mice had reduced CS-induced emphysema and airway remodeling and improved lung function in experimental COPD. These were associated with reduced apoptosis in the lungs. The mechanism of TLR7 regulating apoptosis remains unclear, and the involvement of active caspases in defining the role of apoptosis should be evaluated in future studies. Conversely, imiquimod stimulation of TLR7 induced emphysema and apoptosis in mouse lungs, which synergistically increased with CS exposure. Others showed that CS-exposed *Unc93b1* mutant (*Tlr3/7/9*^−/−^) mice also had significant reductions in alveolar enlargement^32^, however, the specific involvement of TLR7 was not elucidated. Imiquimod is pro-apoptotic against certain cancer cells^51–54^, and induces apoptosis in human and mouse cell lines^55^. Thus, our study is to demonstrate unexpected roles for TLR7 in apoptosis in the lung, emphysema, and experimental COPD.

Our CS-induced model of experimental COPD is well established and widely recognized in the field as representative of human exposures and disease. It is induced by inhaled exposures the same exposure method as human smokers^38–47^, is representative of the exposures of a pack-a-day smoker^38,43,46,56^, and has the same disease features and mRNA expression signatures in the lungs as human COPD. It is not confounded by ingestion of smoke residue or particles and induces similar disease features in both BALB/c and C57BL/6 mice. A further strength of this study is that we show supportive findings in both these strains, in genetically modified mice and in humans showing that the findings are not mouse or strain specific. Female mice were used since women are more susceptible (50% increased risk) of developing COPD and suffer more severe disease and mortality^57,58^. In the United States women account for 53% of COPD deaths and COPD is now the commonest cause of female deaths^59,60^. Also, logistically female mice are easier to house collectively. Future studies should also examine male mice.

Somewhat surprisingly, CS-induced pulmonary inflammation in BALF and tissues and histopathology scores were not altered in *Tlr7*^−/−^ mice. This is in contrast to *Unc93b1* mutant mice that had reduced total leukocytes in BALF when exposed to CS for six months^32^. However, the observed reduction in BALF total leukocytes in that study was minor ( ~ 1.3-fold)^32^, and the effects may be consequential of global dysfunction of intracellular TLR signaling. We found that CS-induced inflammatory mediators, chemokines and COPD-related factors^27,38,43^ were induced by CS exposure but were not altered in *Tlr7*^−/−^ mice. This was consistent with the inflammatory profile in *Tlr7*^−/−^ mice. We also showed that TLR7 stimulation with imiquimod or anti-TLR7 treatment did not alter CS-induced pulmonary inflammation. The majority of observations are consistent between anti-TLR7, imiquimod, and TLR7 deletion experiments. The only difference is the effect on inflammation. This may occur through different cells that are impacted or interactions with the TLR7 receptors in different situations, or compensatory effects that could occur in *Tlr7*^−/−^ mice. Hence, TLR7 does not have a major role in CS-induced inflammation, and the effects on emphysema and COPD are inflammation-independent. Instead, the effects are likely on the airway epithelium and we show small-airway remodeling changes and apoptosis may be linked to and promote emphysema^61^. We show that TLR7 is robustly expressed by epithelial cells in the lungs of COPD patients and in experimental COPD. This could be involved in the development of emphysema via effects on alveolar epithelial cells. The role of TLR7 on epithelial cells could be further assessed in a *Tlr7* floxed epithelial Cre system in future studies.

Low dose imiquimod (50 µg) was used to mimic responses induced by CS exposure rather than viral infection. Acute viral infections induce excessive inflammatory cell infiltrates, cytokine and chemokine responses and virus-induced tissue destruction^50–52^. Inflammatory responses induced by CS exposure are chronic and low grade, and CS suppresses anti-viral IFN responses in certain immune cells^38,62,63^. We and others previously showed that CS exposure suppressed IFN responses, which increased susceptibility to lung infections that typify COPD exacerbations^38,64–68^. Intranasal administration of higher doses (100 µg) of another TLR7 agonist, gardiquimod, also did not induce significant type I IFN responses in the lung^69^. Some mice were also intranasally administered a similar dose of imiquimod (50 µg), but the effects on lung type-I IFN responses were not assessed^69^.

In contrast, other studies demonstrated imiquimod-induced IFNs expression in other tissues^70–72^. Topical application of Aldara (62.5 mg, 5% imiquimod) induced psoriasiform skin inflammation and increased serum levels of IFN-α and -β in mice^70^. *Ifna2* mRNA levels were also induced in healthy human peripheral blood monocytes stimulated with imiquimod (10 µg/mL) in vitro^71^. Moreover, oral imiquimod administration (30 mg/kg) induced type-I *Ifn* mRNA levels in the gastrointestinal mucosa of mice^72^. The differences between our study and those of others may be in part due to differences in regimen, doses, route of administration, and cell-/tissue-specific effects. In our experimental studies we showed that IFN responses were not altered in *Tlr7*^−/−^ mice or with antibody neutralization of TLR7. Thus, targeting TLR7 in COPD may not predispose patients to an increased risk of infectious exacerbations.

We show that increases in mast cell numbers are important in COPD pathogenesis. They were substantially increased in the lungs of WT mice chronically exposed to imiquimod in association with the induction of chronic features of COPD. The absence of TLR7 and MyD88 prevented this increase and the development of CS-induced experimental COPD. Treatment with anti-TLR7 or genetic deletion of TLR7 also prevented the increase in lung MCs and the development of experimental COPD. Mast cell degranulation and specific mast cell tryptases are also important. Imiquimod induced the degranulation of human mast cells. Treatment with the mast cell stabilizer cromolyn or the absence of the granule-associated mast cell tryptase mMCP6 (equivalent of human tryptase-β) prevented the development of imiquimod-induced COPD features in vivo. Cromolyn treatment, which stabilizes mast cells and suppresses their degranulation, also prevented the development of experimental COPD and associated increases in mMCP6. Treatment with recombinant human tryptase-β induced the chronic features of COPD in vivo. However, imiquimod-induced disease features were not altered in the absence of another granule-associated mast cell tryptase PRSS31 (equivalent of human tryptase-γ). Imiquimod also did not affect the levels of other known protease (neutrophil elastase, myeloperoxidase, MMP activity) in the lung providing further evidence that the effects may be mast cell specific. Together these findings show that increases in mast cell numbers, their degranulation and release of specific granule tryptases are all important in COPD pathogenesis, and degranulation and tryptases may be therapeutically targeted in emphysema and COPD.

Mast cell tryptases are serine proteases that break down cellular substrates including the extracellular matrix^73^, and tight junction proteins occluding and claudin-5^74^, through protease-activated receptor-2 that could lead to emphysema. The substrates involved are yet to be fully elucidated. They also stimulate fibroblast proliferation and can induce collagen synthesis and fibrosis^75^ potentially around the airways, and increase the levels and activity of matrix metalloproteinases (MMPs)^73,76^ that cleave elastin also contributing to emphysema. Mast cell tryptases also induce leukocyte infiltration, particularly macrophages and neutrophils, which when activated release their cytotoxic mediators further contributing to emphysema^27^. There are limited studies on mast cell tryptase-β2 (*TPSB2*). One study showed that *TPSB2* mRNA expression is increased in the sputum of patients with COPD compared to healthy controls^77^.

TLR7 signaling has not been widely assessed in mast cells. Others showed that the TLR7 agonist resiquimod (R848) stimulated the synthesis and release of the cytokines IFN-α and -β and TNF, chemokines CCL3 and CXCL8 and pro-inflammatory lipid mediators from rat intra-peritoneal macrophages^78^. This could induce downstream inflammatory responses in COPD. In support of this, stimulation of the TLRs of mast cells results in the activation of MyD88 that promotes the nuclear translocation of NF-kB leading to the transcription of many genes, including cytokines, that have numerous downstream COPD-associated effects^79^. Some studies showed that ligands of TLR induce mast cell degranulation^80^, and our work shows for the first time that this and the release of mast cell tryptases may occur in association with TLR7 and MyD88. Precisely how TLR7 signaling and MyD88 activation regulate mast cell function to drive emphysema and COPD remain to be determined in future studies. We show that a subset of the TLR7 gene signature is contributed by epithelial cells involved in inflammatory responses that could drive mast cell responses. The remainder of the signature likely comes from immune cells such as mast cells. Mast cells express TLR7 and stimulation with the TLR7 agonist resiquimod induced their production of inflammatory cytokines and chemokines in vitro^24–26^. We previously showed that mice deficient in mast cell-specific tryptases mMCP-6 and Prss31 were protected against CS-induced emphysema and experimental COPD^27,28^. However, it was not known whether TLR7 activation on mast cells leads to the release of tryptases that promote disease. We therefore assessed the impact of imiquimod administration on *mmcp6*^−/−^ or *Prss31*^−/−^ mice. Imiquimod-induced emphysema was significantly ablated in *mmcp6*^−/−^, but not *Prss31*^−/−^ mice. mMCP-6 is soluble while Prss31 is a membrane-bound tryptase. This suggests that the effects of TLR7-induced emphysema are likely to involve soluble tryptases or mediators released from mast cells. Others showed that imiquimod stimulation of HMC-1 cells induced morphological changes, increases in intracellular vesicles, lipid droplets, and ribosomes, and downstream signaling with pro-inflammatory and angiogenesis responses but there was no effect on mast cell survival^81^. Furthermore, we showed that the human mast cell line HMC-1 expressed TLR7 and stimulation with imiquimod increased mast cell tryptase activity in culture supernatants. Collectively, our study demonstrates a role for TLR7 in mast cells and provides new insights into the mechanisms of TLR7-driven emphysema and COPD.

Our current and previous^34^ studies show that TLR7 is distributed in the cytoplasm but also on the plasma membrane and facilitates the uptake of antibodies, which could then function intracellularly. We found that TLR7 is located in the membranes and cytoplasm of human COPD bronchoepithelial cells and in the cytoplasm rather than the nucleus of epithelial and inflammatory cells in lung sections in experimental COPD. In the membrane, TLR7 could be acting as a typical TLR and upon stimulation inducing intracellular signaling. In the cytoplasm TLR7 could be working through endosomes to induce oxidative stress or through MyD88- and caspase-dependent pathways of apoptosis that drive inflammation and cell death as in other situations^8,82^. This needs to be further examined in future studies.

TLR7 may detect and induce inflammatory responses to host-derived RNA released by apoptotic cells^26,32,33,83^. It is activated and increased by viral and ssRNA including micro (miR)RNAs, and GU-rich and Au element RNAs^84–86^. We assessed the levels of circulating RNA indirectly by detecting the presence of anti-Smith antibody (a host RNA-specific auto-antibody)^34^. Serum anti-Smith antibody levels are inversely correlated with impaired lung function in COPD patients, suggesting that the levels of endogenous RNA are increased in COPD. Supporting this, we showed previously that CS exposure induced cell death^87,88^, which could release ssRNA to activate and potentially increase TLR7 levels. Others showed that mice exposed to CS for six months had increased levels of nucleic acids (RNA, DNA) in BALF^32^. This was likely a consequence of apoptosis and was also shown using the mouse lung epithelial cell line (MLE-15) exposed to CS extract in vitro^32^. Additionally, ssRNA stimulated TNF-α production in mouse macrophage (RAW-ELAM) and activated human monocyte-like (THP-1) cells in a TLR7-dependent manner^33^. We^47,89^, and others^90^, also showed that miRNAs have key roles in COPD pathogenesis. Extracellular miR-let-7b increases the levels of TLR7 in sensory neurons via direct binding and activation of this TLR^91^. miR-21, which we have shown is elevated in the lungs in experimental COPD^47^, has important roles in TLR7 activation^92^. Furthermore, TLR7 is highly expressed by macrophages^93^, and here we found that mast cells also express TLR7. In this and our previous studies, lung macrophages and mast cells^27,42,87^ are increased in experimental COPD. The increases in these cells that express TLR7 could also, therefore, result in increased levels of TLR7 in experimental COPD. Thus, increases in TLR7 levels and activation are likely caused by increased ssRNA levels and/or macrophages and mast cells expressing TLR7. Further investigation with a combination of TLR7 pulldown assays and mass spectrometry may be useful in determining whether CS interacts with TLR7. The impact of microbiomes and their component nucleic acids would also be interesting^56,94^.

There is the potential that increases in TLR7 increases channel function. Previous studies showed that the presence of TLR7 in tracheal submucosal gland cells resulted in a rapid attenuation of acetylcholine-induced Ca2^+^ dependent ion currents in the airways^95^. Other studies also demonstrated that imiquimod treatment increased intracellular Ca2+ flux in neurons^96^, and activation of TRL7 simulated Ca2^+^ flux in monocytes that protected against RNA virus infection^97^. The extracellular miRNA let-7b can cause excitation of sensory neurons via direct binding and activation of TLR7^91^, and blockade of TLR7 inhibits acetylcholine-induced ion currents in tracheal submucosal gland cells^95^. These studies suggest that increases in TLR7 may be associated with altered Ca2+ channel function in COPD but this needs to be investigated further.

Here we make major progress in defining new mechanisms of emphysema and COPD. There are limitations and areas for future study. Further evidence for cause and effect could be provided using conditional *Tlr7*^−/−^ deletion mice but these do not currently exist. It remains unknown exactly why TLR7 increased and how TLR7, MyD88, and mMCP-6/hTrypase-β induce degranulation and emphysema and these mechanisms need to be defined. This could be achieved using RNA-sequencing and proteomics over a time course. Our studies were only performed in female mice and further studies in males would show any sex differences. We have not defined definitively what stimulates TLR7 to induce the effects. Trypase-β is the most abundant component of mast cell granules but the roles of the other factors contained in these granules in the processes that we describe remain unknown. The precise roles and relative contributions of TLR7 on epithelial cells and intracellularly need to be defined. Finally, future clinical trials of TLR7 neutralizing antibodies, and inhibitors of human tryptase-β should be undertaken in patients with COPD to determine the clinical benefit and their potential as therapeutics. We found increased TLR7^+^ mast cells in COPD, that correlated with reduced lung function, indicating that TLR7^+^ mast cells are associated with the severity of the COPD. It is difficult to identify TLR7^+^ mast cells in different stages of COPD, due to their low numbers of mast cells in the lung and large sample sizes would strengthen these findings.

In summary, we discover an unexpected role for TLR7 in mediating emphysema and COPD through mast cell-specific tryptase activity. In COPD, TLR7 expression is increased on the surface and cytoplasm of mast cells and induces mast cell influx, activity, and degranulation. It is also increased lung epithelial cells and macrophages. TLR7 may detect and induce inflammatory responses to host-derived RNA released by airway and/or alveolar epithelial cell death^37,64,65^. This could involve the trafficking of NF-kB to the nucleus and stimulation of mRNA expression, but this needs to be elucidated in future studies. Mast cell degranulation releases tryptases that break down cell substrates and tight junctions, which induces airway fibrosis and MMPs that cleave elastin. They also induce macrophage influx, inflammatory responses, and release of cytotoxic mediators. In combination, these effects over time and with repeated exposures can contribute to the development of chronic features of emphysema and impaired lung function in COPD. CS- and imiquimod-induced emphysema is TLR7- and MyD88-dependent. Inhibiting TLR7 reduces mast cells and their activation and mediator release suppresses the development of COPD (Fig. 9r). Our study provides new insights and possible mechanisms of how TLR7 potentiates tissue-specific responses through mast cells, and shows that prophylactic and therapeutic targeting of TLR7 reduced lung mast cell numbers, emphysema and experimental COPD. Thus, our study defines TLR7 as a promising treatment target and opens a new avenue for developing new therapies to reduce or reverse the severity of emphysema and COPD, as well as mast cell-related diseases.

## Methods

### Ethics statement

The Institutional Review Board of the University of Arizona approved the study (IRB #1811124026). The human study was also approved by the medical ethics committee of the Ghent University Hospital (2016/0312 and 2019/0537) and the “delibera C.S. n.270 of the 18th of October 2017” of the Ethics Committee of the Academic Hospital of Messina, Italy (www.polime.it). All recruited volunteers provided written informed consent.

Animal work in this study was performed in accordance with the recommendations issued in the Australian Code of practice for the care and use of animals for scientific purposes by the National Health and Medical Research Council of Australia. All protocols were approved by the Animal Ethics Committees of The University of Newcastle, Australia (A-2008-100) and Sydney Local Health District (SLHD) (2018/004).

### Microarray analysis of differential human gene expression

Differential gene expression analysis of published datasets (Gene Expression Omnibus [GEO] accession numbers GSE5058 and GSE27597)^29–31^ was performed using Array Studio software (Omicsoft Corporation, Research Triangle Park, NC) from a general linear model adjusting for age and gender and the Benjamini–Hochberg method for *P*-value adjustment as described previously^98–100^. Patients’ demographic and clinical data of donors for GSE5058 and GSE8545 were shown in Supplementary Tables 1 and 2. Expression analysis of the same datasets was performed with Limma^101^ using the log2 TMA-normalised intensities from GSE27597 to identify differentially expressed genes (DEGs) (FDR < 0.05) between healthy and COPD samples. Hierarchical clustering was used to identify gene clusters based on their expression profiles. Enriched functions of the clusters were annotated using ViSEAGO^102^. To identify the *TLR7* gene signature, pairwise correlation was performed using the gene cluster with *TLR7* to shortlist the gene expression that strongly and positively correlated with TLR7 (Pearson r ≥ 0.6; FDR < 0.05). The expression of the *TLR7* gene signature was validated with the datasets from microdissected COPD small airway epithelium (GSE5058)^30^. To examine the epithelial contribution associated with TLR7 signaling, RNA-seq data of airway epithelial organoids from healthy and COPD individuals were obtained (GSE201465, GSE186017) and analyzed with DESeq2^103^ and Seurat^104^.

### Robust microarray analysis (RMA) of differential human gene expression

DEG analysis was performed using the RMA algorithm and published datasets (Gene Expression Omnibus [GEO] accession numbers GSE5058 and GSE8545)^62,63,105^. This was performed using Array Studio software (Omicsoft Corporation, Research Triangle Park, NC) from a general linear model adjusting for age and gender and the Benjamini–Hochberg method for *P*-value adjustment as described previously^54^. Demographic and clinical data of donors for microarray studies GSE5058 and GSE8545 are in Supplementary Tables 1 and 2.

### Lung single-cell RNA-sequencing COPD dataset

We analyzed the expression of *TLR7* in different immune cells in previously published single-cell RNA-sequencing datasets from severe COPD patients and healthy control^106^. The data was explored using the COPD Cell Atlas online portal (www.copdcellatlas.com). The *TLR7* gene was searched under Gene Explorer on the website, immune cell type was selected as a category and the plot type was violin.

### Endogenous RNA quantification

The amount of anti-Smith antibody, was quantified using anti-Sm/RNP-C (IgG) enzyme-linked immunosorbent assay (ELISA, 35-SNRHU-E01, ALPCO Diagnostics, Salem, NH) in the serum of patients with clinically diagnosed COPD from the Advair Biomarkers in COPD (ABC) trial study (Supplementary Table 6)^35^. Serum anti-Smith antibody levels were then tested for correlation with lung function (forced expiratory volume in 1 second, FEV_1_ % predicted). Study participants were diagnosed as having COPD based on an average smoking of 10 pack-years and post-bronchodilator FEV_1_: forced vital capacity (FVC) ratio of less than 70%. Participants were randomly selected and stratified by quantiles of FEV_1_. Samples were run in duplicate and the coefficient of variance (CV) determined. The dynamic range was 0–300 U/mL and analytical sensitivity of the kit was 1.0 U/mL. The Advair clinical trial was registered with www.clinicaltrials.gov (NCT00120978).

### Animals

Female, 7-8-week-old, wild-type (WT) controls, Toll-like receptor (TLR)7-(*Tlr7* ^−/−^) and MyD88-deficient (*Myd88*^−/−^) BALB/c mice^107^ were originally from Prof. Shizhu Akirap Osaka University, Japan and provided to the Matters’ lab at the University of Newcastle, Australia. Mouse mast cell protease-6-deficient (*mmcp6*^−/−^) C57BL/6 mice^27^ and protease serine member S31-deficient (*Prss31*^−/−^) C57BL/6 mice^24^ were a gift from Prof. Rick Stevens, Brigham and Women’s Hospital, Harvard University and were then maintained at Australian BioResources facility (Moss Vale, NSW, Australia). WT BALB/c mice were used as control for *Tlr7*^−/−^ BALB/c mice, and WT C57BL/6 mice were used as controls for *mmcp6*^−/−^ and *Prss31*^−/−^ mice. All animals were bred and housed in a specific pathogen-free barrier in the BioResources Facility at the Hunter Medical Research Institute (New Lambton Heights, NSW, Australia) or Centenary Institute (Camperdown, NSW) at 18–23 °C with 40–60% humidity under a 12-hour light and dark cycle. Experimental control animals were co-housed in the same room as experimental groups. Animals had free access to sterile food and water. Cages were chosen at random for treatments with or without exposure to normal air or cigarette smoke (CS). Power was not explicitly calculated for each experiment. Numbers of mice were typically used based on knowledge from prior experiments and publications. CS exposure of mice was performed by research technicians who were blinded to the study.

### Murine model of experimental COPD

Age- and sex-matched 7-8-week-old mice (female, *n* = 8/group) were randomly exposed to normal air or CS via the nose only for up to 12 weeks as extensively described previously^27,28,37–47,108^. Briefly, mice were simultaneously exposed to CS (twelve 3R4F reference cigarettes, University of Kentucky, Lexington, KY, twice per day, 5 times per week, for up to 12 weeks) using a custom-designed and purpose-built nose-only, directed flow inhalation and smoke-exposure system (CH Technologies, Westwood, NJ) housed in a biosafety cabinet. Each exposure typically lasted for 75 min.

### In vivo activation of TLR7

Some mice (female, 6–8 weeks old, *n* = 8) were administered 50 μg of TLR7 agonist imiquimod (clone R837, Invivogen, San Diego, CA) in 50 μl sterile Dulbecco’s phosphate-buffered saline (PBS, Life Technologies, Mulgrave, Victoria, Australia)^69^, intranasally (i.n) under isoflurane-induced anesthesia, 5 times per week, either for 2 or 8 weeks in the absence of CS exposure or between weeks 6–8 of CS exposure. Controls received sterile saline. Some WT BALB/c mice (female, 6–8 weeks old, *n* = 6–8) were administered imiquimod (100 μg in 50 μl sterile saline), i.n., 5 times per week, for 2 weeks. Controls received sterile saline.

### In vivo mast cell stabilization

Some mice (female, 6–8 weeks old, *n* = 8) were administered 50 mg/kg of body weight of cromolyn sodium salt^49^ (C0399, >95% purity, Sigma Aldrich/Merck, Castle Hill, New South Wales, Australia) in 50 μl sterile ultrapure water, intranasally (i.n) under isoflurane-induced anesthesia, 5 times per week, for up to 2 weeks. Controls received sterile saline.

### Mast cell inhibitor treatment

Mice (female, 6–8 weeks old, *n* = 8) were exposed to CS for 8 weeks, control mice were exposed to normal air. Some mice were treated with 50 mg/kg disodium cromoglycate (DSCG, cat# 15826-37-6, Sigma) intranasally from weeks 6 to 8 of CS exposure, and control mice received equal volumes of vehicle. BALF was collected for assessment of inflammatory cell influx, lungs were perfused, formalin-fixed, paraffin-embedded and sectioned (3.5 μm thickness) for histology, stained with H&E for emphysema analysis, and lung function was assessed using the flexiVent system^27,28,42,46^. Proteins were extracted from mouse lungs and mMCP6 levels were measured by immunoblot^41^.

### Protein extraction and immunoblot

Mice were exposed to the smoke of 12 cigarettes, twice per day, 5 days per week for 8 weeks, and control mice were exposed to normal air. Lung tissues were collected, and proteins were extracted with RIPA buffer supplemented with PhosSTOP phosphatase and protease inhibitors (Roche). Tissues were homogenized and centrifuged (8,000xg, 4 °C, 10 min) for immunoblot^41^. Proteins were separated by SDS-PAGE using Mini-PROTEAN TGX stain-free gels (Bio-Rad) and transferred to PVDF membranes (Bio-Rad)^109–112^. Blots were incubated with TLR7 (1:2,000, NBP2-24906, Novus Biologicals), mMCP6 (1:1,000, MAB3736, R&D Systems) or β-actin (1:10,000, ab8226, Abcam) antibody (4 °C, overnight). Blots were then incubated with anti-rabbit or anti-mouse secondary antibody (R&D system, room temperature, 2 hr). Images were captured using a ChemiDoc MP System (Bio-Rad). Densitometry analysis was performed using image J and presented as fold change of target protein normalized to β-actin^109,111^.

### In vivo neutralization of TLR7 with monoclonal antibody

Some mice (female, 6–8 weeks old, *n* = 8) were administered neutralizing anti-TLR7 (clone Ba/F3; 4 mg/kg of body weight) monoclonal antibody or IgG1/κ isotype control^34,113^, by i.v. injection under isoflurane-induced anesthesia, once per week, during the last 2 weeks (between weeks 6–8) or 4 weeks (between weeks 8–12) of continued CS exposure or CS cessation.

### Isolation of RNA

Total RNA was extracted from whole lung tissue or blunt-dissected airways and parenchyma as described previously^43,114^. Briefly, the trachea and lungs were excised, and the airways carefully separated from the lung parenchyma with sterile forceps. Whole lungs, airways, and parenchyma were then snap-frozen and stored at -80 °C. Tissues were thawed and homogenized in 500 μL of sterile PBS (Life Technologies, Mulgrave, Victoria, Australia) using a Tissue-Tearor stick homogenizer (BioSpec Products, Bartesville, OK) on ice. Total RNA was extracted using TRIzol reagent (Invitrogen, Mount Waverly, Victoria, Australia) according to the manufacturer’s instructions and stored at −80 °C^43^.

### Real-time quantitative polymerase chain reaction (qPCR)

Total RNA from whole lungs, airways and parenchyma (1000 ng) were reversed-transcribed using Bioscript reverse transcriptase (Bioline, Alexandria, New South Wales, Australia) and random hexamer primers (Invitrogen) as described previously^115–118^. The mRNA levels of cytokines, chemokines, COPD-related factors and interferon-related factors were determined by qPCR (ABIPrism7000, Applied Biosystems, Scoresby, Victoria, Australia). Assays were performed using SYBR Green Supermix (KAPA Biosystems Inc., Wilmington, MA), normalized to the house-keeping hypoxanthine-guanine phosphoribosyltransferase (*Hprt*) transcript and expressed as relative abundance to normal air-exposed WT controls. Custom-designed primers (Integrated DNA Technologies, Baulkham Hills, New South Wales, Australia) were used (Supplementary Table 8). Primers used to assess mast cell chemokines and TLR7 downstream molecules (KiCqStart^TM^, Sigma-Aldrich) are listed in Supplementary Tables 9 and 10.

### Immunohistochemistry of human lungs

For the immunohistochemical analysis, 5‑micron thick sections of formalin-fixed paraffin-embedded tissue blocks were deparaffinized, washed in descending alcohol concentrations, treated with 3% hydrogen peroxide for 10 min, washed again three times in deionized water and incubated (room temperature, 30 min) with normal goat serum to prevent nonspecific binding of serum proteins. Then, using the ULTRA Staining system (Ventana Medical Systems), sections were washed with deionized water and incubated (37˚C, 30 min) with primary rabbit polyclonal anti-human TLR7 antibody (dilution 1:100, Proteintech, code 17232-1-AP, Supplementary Table 11). Slides were washed three times with PBS and incubated (room temperature, 30 min) with biotinylated goat anti‑rabbit IgG horseradish peroxidase‑labeled secondary antibody (1:300; Abcam). Finally, staining was developed with diaminobenzidine tetrahydrochloride and counterstained with haematoxylin. Negative controls omitted the specific antisera and substituted PBS for the primary antibody. Immunostained slides were evaluated using light microscopy with a ×20 and ×40 objective lens and ×10 eyepiece. Two certified pathologists using a double-headed microscopes performed the assessment on a consensus basis. The percentage of the different (alveolar macrophages/immune cells, bronchiolar epithelial cells, and pneumocytes) immunostained cells (area of staining positivity: ASP) were graded as follows: 0 (no staining); 1 ( > 0 to 5%); 2 ( > 5 to 50%) and 3 ( > 50%). In addition, the intensity of staining (IS) (weak=1; moderate=2; strong=3) was also taken into consideration. An intensity-distribution (ID) score was calculated for each case by adding the values of the ASP and IS, only cases showing an ID score of >3 were considered positive. The project was approved by the “delibera C.S. n.270 of the 18^th^ of October 2017” of the Ethic Committee of the Academic Hospital of Messina, Italy (www.polime.it).

### Immunohistochemistry of mouse lungs

Lungs were perfused, inflated, formalin-fixed, paraffin-embedded, and sectioned (4–6 μm). Longitudinal sections of the left lung were kept on a heating block at 70 °C for 15 min, rehydrated through a series of xylene (2x) and ethanol gradient (2x absolute, 90%, 80%, 70%, 50%, 0.85% saline and PBS) washes, followed by antigen retrieval with citrate buffer (10 mM citric acid, 0.05% Tween 20, pH 6.0) in a steam cooker for 30 min. Sections were blocked with a casein blocker (Thermo Scientific) at room temperature for 1 h. Sections were then washed with PBS (5x 5 min) and incubated with primary anti-TLR7 rabbit antibody^119,120^ (1:100, ab45371, Abcam, Melbourne, Victoria, Australia) or anti-fibronectin rabbit antibody (1: 100 ab2413, Abcam, Melbourne, Victoria, Australia) overnight at 4 °C, washed with PBS (5x, 5 min each), followed by anti-rabbit horseradish peroxidase-conjugated secondary antibody incubation at 37 °C for 30 min (R&D Systems, Gymea, New South Wales, Australia) as per manufacturer’s recommendations. The 3,3’-diaminobenzidine chromogen-substrate buffer (DAKO, North Sydney, New South Wales, Australia) was applied to sections and incubated in the dark at room temperature for ~12 min. Sections were washed in PBS (5x, 5 min each), counterstained with hematoxylin (5 min), dehydrated, mounted, and analyzed with a BX51 microscope (Olympus, Tokyo, Shinjuku, Japan) and Image-Pro Plus software (Media Cybernetics, Rockville, MD).

### Immunofluorescence for human lung sections

The Institutional Review Board of the University of Arizona approved the patient recruitment protocol (2019–2021). All patients had complete medical history and lung function tests (spirometry); they also underwent whole-lung volumetric computed tomography (CT) at study entry and at Year 5, permitting longitudinal assessment of lung CT characteristics, expressed in %LAA950 scores. From the Polverino Lung Biorepository, a total of 21 FFPE lung sections from never-smoker (NS) controls (*n* = 4), smoker (*n* = 6), and COPD patients (*n* = 11, GOLD I, II and IV, Supplementary Table 7) were assessed. Donors either underwent surgery for solitary pulmonary nodule removal and the lung samples were collected 10 cm away from the nodule, or underwent lung transplant for severe COPD. Lung sections were deparaffinized and incubated with 10 mM citrate buffer (pH 6.0, 100˚C, 10 min) for antigen retrieval, and blocked with 1% albumin and 5% normal goat and rabbit serum (room temperature, overnight). Sections were incubated with IgG rabbit anti-human Tryptase (1:75, #AB196772, Abcam, Cambridge, MA, room temperature, 1 hr), followed by IgG mouse anti-human TLR7 (1: 50, MBS668010, MyBiosource, San Diego, CA, 4 °C, overnight). Sections were then stained with rabbit anti-mouse-Alexa488® and goat anti-rabbit-Alexa555® (Invitrogen, Carlsbad, CA, both 1:100, 37 °C, 1 hr), or non-immune mouse or rabbit IgG (Agilent, Carpinteria, CA) applied at the same concentration for the negative controls. Nuclei were counterstained with DAPI mounting gel (Abcam, Cambridge, MA). Twenty non-consecutive images were captured with an Olympus® BX43 microscope with a digital camera (DP80, Olympus®, PA, USA), at 100X magnification with submersion oil (Immoil-F30CC, Olympus ®, Japan). Metamorph® Software was used to quantify Tryptase^+^ and TLR7^+^ cells. Data were analyzed SigmaPlot™ software (Systat, San Jose, CA), version 14.0.

### Immunofluorescence for mouse lung sections

Mice were exposed to CS for 8 weeks and control mice breathed normal air. Lungs were perfused, formalin fixed, embedded and sectioned (3.5 μm thickness) for histology. Lung sections were incubated with sodium citrate buffer (10 mM sodium citrate, 0.05% Tween 20, pH 6.0, 100 °C, 35 min) for antigen retrieval, and blocked with 5% BSA (room temperature, 1 hr). Slides were stained with TLR7 (1:100, NBP2-24906, Novus Biologicals) and CD8 (1:100,14-0808-82, ThermoFish Scientific) antibodies (4 °C, overnight). This was followed by anti-rabbit secondary antibody conjugated with Alexa Fluor® 488 (ab150077, Abcam) and anti-rat secondary antibody conjugated with Alexa Fluor® 647 (room temperature, 1 hr). Nuclei were stained with DAPI. Six random images per slides were taken using an Axio Imager M2 microscope and analyzed using Zen imaging software^110^. mMCP4 is a major marker of mast cells^121^ in mice and F4/80 is a macrophage marker. Lung sections from WT mice that developed experimental COPD after 8 weeks of CS exposure and air-exposed controls were stained with mMCP4 (ab92368, Abcam), TLR7 (NBP2-24906, Novus), F4/80 (conjugated with PE, 565410, BD) and DAPI. A far-red label conjugated secondary antibody was used to stain mMCP4 and an FITC labeled antibody was used to stain TLR7. At least 30 random images were assessed from each section under 40x magnification using an Axio Imager M2 microscope (Zeiss). The numbers of TLR7^+^ mast cells (TLR7^+^mMCP4^+^) and TLR7^+^ macrophages (TLR7^+^F4/80^+^) were quantified.

### Airway and parenchymal inflammation

Airway inflammation was assessed by differential enumeration of inflammatory cells in cytospin preparations from BALF as described previously^43,118,122^. Briefly, BALF was collected by two 500 µl lung lavages with Hank’s Balanced Salt Solution (Life Technologies) through a cannula inserted into the trachea. BALF was centrifuged (527 x*g*, 8 min, 4 °C using Heraeus Multifuge X3 Centrifuge with TX-1000 Swinging Bucket Rotor [ThermoFisher Scientific, Scoresby Victoria, Australia]), the resulting supernatant collected, and red blood cells were lysed using lysis buffer (1 ml, Tris-buffered NH_4_Cl). Lysis buffer was then diluted with approximately 3 ml of Hank’s Balanced Salt Solution (Life Technologies) and centrifuged. The resultant supernatant was then decanted, and cell pellets were re-suspended in Hank’s Balanced Salt Solution. Total leukocytes were determined using a hemocytometer. Cells were cytocentrifuged and stained with May-Grunwald-Giemsa. Differential leukocyte counts were enumerated according to morphological criteria (250 cells by light microscopy using a BX51 microscope, Olympus, at 40x magnification)^123^. All slides were coded, and counts performed in a blinded manner.

### Histopathology

Histopathology was assessed at 10x and 40x magnification in longitudinal lung sections stained with hematoxylin and eosin (H&E) and scored based on a set of custom-designed criteria as described previously^28,123^. Slides were coded and assessments were performed in a blinded manner.

### Airway remodeling

Longitudinal sections of the left single-lobe lung were stained with H&E or Sirius red and fast green. Airway epithelial area (μm^2^) and cell (nuclei) number were assessed at 40x or 100x magnification, quantified from a minimum of 3 small airways per lung section from each mouse using ImageJ software (Version 1.50, NIH, New York City, NY) and normalized to basement membrane (BM) perimeter (μm) as described previously^42,110^. Slides were coded and quantifications were performed in a blinded manner.

### Alveolar enlargement

Mouse lungs were perfused with 0.9% saline. The left lobes of the lung were tied off and the right lobe was perfused by inserting a 19-gauge needle to the heart and washed with 0.9% saline by gravity at 30 cm H_2_O height. A blunt needle was inserted into the mouse trachea and gently injected 500 ul 10% formalin to inflate the lung. The needle was removed, the trachea was then tied off and the lungs were fixed in 5 ml tubes with 10% formalin for 24 hrs. Longitudinal sections (4–6 μm thick) of the left single-lobe lung were stained with H&E to assess alveolar septal damage using the destructive index^124^, and alveolar size and diameter using the mean linear intercept technique, respectively^27,38^. Briefly, 40 randomized images of lung sections at 40x magnification were taken using the BX51 microscope (Olympus) and Image-Pro Plus software (Media Cybernetics). Images with partial lung sections (the edge of the lung section) or that contained multiple airways, blood vessels, and areas of inflammation and fibrosis were excluded from the counts. Ten viable images were then superimposed with destructive index or mean linear intercept grids using ImageJ software (Version 1.50, NIH), and counts were made in a blinded manner and averaged per lung section for each mouse^43^.

### Apoptosis

Longitudinal sections of the left single-lobe lung were stained with terminal deoxynucleotidyl transferase dUTP nick end labeling (TUNEL) assay kits (Promega, Sydney, New South Wales, Australia) according to the manufacturer’s instructions. Apoptosis in lung parenchyma was assessed by enumerating the numbers of TUNEL^+^ cells in 20 randomized, high-powered fields (fluorescent microscopy using the BX51 microscope [Olympus] at 100x magnification) in a blinded fashion and averaged per lung section for each mouse^31^. Cells were defined as apoptotic when superimposed images of cells stained with TUNEL (green) overlapped with nuclei stained with Bisbenzimide H 33342 (blue, Sigma Aldrich/Merck). TUNEL^+^ inflammatory cells or cells in alveolar spaces were excluded from the counts (verified by light microscopy using the BX51 microscope [Olympus], 100x magnification)^43^.

### Lung function

Mice were anaesthetised with a mix of xylazine (2 mg/ml) and ketamine (40 mg/ml), and cannulas were inserted into their tracheas by tracheostomy. Cannulated tracheas were attached to flexiVent apparatus (FX1 System; SCIREQ, Montreal, Canada). Mice were ventilated with a tidal volume of 8 mg/kg at a respiratory rate of 450 breaths/minutes, then transpulmonary resistance was assessed and recorded using flexiWare software (FX1 system, SCIREQ, Montreal, Canada)^41,45^. All assessments were performed at least three times and the average was calculated for each mouse. In some experiments gas exchange assessment in terms of the diffusing factor for carbon monoxide (DLCO) was assessed prior to the measurement of baseline lung function. A single-breath maneuver to assess the diffusing factor for carbon monoxide (DLCO) was performed^45^. Briefly, the lungs were inflated with 0.7 mL of tracer gas mixture (0.318% Ne, 0.302% CO, balance was air). Following a 9-second breath hold, 0.7 mL of gas was withdrawn from the lung, diluted to 2 mL (total volume) with room air, and the concentrations of Ne and CO were measured by gas chromatography (60-second gas analysis time; Micro GC Fusion® Gas Analyzer, INFICON, Singapore). DFCO is expressed as a value between 0 and 1; complete uptake of CO = 1, and no uptake =0.

### Enumeration of mast cells in lung tissues

Mouse lungs were perfused, inflated, formalin fixed, embedded and sectioned at 4 μm thickness. Longitudinal sections of the left single-lobed lung were stained with toluidine blue for mast cells. To quantify mast cells, >50 images per lung section were taken under x40 magnification using a light microscope (BX51 microscope from Olympus). The number of mast cells were enumerated in 6–8 complete longitudinal sections in a blinded fashion (light microscopy using the BX51 microscope [Olympus] at 20x or 40x magnification) and represented as numbers of mast cells per lung section for each mouse^42^.

### Bone marrow-derived mast cell transfers

Bone marrow cells were isolated from the femurs of WT and *Tlr7*^−/−^ mice, and cultured in RPMI media containing 10% FBS and 10 ng mouse IL-3 recombinant protein (Cat# I4144, Sigma-Aldrich) for 4 weeks as previously described^125^. Five x10^5^ WT or *Tlr7*^−/−^ mast cells in 50μl RPMI were transferred into WT BALB/c mice (female, 6–8 weeks old, n = 6, Supplementary Fig. 30). Control mice (female, 6–8 weeks old, *n* = 6) received the same volume of cell culture media only. Bronchioloalveolar lavage fluid (BALF) and lung tissues were collected 1 and 3 days after mast cell challenge.

### Tryptase-β challenge

Mice (6–8 weeks old, female, *n* = 5) were administered recombinant human tryptase-β (20 μg in 50 μL PBS/mouse, Cat# G5631, Promega) i.n. as previously described^27^. Control mice received equal volumes of PBS. Seven days after the challenge BALF and lungs were collected, and lung function assessed.

### Human mast cell culture

Cells of the human mast cell line (HMC)-1 were cultured in Dulbecco’s modified Eagle medium (DMEM, D5671, Sigma Aldrich/Merck) containing 10% fetal bovine serum (Bovogen), 25 mmol HEPES buffer, 100 U/ml penicillin, and 100 μg/ml streptomycin (37 °C, 5% CO_2_). HMC-1 cells were seeded at 500,000 cells per well and stimulated with imiquimod (5, 10, or 100 ng, Invivogen) in 50 μl DMEM supplemented with 0.5% fetal bovine serum or medium only (control) for 1 h. Cells were centrifuged (234 x *g*, 5 min, room temperature [Heraeus Multifuge X3 Centrifuge]), and resulting supernatants were collected. Cell pellets were then resuspended in 150 µL DMEM, cytocentrifuged and air-dried overnight prior to immunocytochemistry.

### TLR7 and mast cell tryptase immunocytochemistry

Cells were fixed with methanol (−20 °C, 15 min) and blocked with 1% bovine serum albumin (Sigma Aldrich/Merck) at room temperature for 1 h. Sections were then washed with PBS (5x, 5 min each) and incubated with primary anti-TLR7 rabbit antibody (1: 100, ab45371, Abcam) or anti-mast cell tryptase antibody (1:100, ab2378, Abcam) overnight at 4 °C, washed with PBS (5x, 5 min each), followed by anti-rabbit (1:200, R&D Systems, Gymea, New South Wales, Australia) or anti-mouse (1: 200, ab97023, Abcam) horseradish peroxidase-conjugated secondary antibody incubation at 37 °C for 1 h as per manufacturer’s recommendations. The DAKO buffer was applied to sections that were incubated in the dark at room temperature for ~12 min. Sections were washed in PBS (5x, 5 min each), counterstained with hematoxylin (5 min), air-dried and mounted. Twenty randomized images at 40x magnification were taken per slide using the BX51 microscope and Image-Pro Plus software. For quantification of mast cell tryptase, 10 viable images (x40) were randomly selected and the numbers of HMC-1 cells were enumerated in each image. DAB chromogen and hematoxylin signals were then separated and converted into pixels using the ImageJ Color Deconvolution plugin (NIH). DAB signal was quantified as the area (pixels) normalized to the number of HMC-1 cells in the image. In addition, DAB signal is also presented as a percentage area of the hematoxylin-stained area of HMC-1 cells.

### Human mast cell tryptase activity

Mast cell tryptase activity in the culture supernatant was determined using mast cell degranulation assay kits (Chemicon, Merck). Briefly, culture supernatants (20 μl) from HMC-1 cells incubated with media or imiquimod (5, 10, or 100 ng) were mixed with assay buffer (160 μL) and labeled substrate tosyl-gly-pro-lys-*p*NA (20 μl). Samples were incubated for 1 h at 37 °C. The chromophore *p*-nitroaniline (*p*NA) is cleaved from the labeled substrate by mast cell tryptase in the sample. The remaining free *p*NA is then determined using a microtiter plate reader (SpectraMax M5, Molecular Devices, CA, USA) at 405 nm. Optical density values were obtained, compared to known concentrations of a *p*NA standard curve and relative mast cell tryptase activities were defined.

### Statistical analyzes

Data are presented as means ± standard error of the mean (s.e.m.) from at least two independent experiments. Mean values appeared to be normally distributed and appropriate statistical tests were performed for each figure. The variance between the groups that were compared statistically appeared to be similar. Comparisons between the two groups were assessed using the two-tailed Mann-Whitney test. Comparisons between multiple groups were made using one-way ANOVA with Bonferroni’s multiple comparison test. Correlation analyzes were made using Spearman’s rank correlation coefficient test. Adjusted P values were calculated, and minimum statistical significance was accepted at *P* < 0.05. Early death was used as an exclusion criterion for animal experiments. However, no animals died in any of the experimental protocols. Significant outliers were identified using Grubb’s test and excluded from statistical analyzes. All statistical analyzes were performed using GraphPad Prism Software version 8 (San Diego, CA).

### Reporting summary

Further information on research design is available in the Nature Portfolio Reporting Summary linked to this article.

### Supplementary information

Supplementary Information

Reporting Summary

### Source data

Source Data

## Data Availability

All the datasets in this study are existing published and are available via the NCBI website, including Gene Expression Omnibus (GSE) accession number: GSE5058, GSE27597, GSE8545, GSE201465, GSE186017. Single cell RNA-sequencing COPD dataset is from the COPD Cell Atlas (www.copdcellatlas.com). All data are included in the Supplementary Information or are available from the authors, as are unique reagents used in this Article. The raw numbers for charts and graphs are available in the Source Data file where appropriate. Source data are provided with this paper.
