# Supplementary material for: TLR7 promotes smoke-induced experimental lung damage through the activity of mast cell tryptase
Source: Nat Commun. 2023 Nov 14;14:7349. doi: 10.1038/s41467-023-42913-z (PMC10646046; doi:10.1038/s41467-023-42913-z)
Supplement: Supplementary file 1 — Supplementary Information [file 41467_2023_42913_MOESM1_ESM.pdf]

TLR7 promotes smoke-induced experimental lung damage through the activity of mast cell tryptase

Gang Liu[#], Tatt Jhong Haw[#], Malcolm R. Starkey[#], Ashleigh M Philp, Stelios Pavlidis, Christina Nalkurthi, Prema M. Nair, Henry M. Gomez, Irwan Hanish, Alan CY. Hsu, Elinor Hortle, Sophie Pickles, Joselyn Rojas-Quintero, Raul San Jose Estepar, Jacqueline E. Marshall, Richard Y. Kim, Adam M. Collison, Joerg Mattes, Sobia Idrees, Alen Faiz, Nicole G. Hansbro, Ryutaro Fukui, Yusuke Murakami, Hong Sheng Cheng, Nguan Soon Tan, Sanjay H. Chotirmall, Jay C. Horvat, Paul S. Foster, Brian GG. Oliver, Francesca Polverino, Antonio Ieni, Francesco Monaco, Gaetano Caramori, Sukhwinder S. Sohal, Ken R. Bracke, Peter A. Wark, Ian M. Adcock, Kensuke Miyake, Don D. Sin, Philip M. Hansbro[*]

[#]Authors contributed equally

*Correspondence should be addressed to Philip M Hansbro
Email: philip.hansbro@uts.edu.au

SUPPLEMENTARY FIGURES

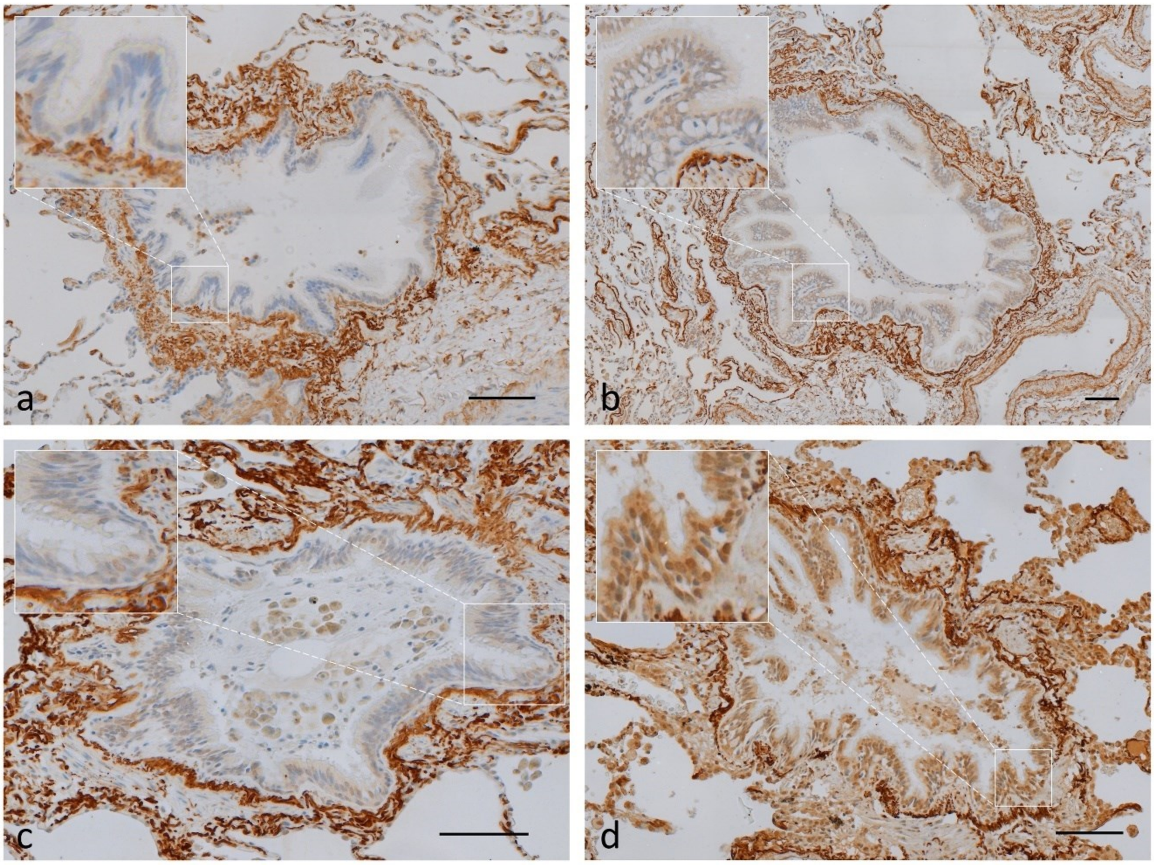

Supplementary Fig. 1 | TLR7 protein in human airways. Human airways from a (**a**) healthy control, (**b**) non-COPD smoker, (**c**) moderate COPD (GOLD II), and (**d**) severe COPD (GOLD IV) were stained with TLR7 using immunohistochemistry. Scale bar = 100μm.

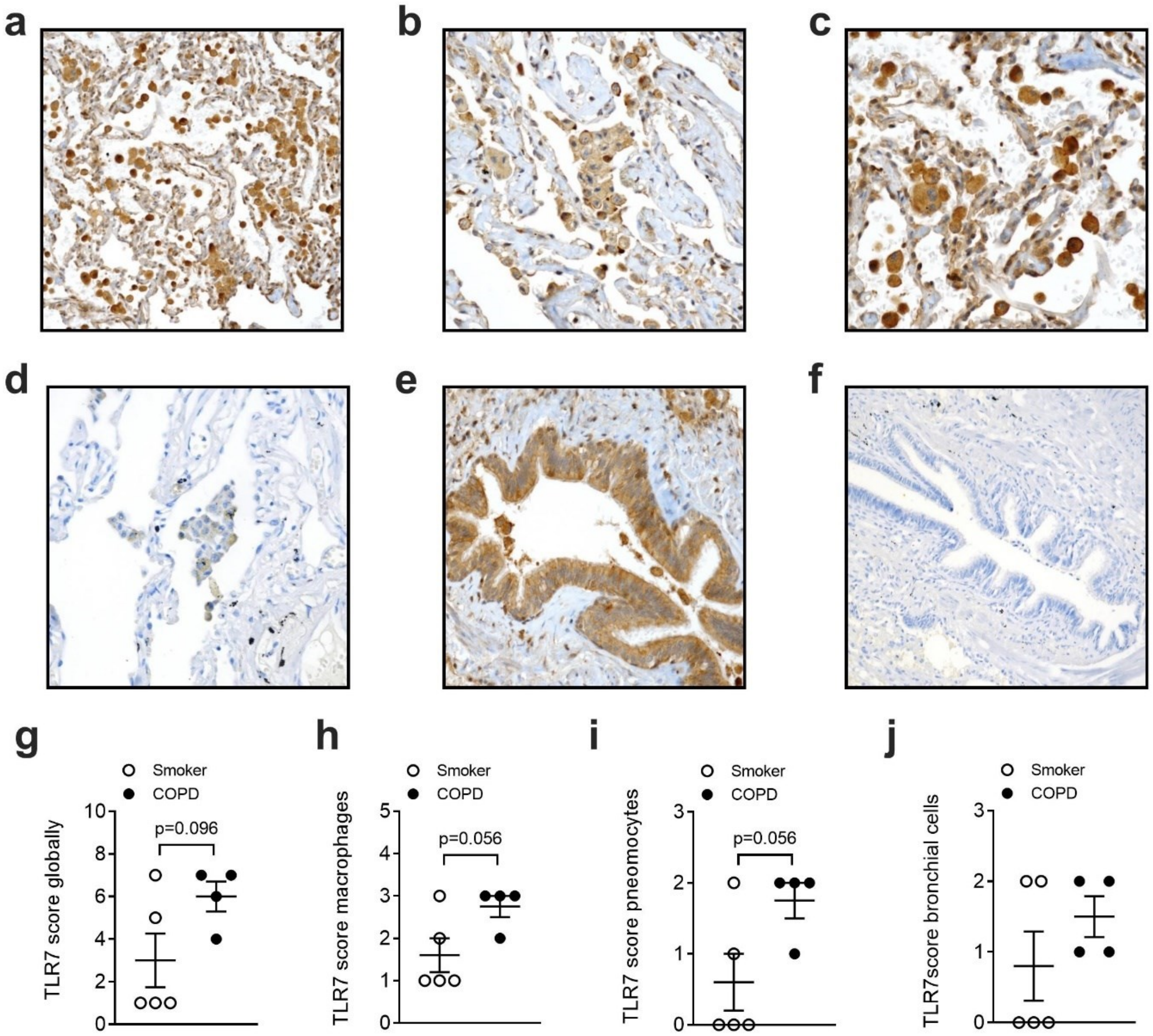

Supplementary Fig. 2 | High levels of TLR7 staining in the membranes and cytoplasm of immune and broncho- and alveolar epithelial cells in peripheral lung tissues of patients with COPD patients compared to smokers with normal lung function. (**a**) Intense and homogeneous TLR7 expression in immune cells and peripheral COPD lung tissue (200X). (**b)** Low TLR7 staining in smokers with normal lung function (400X). (**c**) High TLR7 immunopositivity in alveolar pneumocytes in COPD (400X). (**d**) Negative TLR7 staining in pneumocytes of smokers with normal lung function (400X). (**e**) Evident membranous and cytoplasmic TLR7 expression in broncho-epithelial cells of COPD patients (200X). (**f**) Absence of TLR7 staining in broncho-epithelial cells of smokers with normal lung function (200X). Immunoperoxidase Mayer's haemalum counterstain. Intensity-distribution scores of TLR7 immunostaining were determined in (**g**) lung sections globally, and in (**h**) macrophages, (**i**) pneumocytes, and (**j**) bronchial cells. Scoring was performed by two certified pathologists using a double-headed on a consensus basis. TLR7 score was calculated for each case by adding the values of the area of staining positivity and the intensity of staining. All data are presented as means ± s.e.m. Statistical analysis was performed using two-tailed Mann-Whitney test. Source data are provided as a Source Data file.

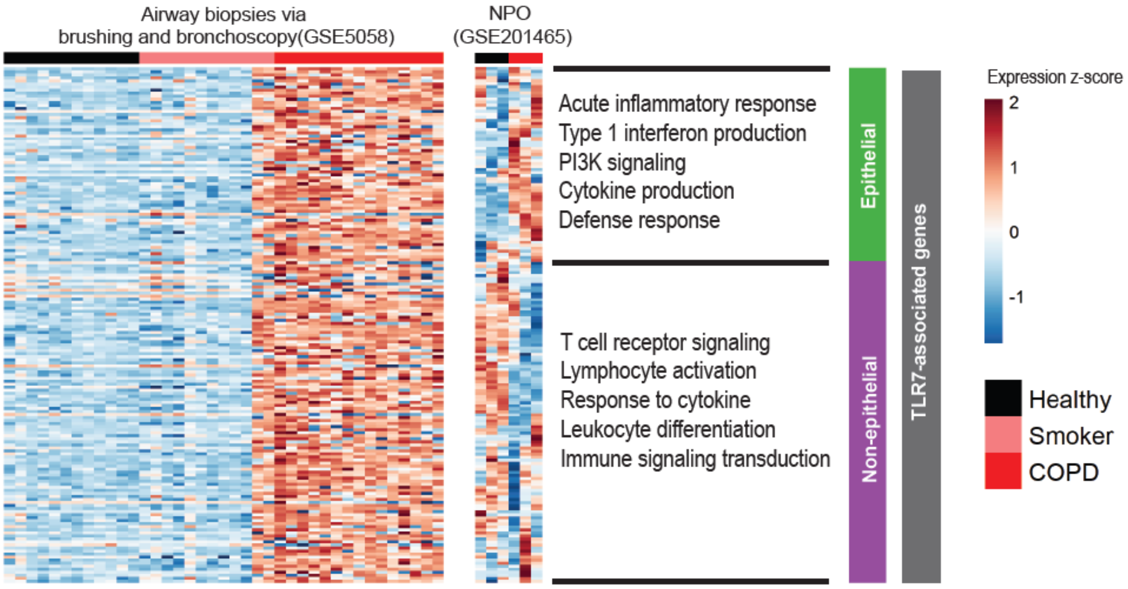

Supplementary Fig. 3 | identification of a TLR gene signature (220 genes) using the transcriptomes of airway biopsies from healthy, smoker and COPD donors (GSE5058). Applying the gene signature to nasopharyngeal organoids (NPOs) revealed epithelial and nonepithelial gene signatures associated with TLR7-mediated inflammatory and lymphocyte activation, respectively.

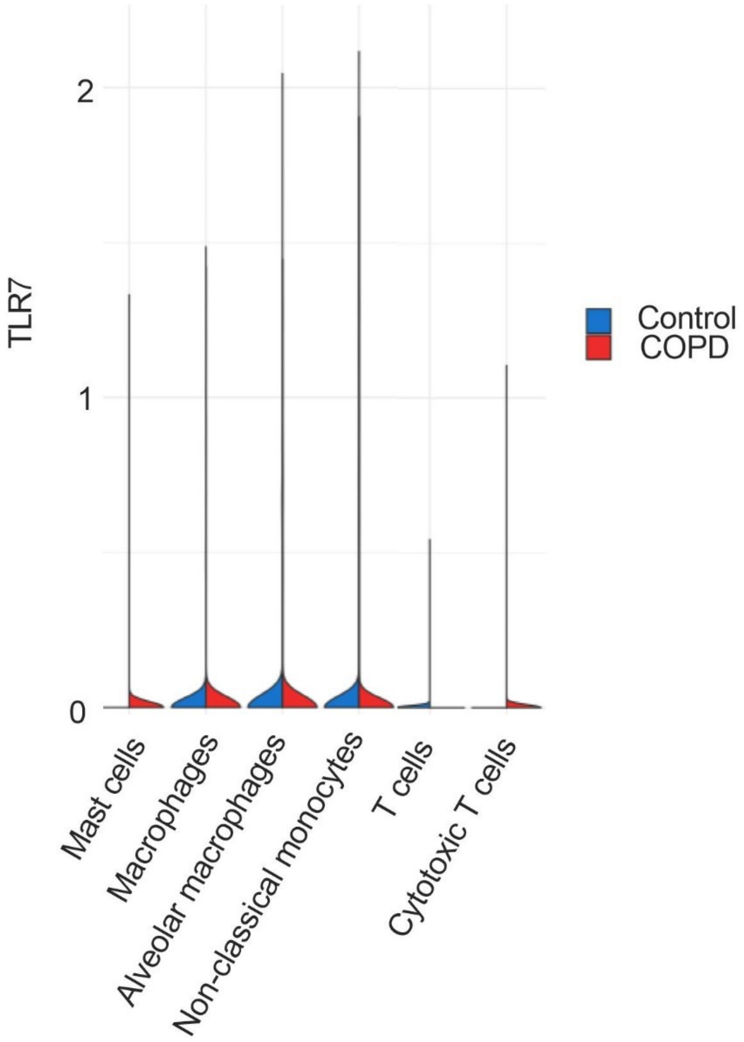

Supplementary Fig. 4 | Immune cells from lung parenchymal tissues of COPD patients and controls produce TLR7 mRNA. Immune cells including mast cells produce *TLR7* mRNA from severe COPD patients (n=17) and lung healthy controls (n=19). The violin plot was generated using COPD Cell Atlas (www.copdcellatlas.com) under gene explorer with selection of immune cells category.

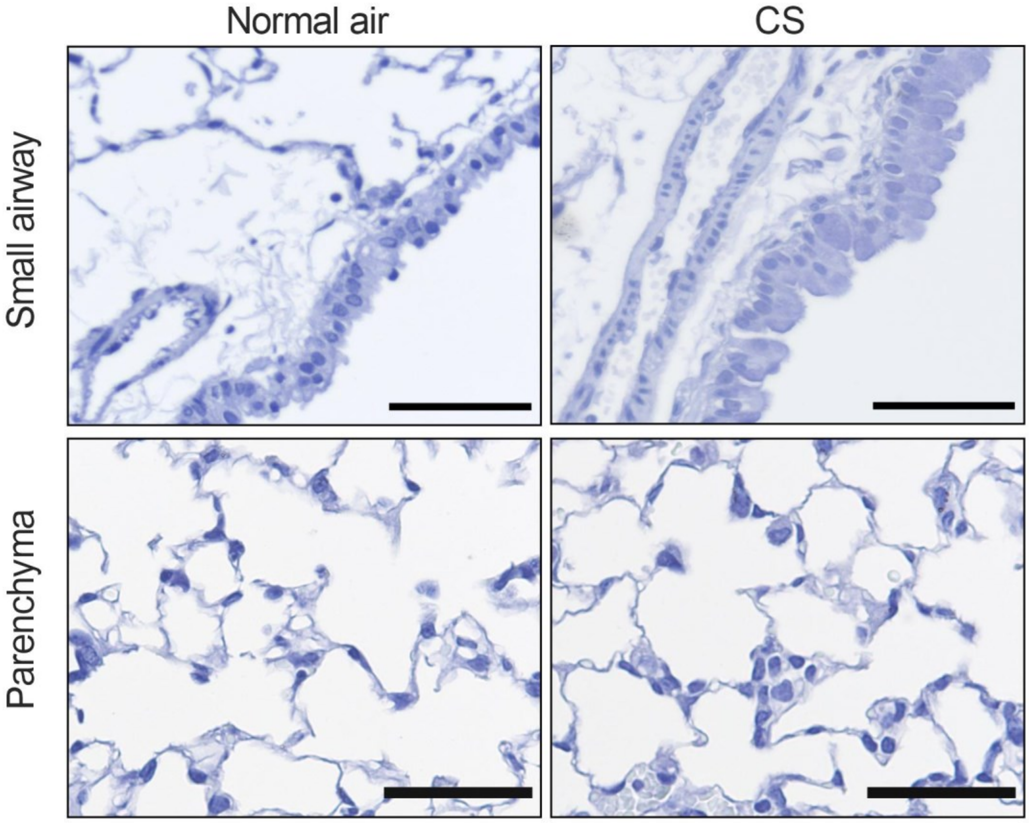

Supplementary Fig. 5 | Non-immune antibody-stained airways and parenchyma in mouse lungs after 8 weeks of cigarette smoke exposure. These images are controls for **Fig. 1o**. Scale bar, 50 µm.

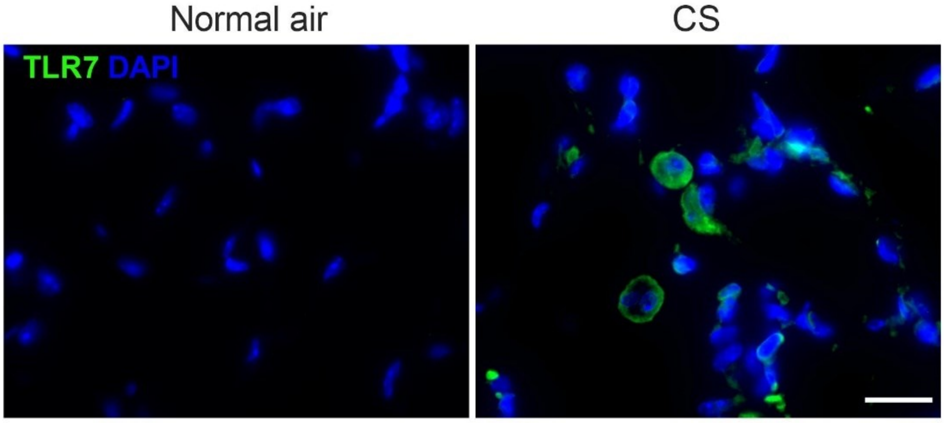

Supplementary Fig. 6 | TLR7$^+$ cells are increased in the lungs in experimental COPD and is located mainly in the cytoplasm. TLR7 (FITC, green) and nuclei (DAPI, blue) were stained in mouse lungs by immunofluorescence (scale bar =50 μm). Representative images for **Fig. 1p**.

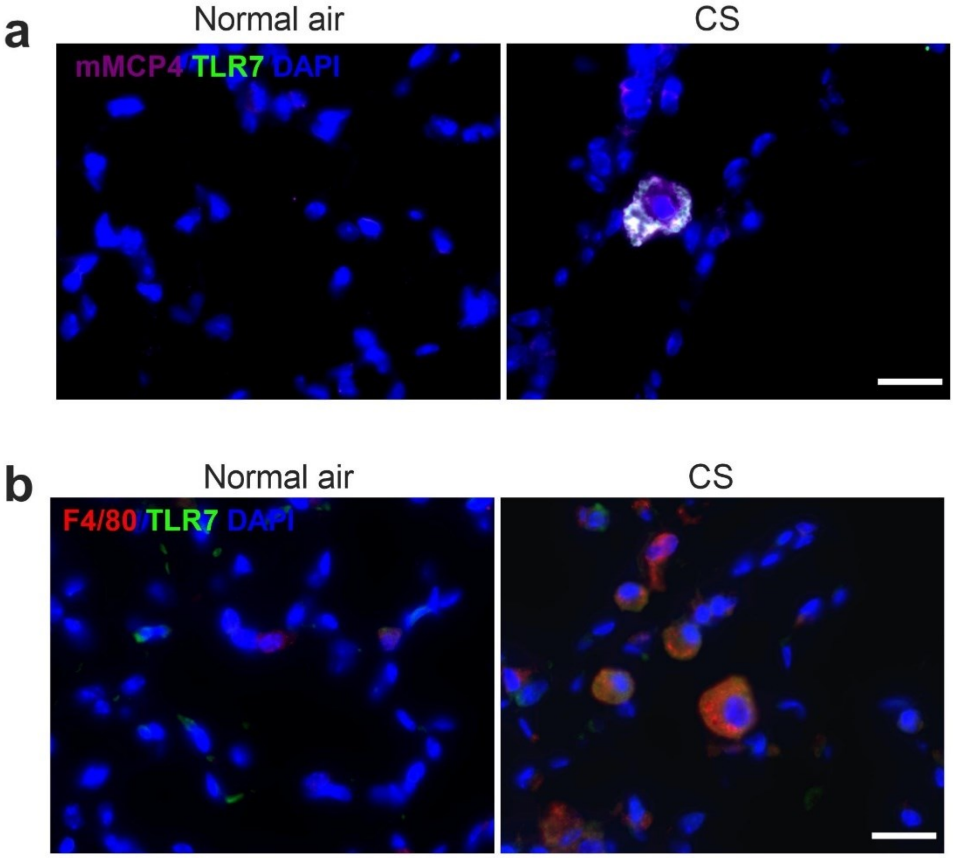

Supplementary Fig. 7 | TLR7$^+$mMCP4$^+$ mast cells and TLR7$^+$F4/80$^+$ macrophages are increased in the lungs in experimental COPD. WT BALB/c mice were exposed to 8 weeks of CS, control mice were exposed to normal air. (**a**) TLR7 (green), mMCP4 (purple) and DAPI (blue) were stained and TLR7$^+$mMCP4$^+$ mast cells, scale bar = 20 μm. Representative images for **Fig. 1q**. (**b**) TLR7 (green), F4/80 (red) and DAPI (blue) were stained in mouse lungs, scale bar = 20 μm. Representative images for **Fig. 1r**.

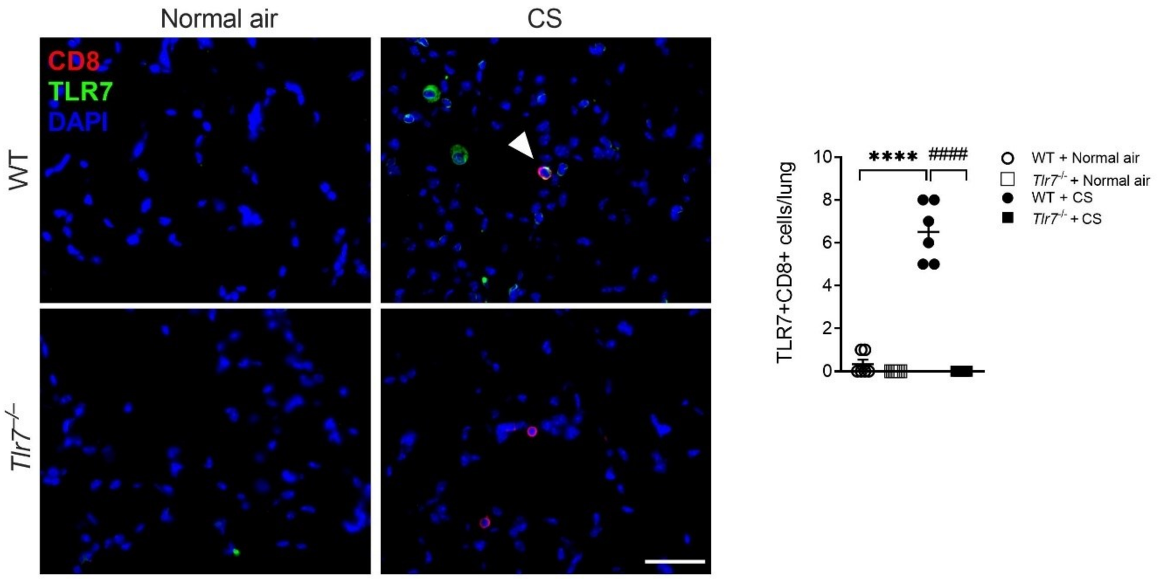

Supplementary Fig. 8 | TLR7^{+}CD8^{+} cells are increased in the lungs of WT but were not detected in *Tlr7*$^{-/-}$ mice with experimental COPD. TLR7 (green), CD8 (red) and nuclei (DAPI, blue) were stained in mouse lungs by immunofluorescence (scale bar = 50 μm), and total TLR7^{+}CD8^{+} cells were enumerated in mouse lung sections. White arrow indicates TLR7^{+}CD8^{+} cells. Results are mean ± s.e.m. ****P<0.05 compared to WT control mice. ####P<0.05 compared to WT CS-exposed mice using one-way ANOVA with Bonferroni's multiple comparison test. Source data are provided as a Source Data file.

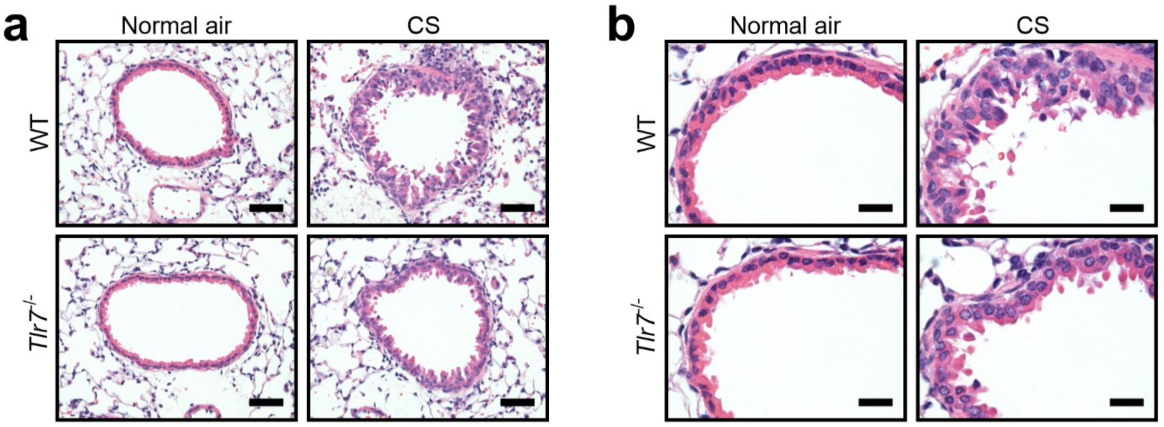

Supplementary Fig. 9 | CS-induced small airway epithelial thickening is reduced in *Tlr7*$^{-/-}$ mice. Wild-type (WT) or *Tlr7*$^{-/-}$ BALB/c mice were exposed to normal air or CS for 8 weeks. (**a**) Representative micrographs of small airways in H&E-stained lung sections ($n = 6$ mice per group) from WT (top panels) and *Tlr7*$^{-/-}$ (bottom panels) mice exposed to normal air (left panels) or CS (right panels) for 8 weeks. Scale bars, 50 μm. (**b**) Representative micrographs showing small airway epithelial cell nuclei in H&E-stained lung sections ($n = 6$ mice per group) from WT (top panels) and *Tlr7*$^{-/-}$ (bottom panels) mice exposed to normal air (left panels) or CS (right panels) for 8 weeks. Scale bars, 20 μm.

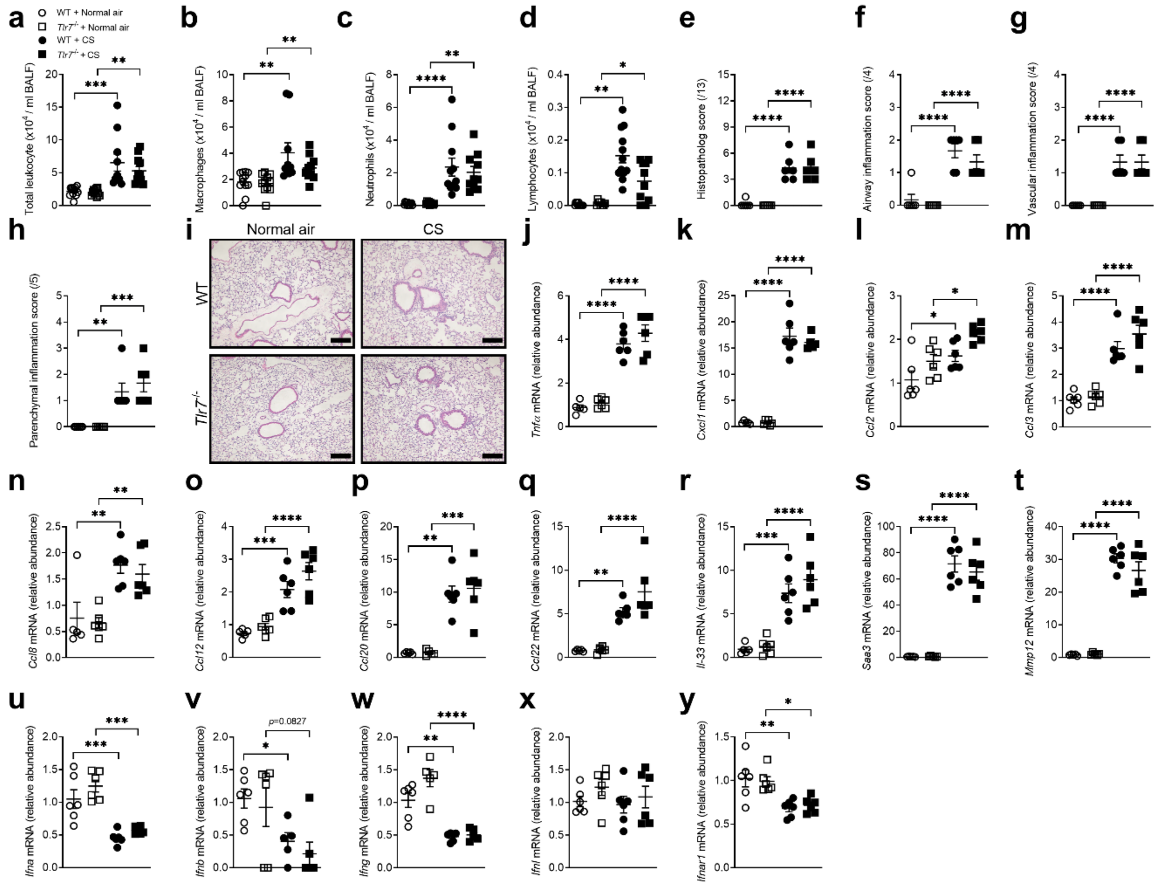

Supplementary Fig. 10 | CS-induced pulmonary inflammation is unaltered in *Tlr7*$^{-/-}$ mice. Wild-type (WT) or *Tlr7*$^{-/-}$ BALB/c mice were exposed to normal air or CS for 8 weeks. (**a**) Total leukocytes, (**b**) macrophages, (**c**) neutrophils and (**d**) lymphocytes in May-Grunwald Giemsa stained BALF cytospins from WT or *Tlr7*$^{-/-}$ mice exposed to normal air- or CS for 8 weeks (*n* = 6 mice per group). (**e** – **h**) Histopathology scores and (**i**) representative micrographs of H&E-stained lung sections (*n* = 6 mice per group) from WT (top panels) and *Tlr7*$^{-/-}$ (bottom panels) mice exposed to normal air (left panels) or CS (right panels) for 8 weeks. Scale bars, 200 µm. (**j**) Tumour necrosis factor (*Tnf*)-α, (**k**) chemokine (C-X-C motif) ligand (*Cxcl*)1, (**l**) chemokine (C-C motif) ligand (*Ccl*)2, (**m**) *Ccl3*, (**n**) *Ccl8*, (**o**) *Ccl12*, (**p**) *Ccl20*, (**q**) *Ccl22*, (**r**) interleukin (*Il*)-33, (**s**) serum amyloid A3 (*Saa3*), (**t**) matrix metalloproteinase (*Mmp*)12, (**u**) interferon alpha (*Ifna*), (**v**) interferon beta (*Ifnb*), (**w**) interferon gamma (*Ifng*), (**x**) interferon lambda (*Ifnl*) and (**y**) interferon receptor 1 (*Ifnar1*) mRNA levels in whole lung homogenates by qPCR from WT or *Tlr7*$^{-/-}$ mice exposed to normal air- or CS for 8 weeks (*n* = 6 mice per group). mRNA data were normalized to the house-keeping *Hprt* transcript and expressed as relative abundance to normal air-exposed WT controls. All data are presented as means ± s.e.m. **P*< 0.05; ***P*< 0.01; ****P*< 0.001; *****P*< 0.0001 compared to normal air-exposed WT or *Tlr7*$^{-/-}$ controls by one-way ANOVA with Bonferonni's multiple comparison test. Source data are provided as a Source Data file.

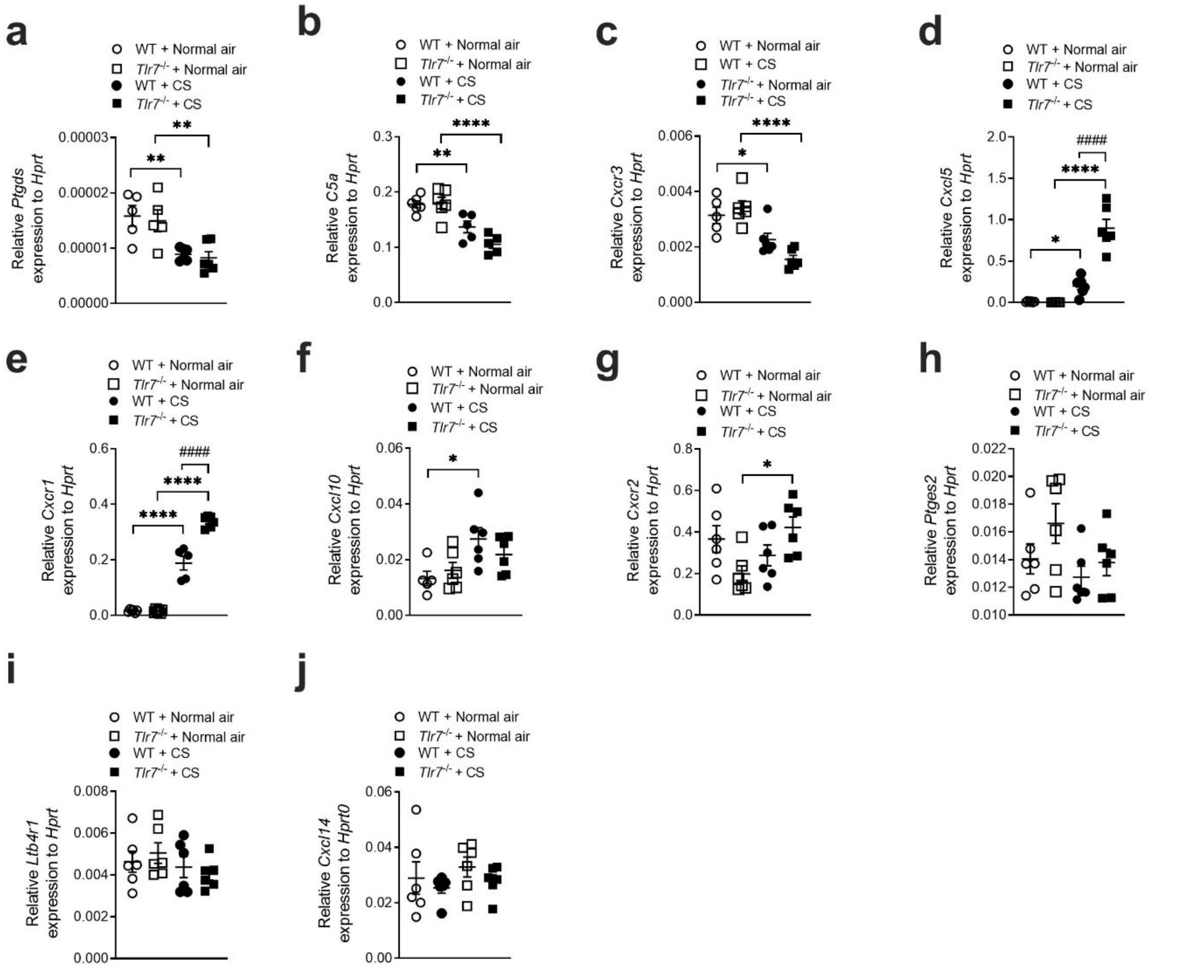

Supplementary Fig. 11 | mRNA expression of mast cell chemokines in WT and $Tlr7^{-/-}$ BALB/c mice after 8 weeks of cigarette smoke (CS) exposure to induce experimental COPD. Mice (n = 6-8) were exposed to 12 cigarettes, 2x/day, 5 days/week for 8 weeks, and controls breathed normal air. mRNA expression of (**a**) *Ptgds*, (**b**) *C5a*, (**c**) *Cxcr3*, (**d**) *Cxcl5*, I *Cxcr1*, (**f**) *Cxcl10*, (**g**) *Cxcr2*, (**h**) *Ptges2*, (**i**) *Ltb4r1* and (**j**) *Cxcl14* mRNA expression were assessed by qPCR. Results are mean ± s.e.m. *P<0.05, **P<0.01, ***P<0.001, ****P<0.0001 compared to control mice. ####P<0.0001 compared to WT mice exposed to CS exposure. Statistical analysis was performed using one-way ANOVA with Bonferroni's multiple comparison test. Source data are provided as a Source Data file.

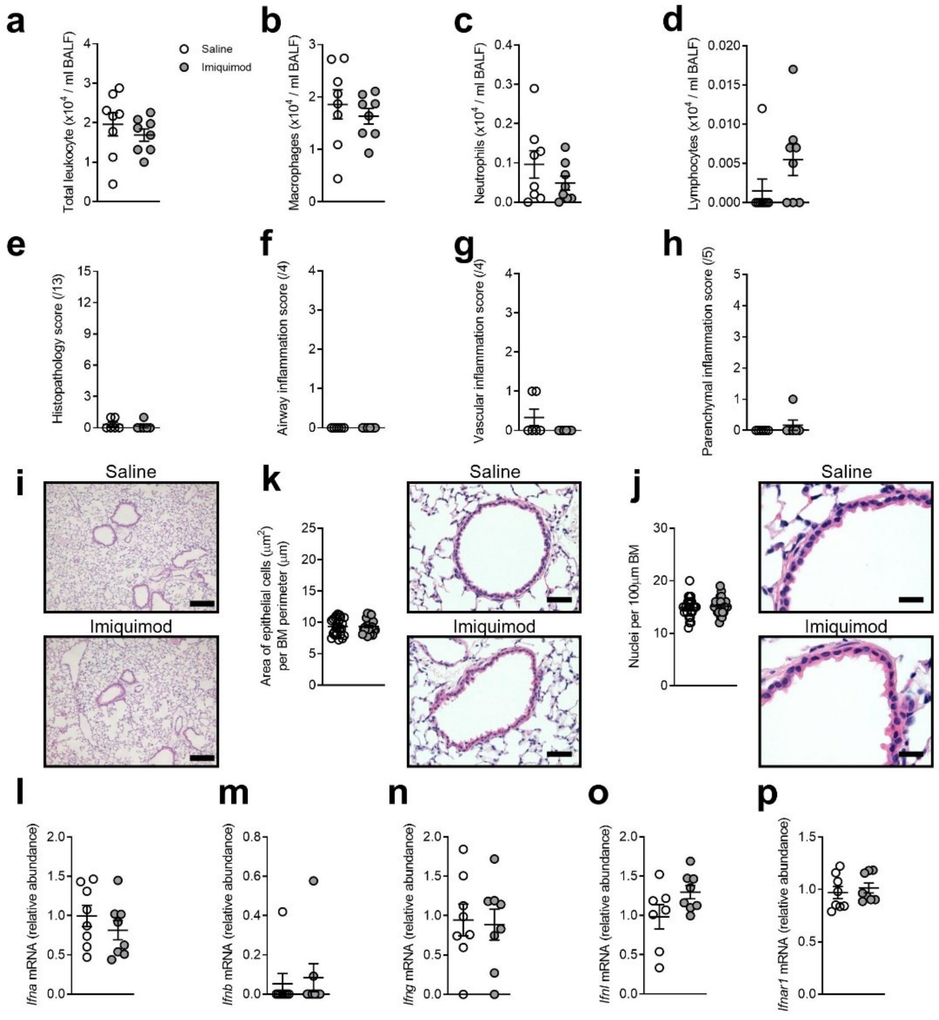

Supplementary Fig. 12 | Administration of imiquimod had no effect on pulmonary inflammation or small airway remodelling in mice. Wild-type (WT) BALB/c mice were administered imiquimod (50 µg) in sterile saline, intranasally 5 times per week, for 8 weeks. Controls received sterile saline. (**a**) Total leukocytes, (**b**) macrophages, (**c**) neutrophils and (**d**) lymphocytes in May-Grunwald Giemsa stained BALF cytospins from WT mice administered saline or imiquimod (n = 8 mice per group). (**e** – **h**) Histopathology scores and (**i**) representative micrographs of small airways in H&E-stained lung sections (n = 6 mice per group) from WT mice administered saline (top panel) and imiquimod (bottom panel). Scale bars, 200 µm. (**j**) Quantification of small airway epithelial cell area per BM perimeter (4 small airways per mouse, n = 6 mice per group) and representative micrographs (right panels) of small airways in H&E-stained lung sections from WT mice administered saline (top panel) and imiquimod (bottom panel). Scale bars, 50 µm. (**k**) Quantification of nuclei numbers per 100 µm of BM perimeter (4 small airways per mouse, n = 6 mice per group) and representative micrographs (right panels) showing small airway epithelial cell nuclei in H&E-stained lung sections from WT mice administered saline (top panel) and imiquimod (bottom panel). Scale bars, 20 µm. (**l**) *Ifna*, (**m**) *Ifnb*, (**n**) *Ifng*, (**o**) *Ifnl* and (**p**) *Ifnar1* mRNA levels in whole lung homogenates by qPCR from WT mice administered saline or imiquimod (n = 8 mice per group). mRNA data were normalized to the house-keeping *Hprt* transcript and expressed as relative abundance to saline-administered WT controls. All data are presented as means ± s.e.m. Statistical analysis was performed using the two-tailed Mann-Whitney test. Source data are provided as a Source Data file.

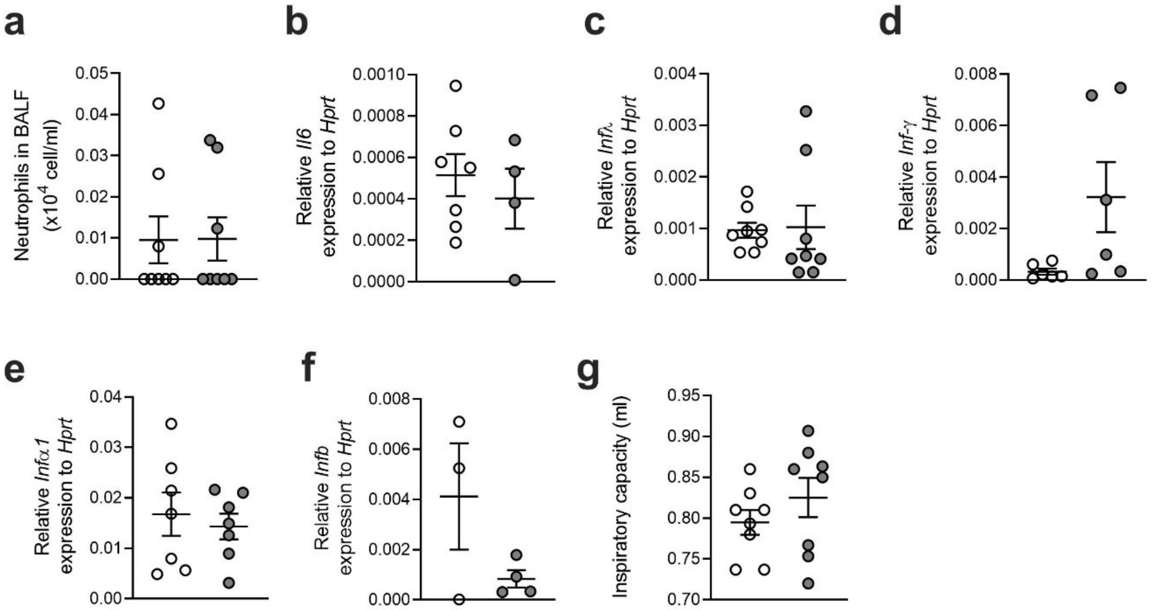

Supplementary Fig. 13 | Lung inflammation in mice after high dose imiquimod challenge. Wild-type BALB/c mice (n = 6-8) were challenged with imiquimod (100 µg in 50 µl sterile saline) intranasally, 5 times per week, for 2 weeks. Controls were challenged with sterile saline. (**a**) Neutrophils in BALF. mRNA expression of (**b**) *Il6*, (**c**) *Infg*, (**d**) *Infl*, (**e**) *Infar1,* (**f**) *Infb* were assess in mouse lungs by qPCR. (**g**) Lung function, in terms of inspiratory capacity was assessed using flexiVent apparatus. Results are mean ± s.e.m. Statistical analysis was performed using the two-tailed Mann-Whitney test. Source data are provided as a Source Data file.

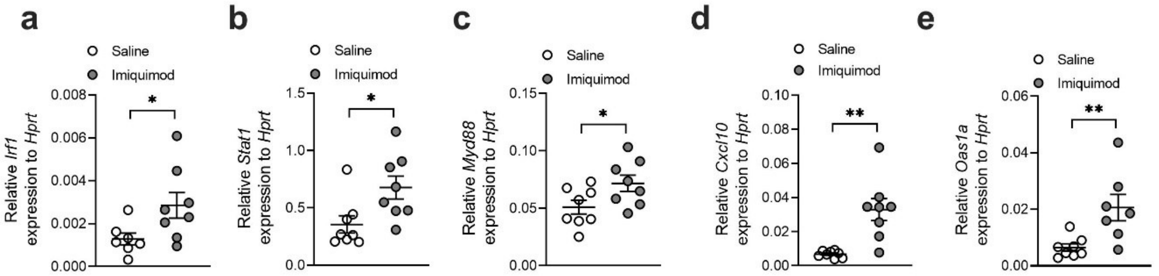

Supplementary Fig. 14 | High dose imiquimod challenge increases the mRNA expression of TLR7 downstream molecules in mouse lungs. WT BALB/c mice (n=6-8) were challenged with high-dose imiquimod (100 µg in 50 µl sterile saline) intranasally, 5 times per week, for 2 weeks. Controls received sterile saline. RNA was extracted from mouse lungs and converted to cDNA by RT-PCR. (**a**) *Ifr1*, (**b**) *Stat1*, (**c**) *Myd88*, (**d**) *Cxcl10*, and (**e**) *Oas1a* mRNA expressions were assessed by qPCR. Results are mean ± SEM. *P<0.05, **P<0.01, compared to controls using the two-tailed Mann-Whitney test. Source data are provided as a Source Data file.

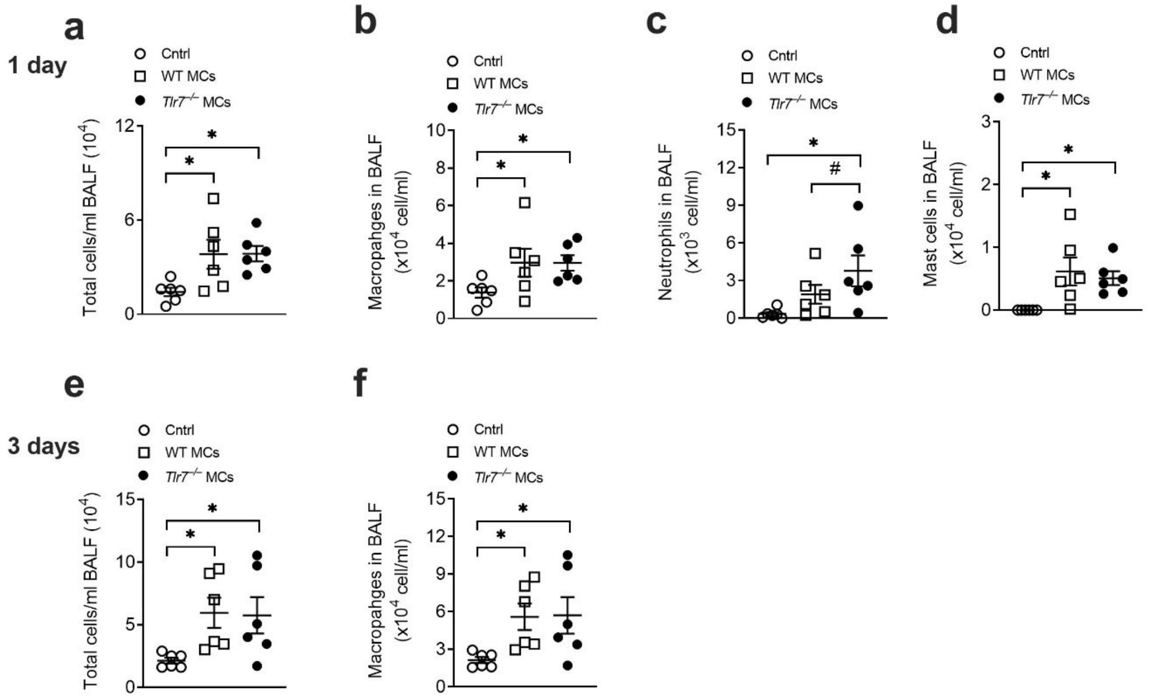

Supplementary Fig. 15 | Bone marrow-derived *Tlr7*$^{-/-}$ mast cells induce less airway inflammation than WT mast cells. WT BALB/c mice were administered 5x10^5 bone-marrow-derived mast cells intranasally from either WT or *Tlr7*$^{-/-}$ mice. (**a**) Totally leukocytes, (**b**) macrophages, (**c**) neutrophils and (**d**) mast cells in bronchoalveolar lavage fluid (BALF) 1 day after receiving mast cells. (**e**) Totally leukocytes and (**f**) macrophages in BALF 3 days after receiving mast cells. n = 8. Results are mean ± s.e.m. *P<0.05 compared to control mice. #P<0.05, ##P<0.01, compared to mice administered mast cells from WT mice. Statistical differences were determined with one-way ANOVA followed by Bonferroni post-test. Source data are provided as a Source Data file.

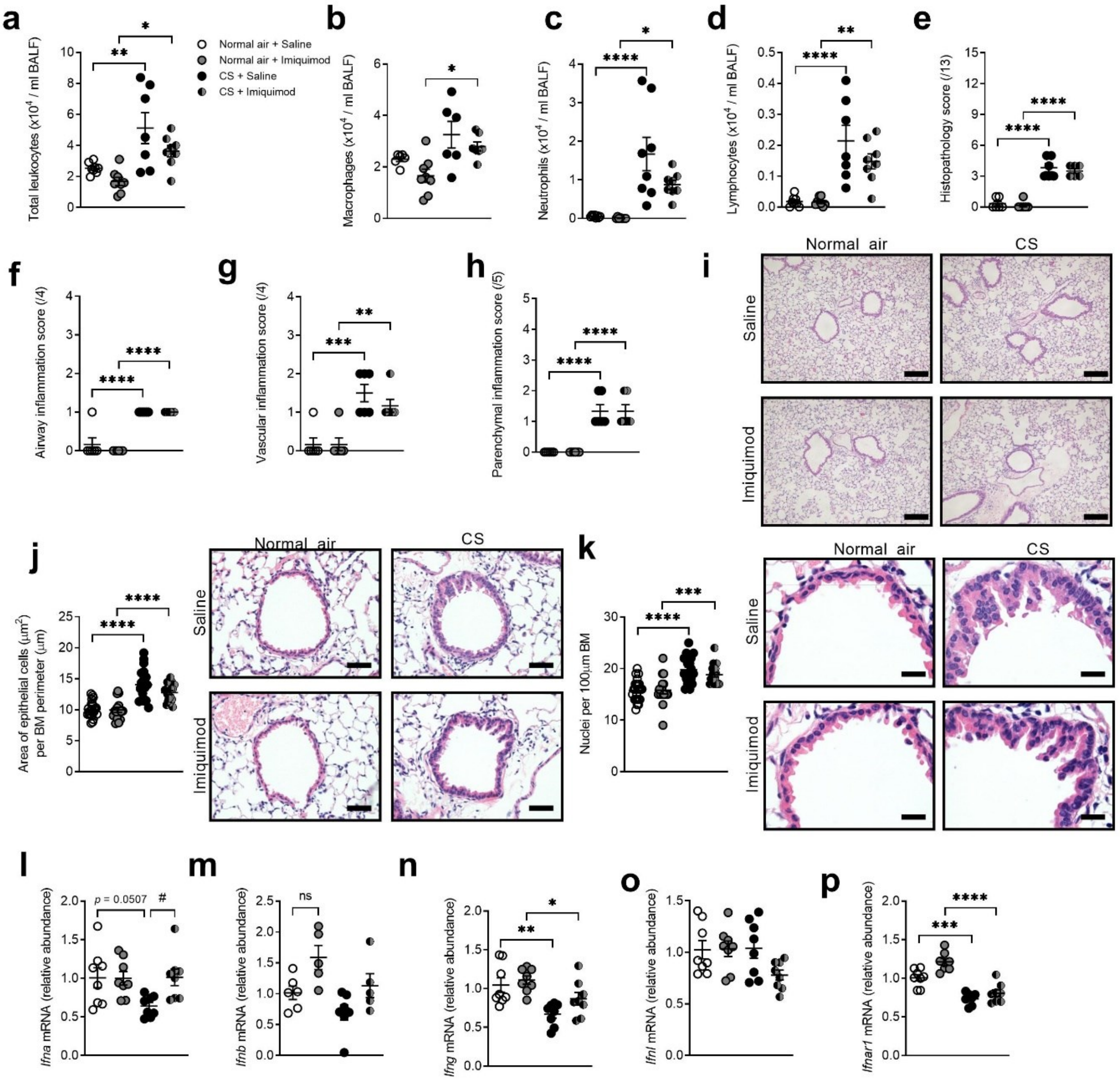

Supplementary Fig. 16 | Administration of imiquimod during chronic CS exposure has minimal effects on pulmonary inflammation or small airway remodelling in experimental COPD. Wild-type (WT) BALB/c mice were exposed to normal air or CS for 8 weeks and were administered imiquimod (50 µg) in sterile saline intranasally 5 times per week, between week 6 to 8 (for 2 weeks). Controls received sterile saline. (**a**) Total leukocytes, (**b**) macrophages, (**c**) neutrophils and (**d**) lymphocytes in May-Grunwald Giemsa stained BALF cytospins from saline- and imiquimod-administered WT mice exposed to normal air or CS for 8 weeks (*n* = 8 mice per group). (**e** – **h**) Histopathology scores and (**i**) representative micrographs of H&E-stained lung sections (*n* = 6 mice per group) from saline (top panels)- and imiquimod (bottom panels)-administered WT mice exposed to normal air (left panels) or CS (right panels) for 8 weeks. Scale bars, 200 µm. (**j**) Quantification of small airway epithelial cell area per µm of basement membrane (BM) perimeter (4 small airways per mouse, *n* = 6 mice per group) and representative micrographs (right panels) of small airways in H&E-stained lung sections from saline (top panels)- and imiquimod (bottom panels)-administered WT mice exposed to normal air (left panels) or CS (right panels) for 8 weeks. Scale bars, 50 µm. (**k**) Quantification of nuclei numbers per 100 µm of BM perimeter (4 small airways per mouse, *n* = 6 mice per group) and representative micrographs (right panels) showing small airway epithelial cell nuclei in H&E-stained lung sections from saline (top

panels)- and imiquimod (bottom panels)-administered WT mice exposed to normal air (left panels) or CS (right panels) for 8 weeks. Scale bars, 20 µm. (**l**) *Ifna*, (**m**) *Ifnb*, (**n**) *Ifng*, (**o**) *Ifnl* and (**p**) *Ifnar1* mRNA levels in whole lung homogenates from saline- and imiquimod-administered WT mice exposed to normal air or CS for 8 weeks (n = 8 mice per group). mRNA data were normalized to the house-keeping *Hprt* transcript and expressed as relative abundance to saline-administered WT controls. All data are presented as means ± s.e.m. *P< 0.05; **P< 0.01; ***P< 0.001; ****P< 0.0001 compared to saline- or imiquimod-administered WT mice exposed to normal air, and $^{\#}P$< 0.05 compared to imiquimod-administered WT mice exposed to CS by one-way ANOVA with Bonferonni's multiple comparison test; ns, not significant. Source data are provided as a Source Data file.

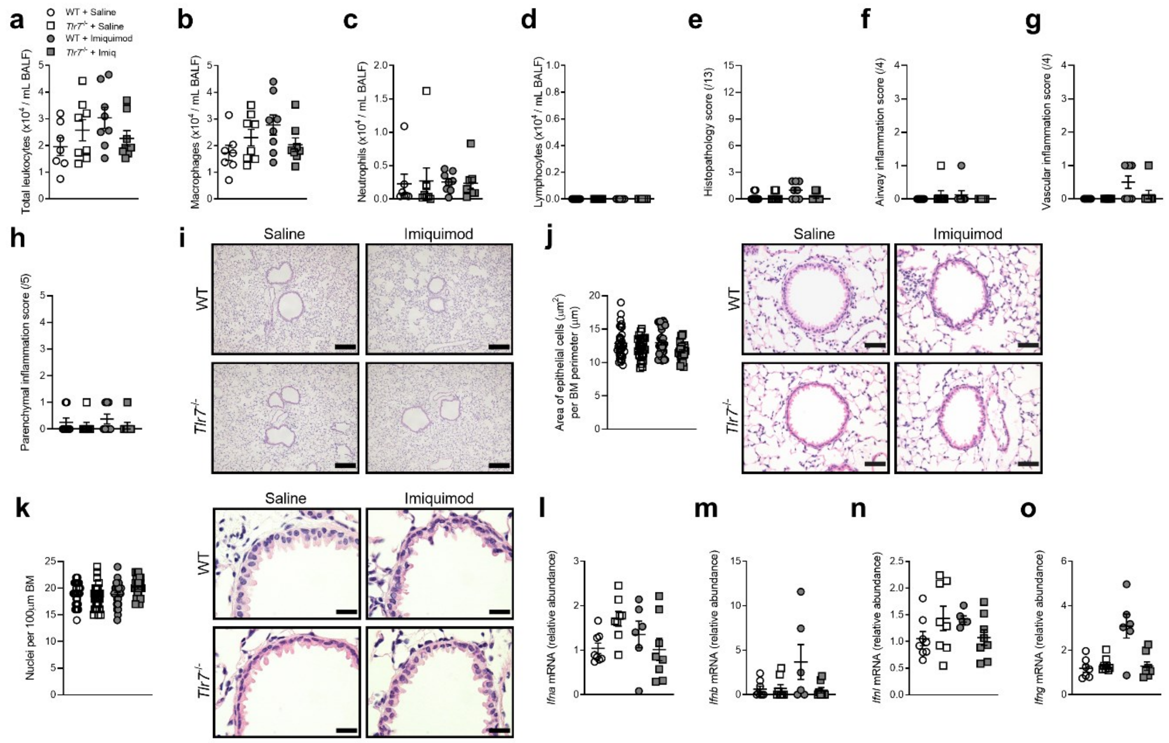

Supplementary Fig. 17 | Administration of imiquimod does not affect pulmonary inflammation or small airway remodelling in *Tlr7*$^{-/-}$ mice. Wild-type (WT) or *Tlr7*$^{-/-}$ BALB/c mice were administered imiquimod (50 µg) in sterile saline, intranasally 5 times per week, for 2 weeks. Controls received sterile saline. (**a**) Total leukocytes, (**b**) macrophages, (**c**) neutrophils and (**d**) lymphocytes in May-Grunwald Giemsa stained BALF cytospins from WT and *Tlr7*$^{-/-}$ mice administered saline or imiquimod (*n* = 8 mice per group). (**e – h**) Histopathology scores and (**i**) representative micrographs of H&E-stained lung sections (*n* = 8 mice per group) from WT (top panels) and *Tlr7*$^{-/-}$ (bottom panels) mice administered saline (left panels) or imiquimod (right panels). Scale bars, 200 µm. (**j**) Quantification of small airway epithelial cell area per µm of basement membrane (BM) perimeter (4 small airways per mouse, *n* = 8 mice per group) and representative micrographs (right panels) of small airways in H&E-stained lung sections from WT (top) and *Tlr7*$^{-/-}$ (bottom panels) mice administered saline (left panels) or imiquimod (right panels). Scale bars, 50 µm. (**k**) Quantification of nuclei numbers per 100 µm of BM perimeter (4 small airways per mouse, *n* = 8 mice per group) and representative micrographs (right panels) showing small airway epithelial cell nuclei in H&E-stained lung sections from WT (top panels) and *Tlr7*$^{-/-}$ (bottom panels) mice administered saline (left panels) or imiquimod (right panels). Scale bars, 20 µm. (**l**) *Ifna*, (**m**) *Ifnb*, (**n**) *Ifng* and (**o**) *Ifnl* mRNA levels in whole lung homogenates from saline- and imiquimod-administered WT or *Tllr7*$^{-/-}$ mice exposed to normal air or CS for 8 weeks (*n* = 8 mice per group). mRNA data were normalized to the house-keeping *Hprt* transcript and expressed as relative abundance to saline-administered WT controls. All data are presented as means ± s.e.m. Statistical analysis was performed using one-way ANOVA with Bonferroni's multiple comparison test. Source data are provided as a Source Data file.

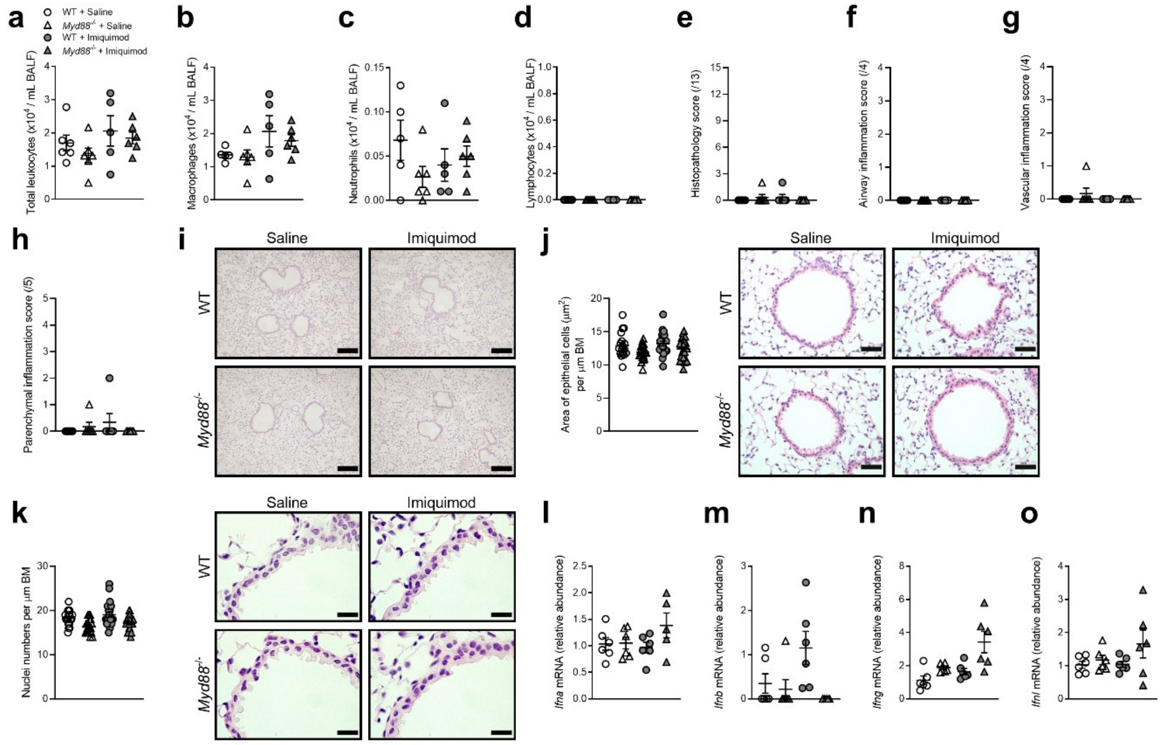

Supplementary Fig. 18 | Administration of imiquimod does not affect pulmonary inflammation or small airway remodelling in *Myd88*$^{-/-}$ mice. Wild-type (WT) or *Myd88*$^{-/-}$ BALB/c mice were administered imiquimod (50 µg) in sterile saline, intranasally 5 times per week, for 2 weeks. Controls received sterile saline. (**a**) Total leukocytes, (**b**) macrophages, (**c**) neutrophils and (**d**) lymphocytes in May-Grunwald Giemsa stained BALF cytospins from WT and *Myd88*$^{-/-}$ mice administered saline or imiquimod (*n* = 6 mice per group). (**e – h**) Histopathology scores and (**i**) representative micrographs of H&E-stained lung sections (*n* = 6 mice per group) from WT (top panels) and *Myd88*$^{-/-}$ (bottom panels) mice administered saline (left panels) or imiquimod (right panels). Scale bars, 200 µm. (**j**) Quantification of small airway epithelial cell area per µm of BM perimeter (4 small airways per mouse, *n* = 6 mice per group) and representative micrographs (right panels) of small airways in H&E-stained lung sections from WT (top panels) and *Myd88*$^{-/-}$ (bottom panels) mice administered saline (left panels) or imiquimod (right panels). Scale bars, 50 µm. (**k**) Quantification of nuclei numbers per 100 µm of BM perimeter (4 small airways per mouse, *n* = 6 mice per group) and representative micrographs (right panels) showing small airway epithelial cell nuclei in H&E-stained lung sections from WT (top panels) and *Myd88*$^{-/-}$ (bottom panels) mice administered saline (left panels) or imiquimod (right panels). Scale bars, 20 µm. (**l**) *Ifna*, (**m**) *Ifnb*, (**n**) *Ifng* and (**o**) *Ifnl* mRNA levels in whole lung homogenates from saline- and imiquimod-administered WT or *Myd88*$^{-/-}$ mice (*n* = 6 mice per group). mRNA data were normalized to the house-keeping *Hprt* transcript and expressed as relative abundance to saline-administered WT controls. Throughout, data are presented as means ± s.e.m. Statistical analysis was performed using one-way ANOVA with Bonferroni's multiple comparison test. Source data are provided as a Source Data file.

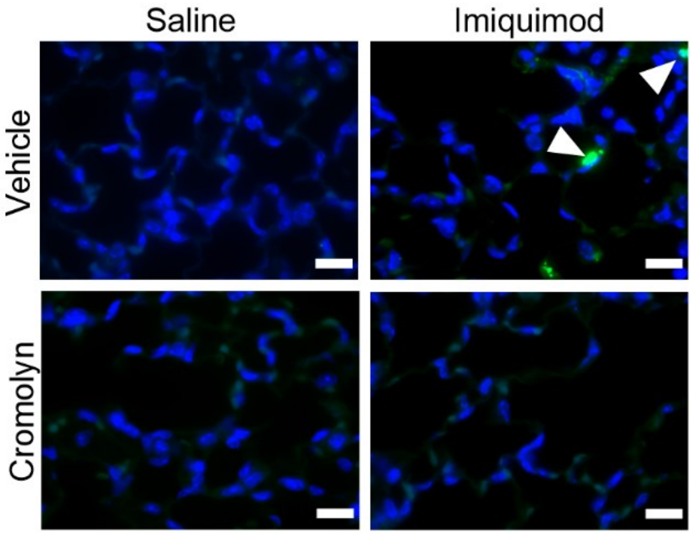

Supplementary Fig. 19 | Mast cell stabilizer cromolyn reduces imiquimod-induced apoptosis. Wild-type BALB/c mice were first treated with vehicle (sterile water) or cromolyn (50 mg/kg body weight) 2 hours prior to administration of sterile saline or imiquimod (50 µg), intranasally 5 times per week, for 2 weeks. Representative micrographs of TUNEL-stained lung sections (*n* = 8 mice per group) from vehicle- (top panels) or cromolyn (bottom panels)-treated mice administered saline (left panels) or imiquimod (right panels). Arrows indicate $TUNEL^+$ cells. Scale bars, 20 µm.

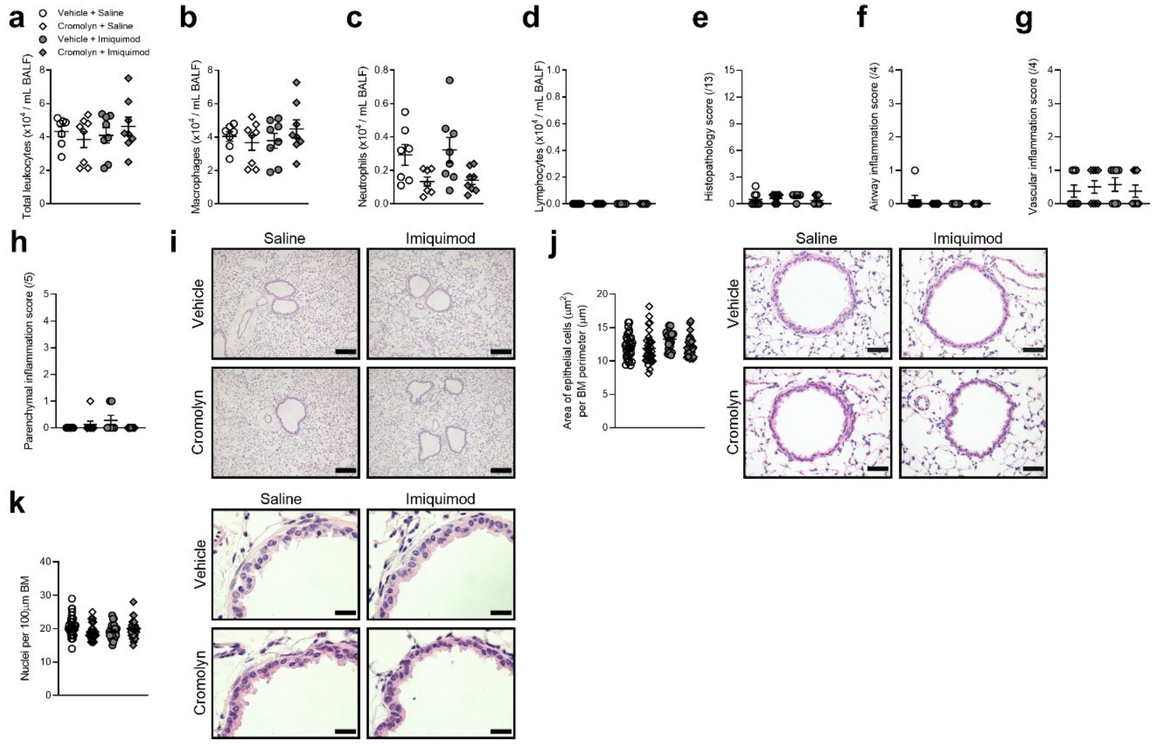

Supplementary Fig. 20 | Administration of mast cell stabilizer cromolyn with or without imiquimod does not affect pulmonary inflammation or small airway remodelling. Wild-type BALB/c mice were first treated with vehicle (sterile water) or cromolyn (50 mg/kg body weight) 2 hours prior to administration of sterile saline or imiquimod (50 µg), intranasally 5 times per week, for 2 weeks. (**a**) Total leukocytes, (**b**) macrophages, (**c**) neutrophils and (**d**) lymphocytes in May-Grunwald Giemsa stained BALF cytospins from vehicle- or cromolyn-treated mice administered saline or imiquimod (*n* = 8 mice per group). (**e – h**) Histopathology scores and (**i**) representative micrographs of H&E-stained lung sections (*n* = 6 mice per group) from vehicle- (top panels) and cromolyn-treated (bottom panels) mice administered saline (left panels) or imiquimod (right panels). Scale bars, 200 µm. (**j**) Quantification of small airway epithelial cell area per µm of BM perimeter (4 small airways per mouse, *n* = 8 mice per group) and representative micrographs (right panels) of small airways in H&E-stained lung sections from vehicle- (top panels) and cromolyn-treated (bottom panels) mice administered saline (left panels) or imiquimod (right panels). Scale bars, 50 µm. (**k**) Quantification of nuclei numbers per 100 µm of BM perimeter (4 small airways per mouse, *n* = 6 mice per group) and representative micrographs (right panels) showing small airway epithelial cell nuclei in H&E-stained lung sections from vehicle- (top panels) and cromolyn-treated (bottom panels) mice administered saline (left panels) or imiquimod (right panels). Scale bars, 20 µm. Throughout, data are presented as means ± s.e.m. Statistical analysis was performed using one-way ANOVA with Bonferroni's multiple comparison test. Source data are provided as a Source Data file.

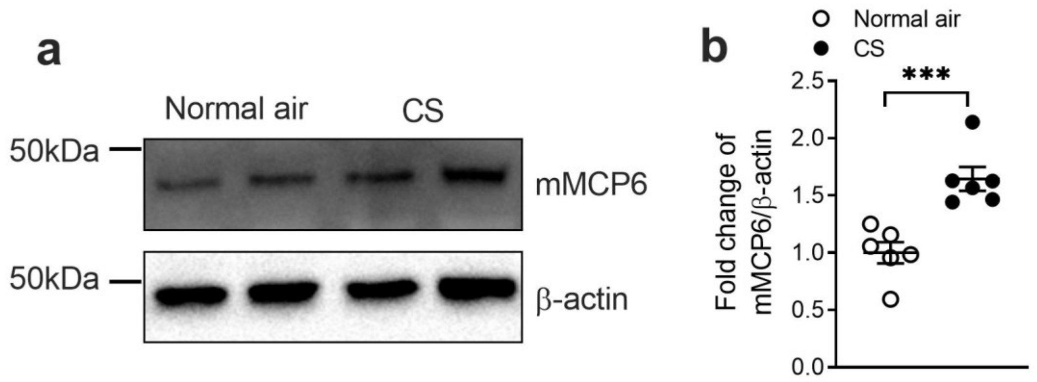

Supplementary Fig. 21 | mMCP6 protein increased in mouse lungs after 8 weeks cigarette smoke (CS) exposure. Wild-type BALB/c mice were received 8 weeks CS exposure or normal air. (**a**) mMCP6 protein were measured in mouse lungs by immunoblot, and was (**b**) quantitated by densitometry analysis of fold change normalised to β-actin. Results are mean ± s.e.m. ***P<0.001 compared to control mice using two-tailed Mann-Whitney test. n = 6 mice per group. Source data are provided as a Source Data file.

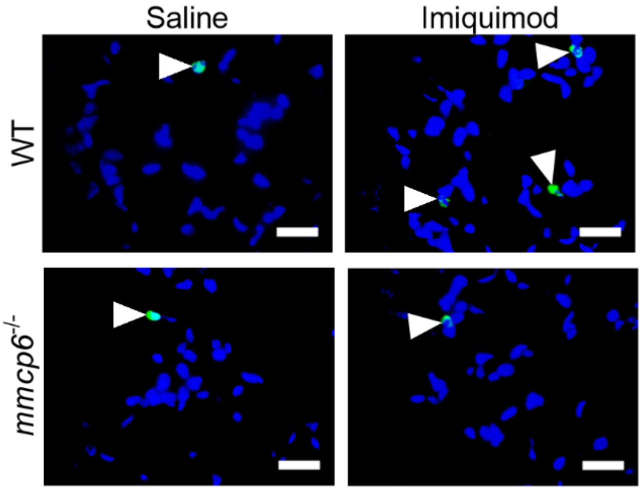

Supplementary Fig. 22 | Imiquimod-induced apoptosis is reduced in *mmcp6*$^{-/-}$ mice. Wild-type or *mmcp6*$^{-/-}$ C57BL/6 mice were administered imiquimod (50 µg) in sterile saline, intranasally 5 times per week, for 2 weeks. Controls received sterile saline. Representative micrographs of TUNEL-stained lung sections (n = 6 mice per group) from WT (top panels) or *mmcp6*$^{-/-}$ (bottom panels) mice administered saline (left panels) or imiquimod (right panels). Arrows indicate TUNEL^{+} cells. Scale bars, 20 µm.

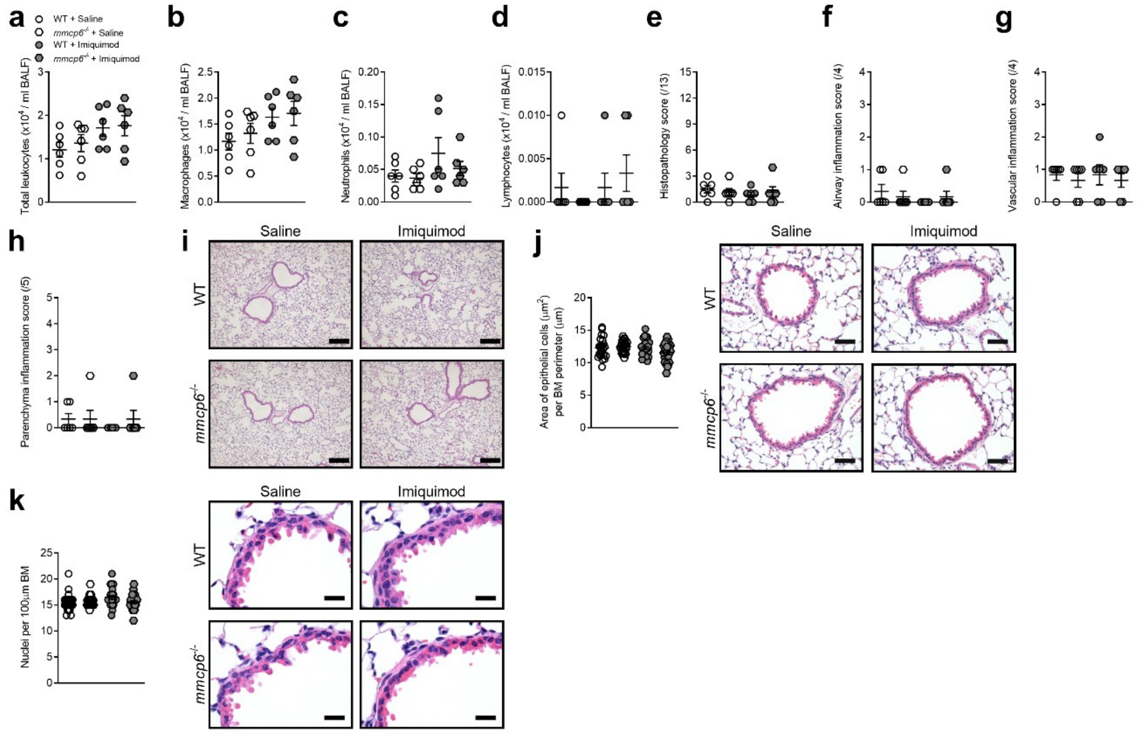

Supplementary Fig. 23| Administration of imiquimod does not affect pulmonary inflammation or small airway remodelling in *mMCP6*$^{-/-}$ mice. Wild-type (WT) or *mMCP6*$^{-/-}$ C57BL/6 mice were administered imiquimod (50 µg) in sterile saline, intranasally 5 times per week, for 2 weeks. Controls received sterile saline. (**a**) Total leukocytes, (**b**) macrophages, (**c**) neutrophils and (**d**) lymphocytes in May-Grunwald Giemsa stained BALF cytospins from WT and *mMCP6*$^{-/-}$ mice administered saline or imiquimod (*n* = 6 mice per group). (**e–h**) Histopathology scores and (**i**) representative micrographs of H&E-stained lung sections (*n* = 6 mice per group) from WT (top panels) and *mMCP6*$^{-/-}$ (bottom panels) mice administered saline (left panels) or imiquimod (right panels). Scale bars, 200 µm. (**j**) Quantification of small airway epithelial cell area per µm of BM perimeter (4 small airways per mouse, *n* = 6 mice per group) and representative micrographs (right panels) of small airways in H&E-stained lung sections from WT (top panels) and *mMCP6*$^{-/-}$ (bottom panels) mice administered saline (left panels) or imiquimod (right panels). Scale bars, 50 µm. (**k**) Quantification of nuclei numbers per 100 µm of BM perimeter (4 small airways per mouse, *n* = 6 mice per group) and representative micrographs (right panels) showing small airway epithelial cell nuclei in H&E-stained lung sections from WT (top panels) and *mMCP6*$^{-/-}$ (bottom panels) mice administered saline (left panels) or imiquimod (right panels). Scale bars, 20 µm. Throughout, data are presented as means ± s.e.m. Statistical analysis was performed using one-way ANOVA with Bonferroni's multiple comparison test. Source data are provided as a Source Data file.

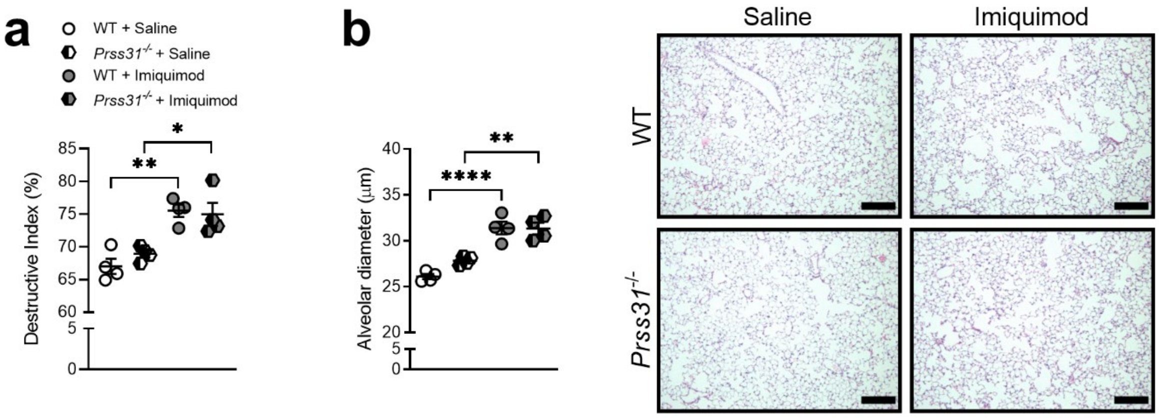

Supplementary Fig. 24 | Imiquimod-induced emphysema is not altered in *Prss31*$^{-/-}$ mice. Wild-type or *Prss31*$^{-/-}$ C57BL/6 mice were administered imiquimod (50 µg) in sterile saline, intranasally 5 times per week, for 2 weeks. Controls received sterile saline. (**a**) Quantification of destructive index (n = 4 mice per group) of saline- or imiquimod-administered WT and *Prss31*$^{-/-}$ mice. (**b**) Quantification of mean linear intercept (n = 4 mice per group) and representative micrographs (right) of H&E-stained lung sections from WT (top panels) and *Prss31*$^{-/-}$ (bottom panels) mice administered saline (left panels) or imiquimod (right panels). Scale bars, 200 µm. Throughout, data are presented as means ± s.e.m. *$P< 0.05$; **$P< 0.01$; ****$P< 0.0001$ compared to saline-administered WT or *Prss31*$^{-/-}$ mice by one-way ANOVA with Bonferonni's multiple comparison test. Source data are provided as a Source Data file.

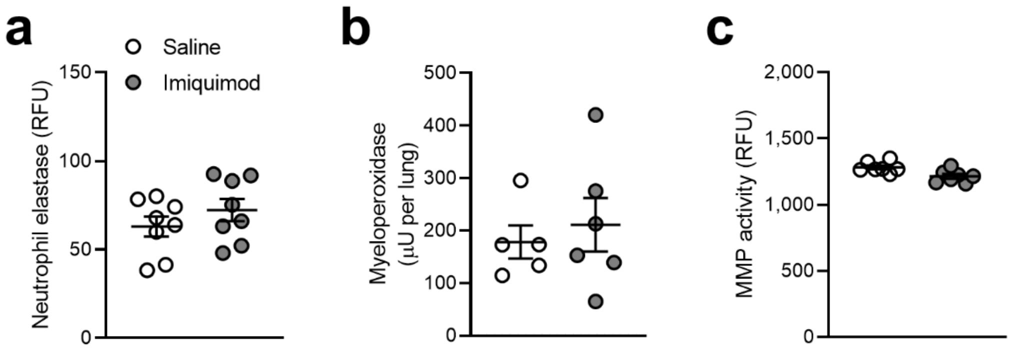

Supplementary Fig. 25 | Administration of imiquimod does not affect other known proteases in the lung. Wild-type C57BL/6 mice were administered imiquimod (50 µg) in sterile saline, intranasally 5 times per week for 8 weeks. Controls received sterile saline. Quantification of (**a**) neutrophil elastase, (**b**) myeloperoxidase and (**c**) total matrix metalloproteinase (MMP) activities in the lungs of WT mice administered saline or imiquimod (n = 6 mice per group). Throughout, data are presented as means ± s.e.m. Statistical analysis was performed using the two-tailed Mann-Whitney test. Source data are provided as a Source Data file.

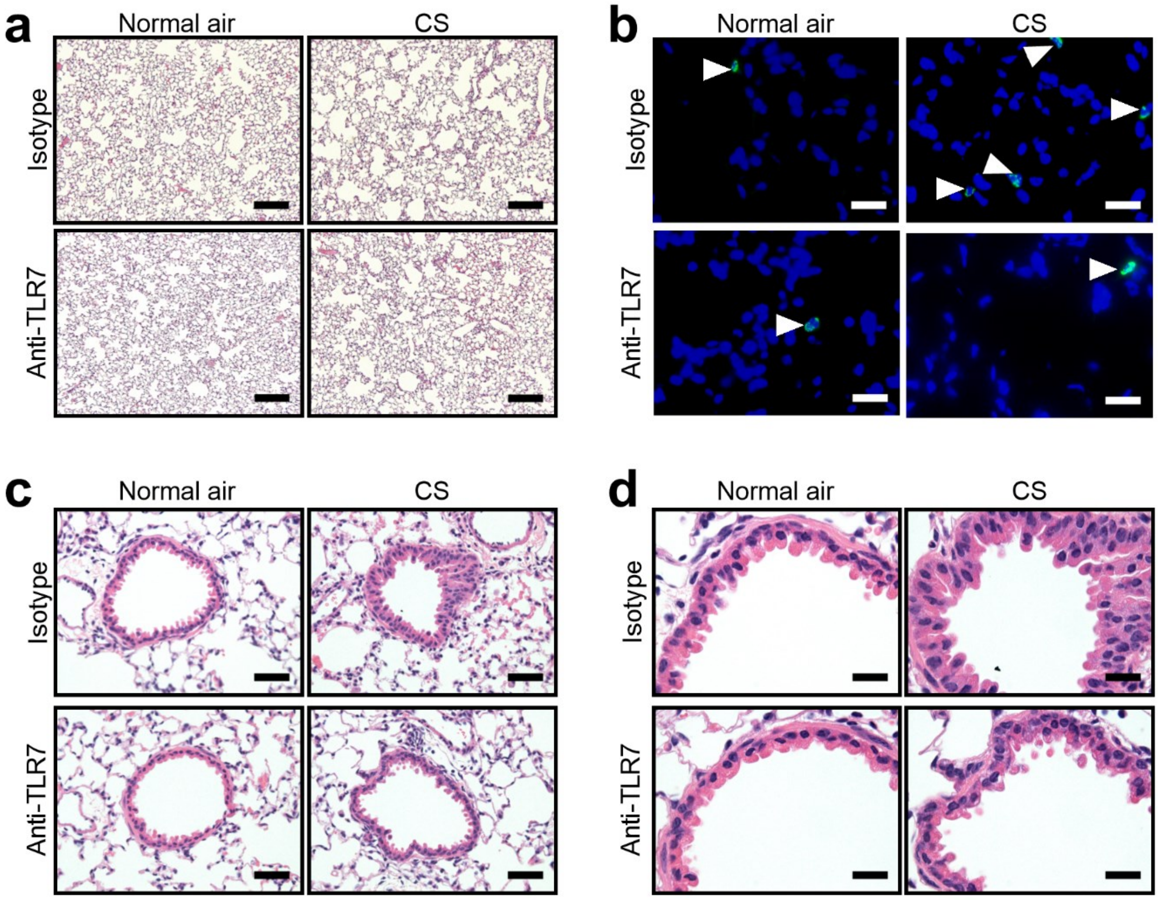

Supplementary Fig. 26| Neutralization of TLR7 reduces emphysema and small airway remodelling in experimental COPD. Wild-type BALB/c mice were exposed to normal air or CS for 8 weeks and administered neutralizing anti-TLR7 monoclonal antibody or isotype control, intravenously (i.v.) once per week, between Week 6 to 8 (for 2 weeks). (**a**) Representative micrographs (*n* = 6 mice per group) of H&E-stained lung sections from isotype (top panels)- or anti-TLR7 (bottom panels)-treated WT mice exposed to normal air (left panels) or CS (right panels) for 8 weeks. Scale bars, 200 µm. (**b**) Representative micrographs (*n* = 6 mice per group) of TUNEL-stained lung sections from isotype (top panels)- or anti-TLR7 (bottom panels)-treated WT mice exposed to normal air (left panels) or CS (right panels) for 8 weeks. Arrows indicate $TUNEL^+$ cells. Scale bars, 20 µm. Representative micrographs (*n* = 6 mice per group) of (**c**) small airways and (**d**) small airway epithelial cell nuclei in H&E-stained lung sections from isotype (top panels)- or anti-TLR7 (bottom panels)-treated WT mice exposed to normal air (left panels) or CS (right panels) for 8 weeks. Scale bars, 20 µm.

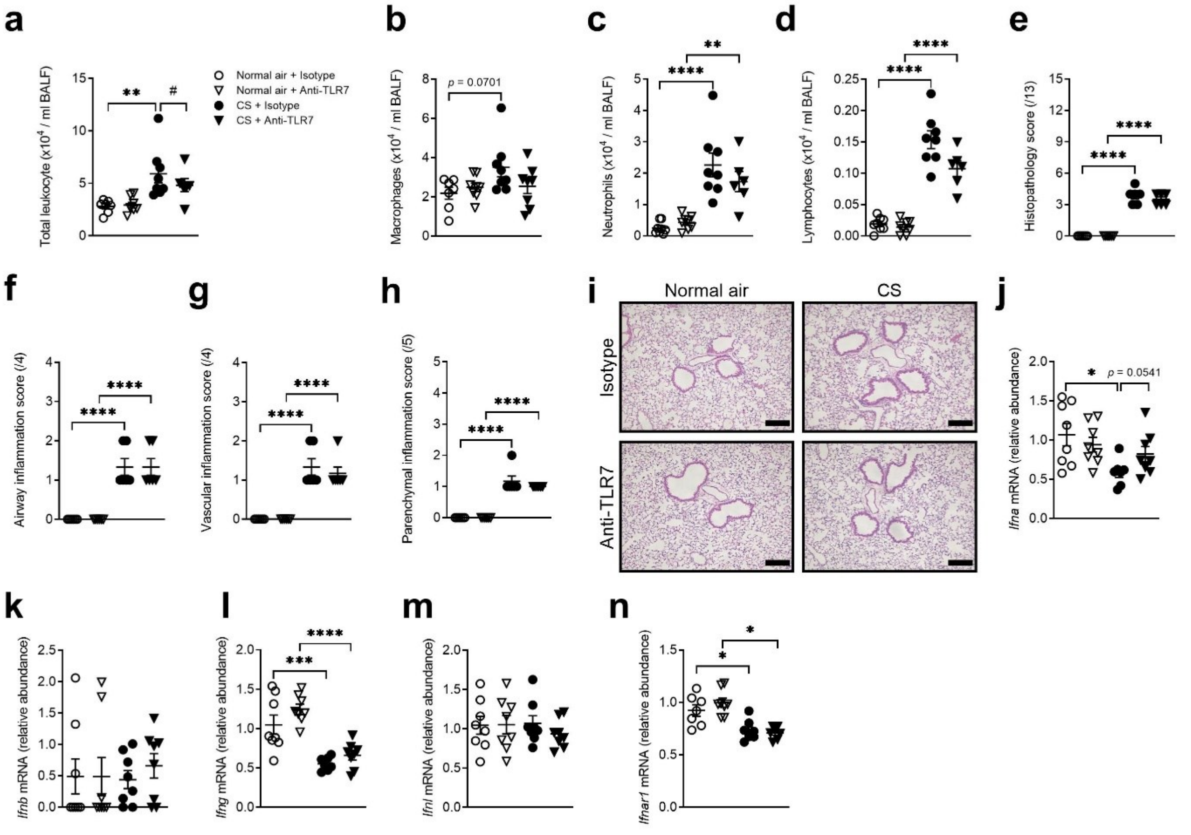

Supplementary Fig. 27| Neutralization of TLR7 has modest effects on pulmonary inflammation in experimental COPD. Wild-type (WT) BALB/c mice were exposed to normal air or CS for 8 weeks and administered neutralizing anti-TLR7 monoclonal antibody or isotype control, intravenously (i.v.) once per week, between Week 6 to 8 (for 2 weeks). (**a**) Total leukocytes, (**b**) macrophages, (**c**) neutrophils and (**d**) lymphocytes in May-Grunwald Giemsa stained BALF cytospins from isotype- and anti-TLR7-treated WT mice exposed to normal air or CS for 8 weeks (n = 8 mice per group). (**e – h**) Histopathology scores and (**i**) representative micrographs of H&E-stained lung sections (n = 8 mice per group) from isotype (top panels)- and anti-TLR7 (bottom panels)-treated WT mice exposed to normal air (left panels) or CS (right panels). Scale bars, 200 µm. (**j**) *Ifna*, (**k**) *Ifnb*, (**l**) *Ifng* (**m**) *Ifnl* and (**n**) *Ifnar1* mRNA levels in whole lung homogenates by qPCR from isotype- and anti-TLR7-treated WT mice exposed to normal air or CS for 8 weeks (n = 8 mice per group). mRNA data were normalized to house-keeping *Hprt* transcript and expressed as relative abundance to saline-administered WT controls. Throughout, data are presented as means ± s.e.m. **P*< 0.05; ***P*< 0.01; ****P*< 0.001; *****P*< 0.0001 compared to isotype- or anti-TLR7-administered WT mice exposed to normal air, and ####*P*< 0.0001 compared to anti-TLR7-administered WT mice exposed to CS by one-way ANOVA with Bonferonni's multiple comparison test. Source data are provided as a Source Data file.

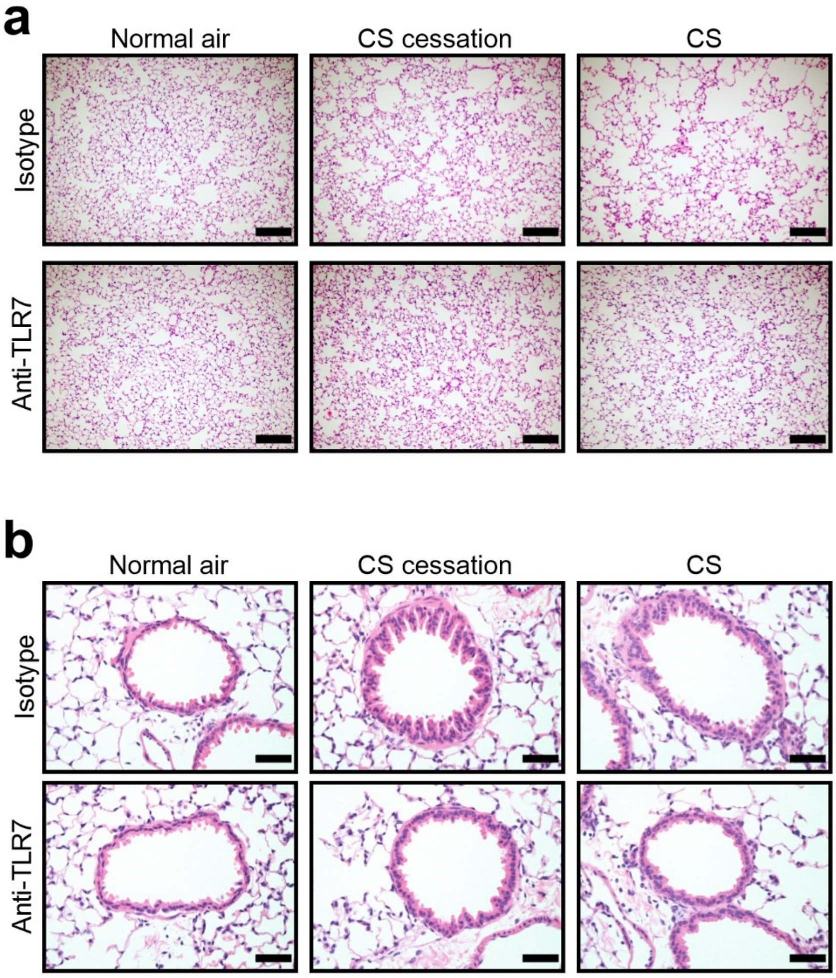

Supplementary Fig. 28| Neutralization of TLR7 during disease progression in a 12-week experimental COPD model reduces emphysema and small airway remodelling. Wild-type (WT) BALB/c mice were exposed to normal air or CS for 12 weeks and some groups underwent CS cessation after 8 week of CS exposure. Mice were treated with either neutralizing anti-TLR7 monoclonal antibody or isotype control, intravenously once per week, between Week 8 to 12 (for 4 weeks). (**a**) Representative micrographs (*n* = 5-6 mice per group) of H&E-stained lung sections from isotype (top panels)- and anti-TLR7 (bottom panels)-treated WT mice exposed to normal air (left panels) or CS (right panels) for 12 weeks and those with CS cessation (middle panels). Scale bars, 200 µm. (**b**) Representative micrographs (4 small airways per mouse, *n* = 5-6 mice per group) of small airways in H&E-stained lung sections from isotype (top panels)- and anti-TLR7 (bottom panels)-treated WT mice exposed to normal air (left panels) or CS (right panels) for 12 weeks and those with CS cessation (middle panels). Scale bars, 50 µm.

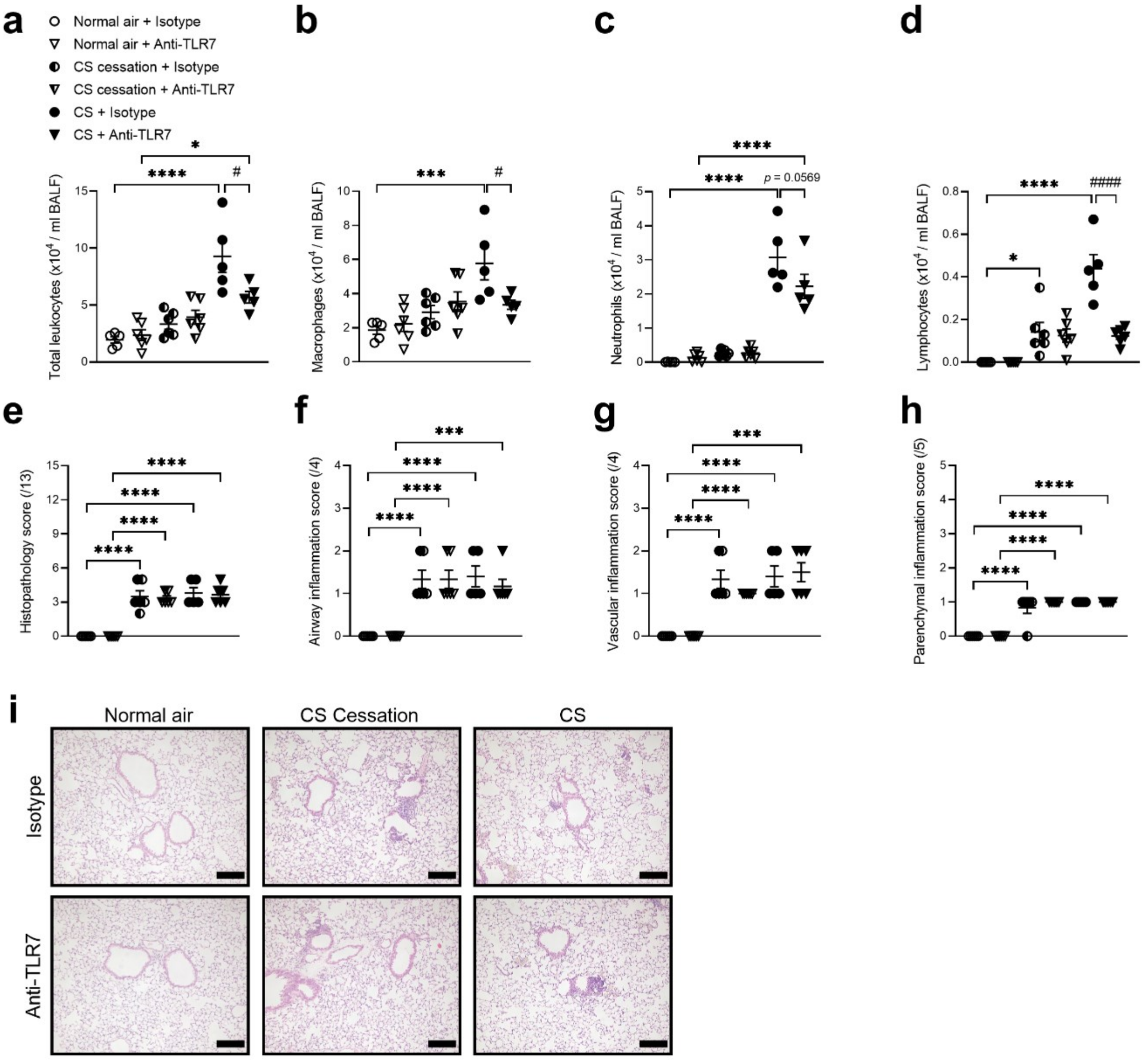

Supplementary Fig. 29| Neutralization of TLR7 during disease progression in a 12-week experimental COPD model reduced inflammation. Wild-type BALB/c mice were exposed to normal air or CS for 12 weeks and some groups underwent CS cessation after 8 week of CS exposure. Mice were treated with either neutralizing anti-TLR7 monoclonal antibody or isotype control, intravenously once per week, between Week 8 to 12 (for 4 weeks). (**a**) Total leukocytes, (**b**) macrophages, (**c**) neutrophils and (**d**) lymphocytes in May-Grunwald Giemsa stained BALF cytospins from isotype- and anti-TLR7-administered WT mice exposed to normal air or CS for 12 weeks and with or with CS cessation (*n* = 6 mice per group). (**e – h**) Histopathology scores and (**i**) representative micrographs of H&E-stained lung sections (*n* = 6 mice per group) from isotype (top panels)- and anti-TLR7 (bottom panels)-administered WT mice exposed to normal air (left panels) or CS (right panels) and with or with CS cessation (middle panels). Throughout, data are presented as means ± s.e.m. **P*< 0.05; ***P*< 0.01; ****P*< 0.001; *****P*< 0.0001 compared to isotype- or anti-TLR7-treated WT mice exposed to normal air, and #*P*< 0.05; ####*P*< 0.0001 compared to anti-TLR7-treated WT mice exposed to CS for 12 weeks or those with CS cessation by one-way ANOVA with Bonferonni's multiple comparison test. Source data are provided as a Source Data file.

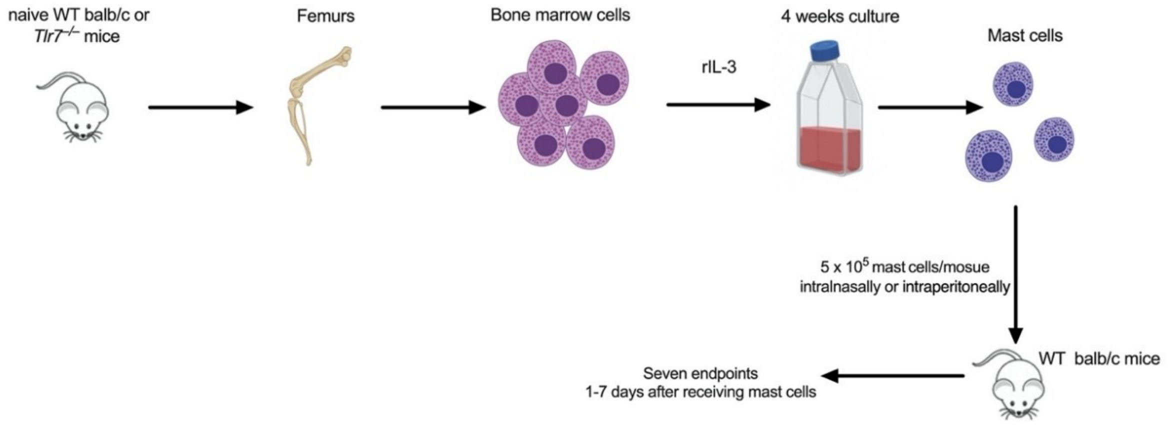

Supplementary Fig. 30| Bone marrow-derived mast cell transfers.

SUPPLEMETNARY TABLES

Supplementary Table 1 | Demographic and clinical data of donors for microarray study (GSE5058)[1]

Parameter	Normal nonsmokers	Normal smokers	Smokers with established COPD*	*P* Value
n	20	15	13	
Sex (male/female), n	16/4	12/3	12/1	>0.5
Age, yr	43 ± 8	46 ± 6	52 ± 6	<0.02†
Smoking history, pack-yr	0	33 ± 18	42 ± 27	>0.3
FVC	106 ± 10	104 ± 13	97 ± 18	>0.1
FEV1	106 ± 11	106 ± 16	77 ± 24	<0.0001†
FEV1/FVC	82 ± 6	81 ± 3	64 ± 12	<0.0001†
TLC	98 ± 10	96 ± 12	107 ± 20	>0.09
DL_{CO}	95 ± 10	95 ± 10	75 ± 17	<0.0001†
Gold stage 0/I/II/III, n*	—	—	2/4/6/1	

Abbreviations: DL_{CO} = diffusing lung capacity for carbon monoxide;
Values are mean ± SD unless otherwise indicated.
*Smokers with established COPD were defined using GOLD criteria.
†*P* value significant for COPD *versus* nonsmoker and COPD versus smoker, but not for smoker *versus* nonsmoker.

Supplementary Table 2 | Demographic and clinical data of donors for microarray study (GSE8545)

Patient ID	Description	Sex	Age	Pack years	Smoking status	Lm mean ± SD (µm)	Lm range (µm)
6965	COPD	M	62	50	Former	716 ± 164	494-982
6967	COPD	F	61	25	Former	414 ± 82	334-585
6968	COPD	F	63	38	Former	724 ± 252	357-1,013
6969	COPD[a,b]	F	56	54	Former	1,822 ± 1270	521-4,620
6970	COPD[c]	M	55	15	Former	1,352 ± 599	647-2,551
6971	COPD	M	59	30	Former	1,097 ± 441	720-2,101
6982	Donor	M	59	-	Never	384 ± 47	344-473
6983	Donor	M	62	24	Former	289 ± 41	231-352

Participants with COPD had FEV_1/FVC <70% and FEV_1 <25% predicted. [a-c]Some patients had other diseases: [a]von Willebrand disease; [b]hypertension; [c]α1-antitrypsin deficiency.

Supplementary Table 3 | Differential *TLR7* gene expression analysis of donors by GOLD status adjusted for age, sex and smoking status

Gene name	GOLDII - NS logFC	GOLDII - NS P.Value	GOLDII - NS adj.P.Val	GOLDII - smoker logFC	GOLDII - smoker P.Value	GOLDII - smoker adj.P.Val
TLR7 (222952_s_at)	1.481563	0.002464	0.019798	1.089945	0.013927	0.155532

Statistical analysis was performed using unpaired student t- test.

Supplementary Table 4 | Patient characteristics for TLR7 immunohistochemistry (n = 4)

	**Never smoker**	**Smoker without COPD**	**COPD GOLD II**	**COPD GOLD III-IV**
**Number**	1	1	1	1
**Age (years)**	58	60	76	57
**Sex**	male	female	female	female
**Smoking**				
Current/ex-smoker	NA	current	current	ex
Pack-years	NA	40	45	-
**Lung function**				
FEV_1 post (L)	4.0	2.8	1.7	0.4
FEV_1 post (% predicted)	114	94	79	17
FEV_1/FVC post	88	83	63	26
DL_{CO} (% predicted)	104	74	-	-
**Medication**				
ICS (yes/no)	no	no	no	yes
OCS (yes/no)	no	no	no	yes
SABA (yes/no)	no	no	no	no
LABA (yes/no)	no	no	no	yes
SAMA (yes/no)	no	no	no	no
LAMA (yes/no)	no	no	yes	no

FEV_1 (forced expiratory volume in 1 second); FVC (forced vital capacity); DL_{CO} (diffusing capacity of the lungs for carbon monoxide); ICS (inhaled corticosteroids); OCS (oral corticosteroids); SABA (short acting β2-agonists); LABA (long acting β2-agonists); SAMA (short acting muscarinic antagonists); LAMA (long acting muscarinic antagonists); NA (not applicable). Lung samples were excised from cancer patients and assessed as far away as possible from the tumour tissue. The tissue from GOLD IV COPD patients was from explanted lungs at the end-stage of COPD.

Supplementary Table 5 | Characteristics of COPD and control smokers peripheral lung donors

Group	No	Age (yr)	Male/ Female	Ex/current Smokers	Smoking pack-years	Chronic bronchitis (Y/N)	FEV_1 (% pred icted)	FEV_1 /FVC (%)
Patients with COPD	4	70.30 ± 6.5	4/0	3/1	96 ± 32.5	2/2	41.3 ± 26.5	48.2 ± 16.6
Control smokers	5	67.8 ± 10.3	3/2	2/3	61.2 ± 58.6	1/4	102 ± 26	81 ± 7.7

For COPD and control smoker participants, FEV_1 % predicted and FEV_1/FVC % are postbronchodilator values. Data expressed as mean ± SD. All lung resection surgery from patients with suspected lung cancer.

Supplementary Table 6 | Correlation analysis of serum anti-Smith antibody and lung function in human COPD

Patient	Age	Sex	Smoking status	FEV_1 % predicted	Average anti-Smith antibody (U/mL)	CV
**1**	63	F	Ex	31.29274	133.9	1.4
**2**	63	M	Cur	57.67217	92.6	8.9
**3**	75	M	Ex	85.05295	117.4	2.9
**4**	58	M	Ex	42.03528	158.2	2.3
**5**	73	M	Cur	57.46761	85.6	2.3
**6**	64	M	Cur	60.0486	68.2	1.0
**7**	69	F	Ex	56.38084	66.3	1.9
**8**	58	F	Ex	38.43131	127.9	2.2
**9**	61	M	Cur	41.33902	183.0	3.3
**10**	71	F	Ex	72.70775	198.7	3.4
**11**	74	F	Cur	76.54096	285.8	3.0
**12**	67	M	Cur	73.1108	228.4	7.2
**13**	66	M	Cur	26.76206	610.6	8.4
**14**	68	M	Ex	17.2032	1694.9	7.7
**15**	63	M	Ex	45.3973	626.4	0.2
**16**	81	F	Ex	38.80983	539.2	2.6
**17**	71	M	Ex	48.73073	653.9	4.1

**18**	60	F	Cur	49.66636	191.7	5.1
**19**	71	F	Ex	37.57592	131.9	9.1
**20**	70	M	Ex	40.93218	718.7	0.1
**21**	55	F	Cur	19.11071	186.0	3.7
**22**	70	M	Ex	22.56967	138.7	2.7
**23**	76	M	Ex	40.20005	278.5	8.4
**24**	66	M	Ex	23.49932	66.0	18.3
**25**	57	F	Cur	28.40203	302.6	1.5
**26**	51	M	Cur	23.88658	913.5	0.3
**27**	88	F	Ex	70.81437	129.1	2.1
**28**	69	M	Ex	40.13399	86.6	1.1
**29**	79	M	Ex	60.27518	185.8	4.3
**30**	67	F	Cur	52.44526	192.2	1.5
**31**	75	M	Ex	48.08761	146.3	0.3
**32**	62	M	Ex	31.09317	81.4	3.6
**33**	73	M	Ex	34.85255	76.2	2.6
**34**	75	M	Ex	29.19837	625.0	1.7
**35**	80	F	Ex	57.19733	187.8	4.2
**36**	61	M	Cur	51.56451	457.1	3.8

**37**	76	M	Ex	15.33453	1286.9	3.8
**38**	59	F	Ex	48.96422	1227.2	1.0
**39**	76	F	Ex	46.91628	188.6	27.5
**40**	50	M	Cur	68.95058	130.5	3.9

Abbreviations: FEV_1, forced expiratory volume in 1 second; CV, coefficient of variance; M, male; F, female; Ex, ex-smoker; Cur, current smoker

Supplementary Table 7. Patient characteristics for TLR7+ mast cell in lung tissues

	Never smoker	Smoker without COPD	COPD GOLD I	COPD GOLD II	COPD GOLD IV
Number	4	6	2	2	7
Age (years)	58	60		76	57
Sex (male/female)	3/1	3/3	2/0	1/1	5/2
Pack years (SD)	NA	42 ± 21.2	40 ± 0	22 ± 8.5	37 ± 29.4
FEV_1 (% predicted, SD)	99.3 ± 10	82.8 ± 16.3	95.5 ± 9.2	68.5 ± 16.3	22.1 ± 5.2
%LAA950 (SD)	0.6 ± 0.4	1.3 ± 1.2	13.9 ±	1.6 ± 0.6	26.8 ± 12.8

FEV_1: forced expiratory volume in 1 second;
LAA950: low attenuation areas less than a threshold of -950 Hounsfield units

Supplementary Table 8. Custom-designed primers used in qPCR analysis

Primer	Primer sequence (5' → 3')
Tlr7 forward	AGTGCCTGAAAAATGCCCTG
Tlr7 reverse	GCTCTCTGAAGAATGTCACCAC
Tnf-α forward	TCTGTCTACTGAACTTCGGGGTGA
Tnf-α reverse	TTGTCTTTGAGATCCATGCCGTT
Cxcl1 forward	GCTGGGATTCACCTCAAGAA
Cxcl1 reverse	CTTGGGGACACCTTTTAGCA
Ccl2 forward	TGAGTAGCAGCAGGTGAGTGGGG
Ccl2 reverse	TGTTCACAGTTGCCGGCTGGAG
Ccl3 forward	CTCCCAGCCAGGTGTCATTTT
Ccl3 reverse	CTTGGACCCAGGTCTCTTTGG
Ccl8 forward	GGGCCCAATGCATCCACATGC
Ccl8 reverse	TTCAGCGCAGACTTACATGCCC
Ccl12 forward	CCGGGAGCTGTGATCTTCA
Ccl12 reverse	AACCCACTTCTCGGGGT
Ccl20 forward	CGACTGTTGCCTCTCGTACA
Ccl20 reverse	AGGAGGTTCACAGCCCTTTT
Ccl22 forward	TGGCTACCCTGCGTCGTGTCCCA
Ccl22 reverse	CGTGATGGCAGAGGGTGACGG
Il-33 forward	CCTCCCTGAGTACATACAATGACC
Il-33 reverse	GTAGTAGCACCTGGTCTTGCTCTT
Mmp12 forward	CCTCGATGTGGAGTGCCCGA
Mmp12 reverse	CCTCACGCTTCATGTCCGGAG
Saa3 forward	TGATCCTGGGAGTTGACAGCCAA
Saa3 reverse	ACCCCTCCGGGCAGCATCATA
Ifna forward	SAWCYCTCCYAGACTCMTTCTGCA
Ifna reverse	TATDTCCTCACAGCCAGCAG
Ifnb forward	CCCTATGGAGATGACGGAGA
Ifnb reverse	ACCCAGTGCTGGAGAAATTG
*Ifn*g forward	GAGGAACTGGCAAAAGG
Ifng reverse	TTGCTGATGGCCTGATTGTC
Ifnl forward	CTTCAGGCCACAGCAGAGCCCAAG
Ifnl reverse	ACACACTTGAGGTCCCGGAGGA
Ifnar1 forward	CTGTGTCATGTGTGCTTCCC
Ifnar1 reverse	ATCTTTCCGTGTGCTCCTCA
Hprt forward	AGGCCAGACTTTGTTGGATTTGAA
Hprt reverse	CAACTTGCGCTCATCTTAGGATTT

Supplementary Table 9: Primer sequences of mast cell chemokines

Gene	Forward	Reverse
Ptgds	TTCAACAAGACAAGTTCCTG	GAAGGTAGAGGTGAGATTGAG
Ptges2	GAAGGACTGAGATCAAATTCTC	ATGACAGAGGAGTCATTGAG
Ltb4r1	GAAACCCTGTCCTTTTGATG	CACAGACAGTAGAACAATGG
C5a	ACATGGACCCCATAGATAAC	ACCACCGAGTAGATGATAAG
Cxcl5	CGCCGCTGGCATTTCTGTTGC	AGCTCCGTTGCGGCTATGACTG
Cxcl10	AAAAAGGTCTAAAAGGGCTC	AATTAGGACTAGCCATCCAC
Cxcl14	TGAAGAAGCTGGAAATGAAG	CCTATTCTTCGTAGACCCTG
Cxcr1	TCTGGACTAATCCTGAGGGTG	GCCTGTTGGTTATTGGAACTCTC
Cxcr2	ATGCCCTCTATTCTGCCAGAT	GTGCTCCGGTTGTATAAGATGAC
Cxcr3	TACCTTGAGGTTAGTGAACG	GGAAGGTTCTGTCAAAGTTC

Supplementary table 10: Primers sequences of TLR7 down stream molecules

Gene	Forward	Reverse
Irf1	TCTGTATAACCTACAGGTGTC	CAGACTGTTCAAAGAGCTTC
Stat1	CCCGAATTTGACAGTATGATGA	GAAGGAACAGTAGCAGGAAGGA
Myd88	TAATTGAGAAAAGGTGTCGC	ATACTGGGAAAGTCCTTCTTC
Cxcl10	AAAAAGGTCTAAAAGGGCTC	AATTAGGACTAGCCATCCAC
Oas1a	ATTAAAAAGGATGGTTCCCG	ATGTCCAGTTCTCTTCTACC

Supplementary table 11: Antibody information

Antibody name	Dilution	Catalogue #	Company
Anti-human TLR7 antibody	1:100	17232-1-AP	Proteintech
Anti-mouse TLR7 rabbit antibody	1:100	ab45371	Abcam
Anti-mouse fibronectin rabbit antibody	1: 100	ab2413	Abcam
Anti-human TLR7 antibody	1:50	MBS668010	MyBiosource
Anti-mouse TLR7 antibody	IHC: 1:100<br>WB: 1:2000	NBP2-24906	Novus Biologicals
Anti-mouse CD8	1:100	14-0808-82	ThermoFish Scientific
Anti-mouse mMCP4	1:100	ab92368	Abcam
Anti-mouse F4/80 conjugated with PE	1:50	565410	BD
Anti-human mast cell tryptase antibody	1:100	ab2378	Abcam
Anti-mouse mMCP6	1: 1000	MAB3736	R&D Systems
Anti-mouse β-actin	1: 10000	ab8226	Abcam
Anti-human Tryptase	1:75	ab196772	Abcam
Non-immune anti-mouse IgG	1:200	F031302	Agilent
Anti-rabbit IgG horseradish peroxidase conjugated antibody	1:300	ab207995	Abcam
Anti-rabbit horseradish peroxidase conjugated antibody	IHC: 1:200<br>WB:	HAF008	R&D Systems
Anti-mouse IgG antibody Alexa488®	1:100	A27023	Thermo Fisher Scientific/Invitrogen
Anti rabbit IgG antibody Alexa555®	1:100	A27036	Thermo Fisher Scientific
Anti-rabbit IgG antibody conjugated with Alexa Fluor® 488	1:100	ab150077	Abcam
Anti-rat secondary IgG conjugated with Alexa Fluor® 647	1:200	ab172335	Abcam
Anti-mouse IgG conjugated with HRP	1: 200	ab97023	Abcam
Anti-mouse IgG conjugated with HRP	1: 10000	HAF007	R&D Systems
Anti-rabbit IgG Alexa555®	1: 100	A-21428	Invitrogen

References:

1. Tilley AE*, et al.* Down-regulation of the notch pathway in human airway epithelium in association with smoking and chronic obstructive pulmonary disease. *Am J Respir Crit Care Med* **179**, 457-466 (2009).
