# Supplementary material for: TLR7 promotes smoke-induced experimental lung damage through the activity of mast cell tryptase
Source: Nat Commun. 2023 Nov 14;14:7349. doi: 10.1038/s41467-023-42913-z (PMC10646046; doi:10.1038/s41467-023-42913-z)
Supplement: Supplementary file 2 — Reporting Summary [file 41467_2023_42913_MOESM2_ESM.pdf]

nature portfolio

Corresponding author(s): Philip Hansbro

Last updated by author(s): Oct 5, 2023

Reporting Summary

Nature Portfolio wishes to improve the reproducibility of the work that we publish. This form provides structure for consistency and transparency in reporting. For further information on Nature Portfolio policies, see our Editorial Policies and the Editorial Policy Checklist.

Statistics

For all statistical analyses, confirm that the following items are present in the figure legend, table legend, main text, or Methods section.

n/a	Confirmed	
☐	☒	The exact sample size (*n*) for each experimental group/condition, given as a discrete number and unit of measurement
☐	☒	A statement on whether measurements were taken from distinct samples or whether the same sample was measured repeatedly
☐	☒	The statistical test(s) used AND whether they are one- or two-sided<br>*Only common tests should be described solely by name; describe more complex techniques in the Methods section.*
☒	☐	A description of all covariates tested
☐	☒	A description of any assumptions or corrections, such as tests of normality and adjustment for multiple comparisons
☐	☒	A full description of the statistical parameters including central tendency (e.g. means) or other basic estimates (e.g. regression coefficient) AND variation (e.g. standard deviation) or associated estimates of uncertainty (e.g. confidence intervals)
☐	☒	For null hypothesis testing, the test statistic (e.g. *F*, *t*, *r*) with confidence intervals, effect sizes, degrees of freedom and *P* value noted<br>*Give P values as exact values whenever suitable.*
☒	☐	For Bayesian analysis, information on the choice of priors and Markov chain Monte Carlo settings
☒	☐	For hierarchical and complex designs, identification of the appropriate level for tests and full reporting of outcomes
☐	☒	Estimates of effect sizes (e.g. Cohen's *d*, Pearson's *r*), indicating how they were calculated

Our web collection on statistics for biologists contains articles on many of the points above.

Software and code

Policy information about availability of computer code

Data collection	Images were collected using Image-Pro Plus software (version 6.0) to operate BX51 microscope (Olympus), ZEN desk version 3.6 software to operate Axio Imager M2 microscope (Zeiss), Metamorph software to operate BX43 microscope (Olympus). Immunoblot Images were taken using Lab 6.0.1 software to operate ChemiDoc MP System (Bio-Rad). qPCR were performed using Primer express P4 Enabler to operate the PCR instrument ABI PRISM 7000. Mouse lung function was collected by flexiWare (FX1 system) software operate flexiVent apparatus (SCIREQ). Mouse DLCO was collected using Micro GC Fusion software to operate Micro GC Fusion Gas Analyzer (INFICON).
Data analysis	Images were analysed using SigmaPlot™ software Version 14.0 or Image J 1.50.<br>RT-qPCR was analysed using QuantStudio Real Time PCR Software V1.7.2 and CFX Maestro Software 2.1<br>Statistical comparisons and graphs were anaylsed and prepared using GraphPad Prism 9.<br>Schematic figure was generated using BioRender scientific illustration software.<br>Western blot was analysed using Bio-Rad Image Lab 6.0.1.

For manuscripts utilizing custom algorithms or software that are central to the research but not yet described in published literature, software must be made available to editors and reviewers. We strongly encourage code deposition in a community repository (e.g. GitHub). See the Nature Portfolio guidelines for submitting code & software for further information.

Data

Policy information about availability of data

All manuscripts must include a data availability statement. This statement should provide the following information, where applicable:

- Accession codes, unique identifiers, or web links for publicly available datasets
- A description of any restrictions on data availability
- For clinical datasets or third party data, please ensure that the statement adheres to our policy

All the datasets in this study are existing published and are available via the NCBI website, including Gene Expression Omnibus (GSE) accession number: GSE5058 (https://www.ncbi.nlm.nih.gov/geo/query/acc.cgi?acc=GSE5058), GSE27597 (https://www.ncbi.nlm.nih.gov/geo/query/acc.cgi?acc=GSE27597), GSE8545 (https://www.ncbi.nlm.nih.gov/geo/query/acc.cgi?acc=GSE8545), GSE201465 (https://www.ncbi.nlm.nih.gov/geo/query/acc.cgi?acc=GSE201465), GSE186017 (https://www.ncbi.nlm.nih.gov/geo/query/acc.cgi?acc=GSE186017). Single cell RNA-sequencing COPD dataset is from the COPD Cell Atlas (www.copdcellatlas.com). All data are included in the Supplementary Information or are available from the authors, as are unique reagents used in this Article. The raw numbers for charts and graphs are available in the Source Data file where appropriate.

Research involving human participants, their data, or biological material

Policy information about studies with human participants or human data. See also policy information about sex, gender (identity/presentation), and sexual orientation and race, ethnicity and racism.

Reporting on sex and gender	We did not consider sex for human tissue analysis.
Reporting on race, ethnicity, or other socially relevant groupings	We did not consider race, ethnicity and other socially relevant factors in this study.
Population characteristics	For immunohistochemistry in supplementary figure 1, lung histology were obtained from never smoker (male, age: 58), smoker without COPD (female, age: 60), COPD GOLD II (female, age 76) and COPD GOLD III-IV (female, age 57).<br><br>For immunohistochemistry in Supplementary figure 2, lung samples were obtained from smoker (male n=3, female n=2, mean of age 67.8) and COPD patients (male n=4, mean of age 70.3).<br><br>For data in figure 1d-h, lung samples were from healthy donor (never smoke, male n=3, female n=1, medium of age 58), smoker without COPD (male n=3, female n=3, medium of age 60) and COPD I-II (male n=3, female =1, medium of age =76) and COPD IV (male n=5, female n=2, medium of age 57).
Recruitment	Patients were randomly recruited from outpatient, inpatient and were eligible to participate if there were >18 years of age. Lung tissues for immunofluorescence: All patients had complete medical history and lung function tests (spirometry); they also underwent whole-lung volumetric computed tomography (CT) at study entry and at Year 5, permitting longitudinal assessment of lung CT characteristics, expressed in %LAA950 scores. For lung Biorepository, donors either underwent surgery for solitary pulmonary nodule removal and the lung samples were collected 10 cm away from the nodule, or underwent lung transplant for severe COPD.<br><br>Lung tissues for immunohistochemistry: Lung samples were excised from healthy donor, cancer patients that assessed as far away as possible from the tumour tissue. The tissue from GOLD IV COPD patients was from explanted lungs at the end-stage of COPD.
Ethics oversight	The Institutional Review Board of the University of Arizona approved patient recruitment protocol (2019-2021).<br>The human study was also approved by the medical ethics committee of the Ghent University Hospital (2016/0312 and 2019/0537) and the "delibera C.S. n.270 of the 18th of October 2017" of the Ethic Committee of the Academic Hospital of Messina, Italy (www.polime.it).<br>All recruited volunteers provided written informed consent.

Note that full information on the approval of the study protocol must also be provided in the manuscript.

Field-specific reporting

Please select the one below that is the best fit for your research. If you are not sure, read the appropriate sections before making your selection.

☒ Life sciences ☐ Behavioural & social sciences ☐ Ecological, evolutionary & environmental sciences

For a reference copy of the document with all sections, see nature.com/documents/nr-reporting-summary-flat.pdf

Life sciences study design

All studies must disclose on these points even when the disclosure is negative.

Sample size	For in vivo experiments, to achieve 20-30% differences in groups with a confidence of 95% experimental groups, we use minimum 6 mice per group in this study based on G*Power Data analysis of two-tailed t-test between 2 groups (alpha=0.05, power =0.8).

	For in vitro experiment, sample size was guided by standards in the field according to our previous publications (PMID: 35777766, 31343988). Cell culture samples was in triplicated in each experiment ,and it repeated 3-6 times at different time points.
Data exclusions	No data exclusions in this study.
Replication	Animal experiments were independently performed for at least two times with more than two replicated. In vitro data have been performed independently for at least three times with three replicated. All attempts at replication were successful.
Randomization	Animal matched with sex and age were randomly assigned into each experimental group. for In vitro studies, cells or conditions were assigned randomly to each experimental group
Blinding	Animal identification code was assigned to each mice that the investigator were not blinded to group allocation during data collection. For in vitro experiment, the investigators were blinded to group allocation during data collection and analysis.

Reporting for specific materials, systems and methods

We require information from authors about some types of materials, experimental systems and methods used in many studies. Here, indicate whether each material, system or method listed is relevant to your study. If you are not sure if a list item applies to your research, read the appropriate section before selecting a response.

Materials & experimental systems

n/a	Involved in the study
☐	☒ Antibodies
☐	☒ Eukaryotic cell lines
☒	☐ Palaeontology and archaeology
☐	☒ Animals and other organisms
☒	☐ Clinical data
☒	☐ Dual use research of concern
☒	☐ Plants

Methods

n/a	Involved in the study
☒	☐ ChIP-seq
☒	☐ Flow cytometry
☒	☐ MRI-based neuroimaging

Antibodies

Antibodies used

All antibodies in this study for immunoblot, immunofluorescence, immunohistochemistry and in vivo experiment are listed below:
Primary antibodies:
Anti-human TLR7 antibody (Cat: 17232-1-AP, Proteintech)
Anti-mouse TLR7 rabbit antibody (Cat: ab45371, Abcam)
Anti-mouse fibronectin rabbit antibody (Cat: ab2413, Abcam)
Anti-human TLR7 (Cat: MBS668010, MyBiosource)
Anti-mouse TLR7 (Cat: NBP2-24906, Novus Biologicals)
Anti-mouse CD8 (Cat:14-0808-82, ThermoFish Scientific)
Anti-mouse mMCP4 (Cat: ab92368, Abcam)
Anti-mouse F4/80 conjugated with PE (Cat: 565410, BD)
Anti-human mast cell tryptase antibody (Cat: ab2378, Abcam)
Anti-mouse mMCP6(Cat: MAB3736, R&D Systems)
Anti-mouse-actin (Cat: ab8226, Abcam)
Anti-human Tryptase (Cat: ab196772, Abcam)

Secondary antibodies:
Non-immune anti-mouse IgG (Cat: F031302, Agilent)
Anti-rabbit IgG (Cat: ab207995, Abcam)
Anti-rabbit horseradish peroxidase conjugated antibody (Cat: HAF008, R&D Systems)
Anti-mouse IgG antibody Alexa488® (Cat:A27023, Thermo Fisher Scientific)
Anti rabbit IgG antibody Alexa555® (Cat:A27036, Thermo Fisher Scientific)
Anti-rabbit IgG antibody conjugated with Alexa Fluor® 488 (ab150077, Abcam)
Anti-rat secondary IgG conjugated with Alexa Fluor® 647 (Cat: ab172335, Abcam)
Anti-mouse IgG conjugated with HRP (Cat: ab97023, Abcam)
Anti-mouse IgG conjugated with HRP (Cat: HAF007, R&D Systems)
Anti-rabbit IgG Alexa555® (Cat: A-21428, Invitrogen)

For in vivo experiment:
Neutralizing anti-TLR7 (clone Ba/F3 monoclonal antibody and IgG1/κ isotype control were obtained from Miyake lab.

Validation

Anti-human TLR7 antibody (Cat: 17232-1-AP, Proteintech): https://www.ptglab.com/products/TLR7-Antibody-17232-1-AP.htm
Anti-mouse TLR7 rabbit antibody (Cat: ab45371, Abcam): https://www.abcam.com/products/primary-antibodies/tlr7-antibody-ab45371.html

Anti-mouse fibronectin rabbit antibody (Cat: ab2413, Abcam): https://www.abcam.com/products/primary-antibodies/fibronectin-antibody-ab2413.html
Anti-human TLR7 (Cat: MBS668010, MyBiosource): https://www.mybiosource.com/TLR7-antibody
Anti-mouse TLR7 (Cat: NBP2-24906, Novus Biologicals): https://www.novusbio.com/products/tlr7-antibody_nbp2-24906
Anti-mouse CD8 (Cat:14-0808-82, ThermoFish Scientific): https://www.thermofisher.com/antibody/product/CD8a-Antibody-clone-4SM15-Monoclonal/14-0808-82
Anti-mouse mMCP4 (Cat: ab92368, Abcam): https://www.abcam.com/products/primary-antibodies/mmcp-4-antibody-ab92368.html
Anti-mouse F4/80 conjugated with PE (Cat: 565410, BD): https://www.bdbiosciences.com/en-au/products/reagents/flow-cytometry-reagents/research-reagents/single-color-antibodies-ruo/pe-rat-anti-mouse-f4-80.565410
Anti-human mast cell tryptase antibody (Cat: ab2378, Abcam):
https://www.abcam.com/products/primary-antibodies/mast-cell-tryptase-antibody-aa1-ab2378.html
Anti-mouse mMCP6(Cat: MAB3736, R&D Systems):
https://www.rndsystems.com/products/mouse-mast-cell-protease-6-mcpt6-antibody-286820_mab3736
Anti-mouse-actin (Cat: ab8226, Abcam):
https://www.abcam.com/products/primary-antibodies/beta-actin-antibody-mabcam-8226-loading-control-ab8226.html
Anti-human Tryptase (Cat: ab196772, Abcam):
https://www.abcam.com/products/primary-antibodies/mast-cell-tryptase-antibody-ab196772.html
Non-immune anti-mouse IgG (Cat: F031302, Agilent): https://www.agilent.com/en/product/clinical-flow-cytometry/reagents-for-clinical-flow-cytometry/clinical-secondary-antibodies/rabbit-anti-mouse-igg-981967
Anti-rabbit IgG (Cat: ab207995, Abcam): https://www.abcam.com/products/secondary-antibodies/goat-rabbit-igg-hl-biotin-ab207995.html
Anti-rabbit horseradish peroxidase conjugated antibody (Cat: HAF008, R&D Systems): https://www.rndsystems.com/products/rabbit-igg-horseradish-peroxidase-conjugated-antibody_haf008
Anti-mouse IgG antibody Alexa488® (Cat:A27023, Thermo Fisher Scientific): https://www.thermofisher.com/antibody/product/Rabbit-anti-Mouse-IgG-H-L-Secondary-Antibody-Recombinant-Polyclonal/A27023
Anti rabbit IgG antibody Alexa555® (Cat:A27036, Thermo Fisher Scientific):
https://www.thermofisher.com/antibody/product/Goat-anti-Rabbit-IgG-Heavy-chain-Secondary-Antibody-Recombinant-Polyclonal/A27039
Anti-rabbit IgG antibody conjugated with Alexa Fluor® 488 (ab150077, Abcam): https://www.abcam.com/products/secondary-antibodies/goat-rabbit-igg-hl-alexa-fluor-488-ab150077.html
Anti-rat secondary IgG conjugated with Alexa Fluor® 647 (Cat: ab172335, Abcam): https://www.abcam.com/products/secondary-antibodies/mouse-monoclonal-2b-10a8-rat-igg2b-heavy-chain-alexa-fluor-647-ab172335.html
Anti-mouse IgG conjugated with HRP (Cat: ab97023, Abcam): https://www.abcam.com/products/secondary-antibodies/goat-mouse-igg-hl-hrp-ab97023.html
Anti-mouse IgG conjugated with HRP (Cat: HAF007, R&D Systems):
https://www.rndsystems.com/products/mouse-igg-horseradish-peroxidase-conjugated-antibody_haf007
Anti-rabbit IgG Alexa555® (Cat: A-21428, Invitrogen): https://www.thermofisher.com/antibody/product/Goat-anti-Rabbit-IgG-H-L-Cross-Adsorbed-Secondary-Antibody-Polyclonal/A-21428
Neutralizing anti-TLR7 (clone Ba/F3 monoclonal antibody and IgG1/κ isotype control were obtained from Miyake lab. The generation of the antibodies refers to previous publication (PMID: 23446849).

Eukaryotic cell lines

Policy information about cell lines and Sex and Gender in Research

Cell line source(s)	Human mast cell line (HMC)-1 was sourced from ATCC.<br>Bone marrow derived mast cells were collected and cultured from Wild-type (WT) and toll-like receptor (TLR)7-deficient (-/-) mice and the protocols were according to our previous publications [PMID:35777766].
Authentication	The cell line was authenticated by morphology and identified with mast cell specific markers.
Mycoplasma contamination	The cells were tested negative for mycoplasma
Commonly misidentified lines<br>(See ICLAC register)	No commonly misidentified cell lines were used in this study

Animals and other research organisms

Policy information about studies involving animals; ARRIVE guidelines recommended for reporting animal research, and Sex and Gender in Research

Laboratory animals	All the mice in this study are 7-8-week-old. WT controls, Tlr7-/- BALB/c mice and Myd88-/- BALB/c mice were originally from Prof. Shizhu Akirap at Osaka University, Japan and provided to the Matters' lab at the University of Newcastle, Australia. Mouse mast cell protease-6-deficient (mmcp6-/-) C57BL/6 mice and protease serine member S31-deficient (Prss31-/-) C57BL/6 mice were a gift from Prof. Rick Steven at Brigham and Women's Hospital, Harvard University and were then maintained at Australian BioResources facility WT BALB/c mice were used as control for Tlr7-/- BALB/c mice, and WT C57BL/6 mice were used as controls for mmcp6-/- and Prss31-/- mice.
Wild animals	This study did not involve wild animals.

Reporting on sex	Female mice were used in this study.
Field-collected samples	No field collected samples used.
Ethics oversight	Animal works in this study was performed in accordance with the recommendations issued in the Australian code of practice for the care and use of animals for scientific purposes by the National Health and Medical Research Council of Australia. All protocols were approved by the Animal Ethics Committee of The University of Newcastle, Australia (A-2008-100) and Sydney Local Health District (SLHD) Animal Ethics and Welfare Committee (2018/004).

Note that full information on the approval of the study protocol must also be provided in the manuscript.
